# Mechanochemical Activation of Zinc and Application to Negishi Cross‐Coupling

**DOI:** 10.1002/anie.201806480

**Published:** 2018-08-02

**Authors:** Qun Cao, Joseph L. Howard, Emilie Wheatley, Duncan L. Browne

**Affiliations:** ^1^ School of Chemistry Cardiff University Main Building, Park Place Cardiff CF10 3EQ UK

**Keywords:** ball milling, mechanochemistry, organometallic, organozinc, solventless reactions

## Abstract

A form independent activation of zinc, concomitant generation of organozinc species and engagement in a Negishi cross‐coupling reaction via mechanochemical methods is reported. The reported method exhibits a broad substrate scope for both C(sp^3^)–C(sp^2^) and C(sp^2^)–C(sp^2^) couplings and is tolerant to many important functional groups. The method may offer broad reaching opportunities for the in situ generation organometallic compounds from base metals and their concomitant engagement in synthetic reactions via mechanochemical methods.

The controlled and selective synthesis of carbon based molecules is a critical endeavor that is key to the discovery of new medicines, crop protection agents, flavors, and fragrances as well as many more materials with great importance to human quality of life. Whilst synthetic chemistry as a discipline has a good grasp on making molecules succumb to synthesis, focus in the modern era is centered upon achieving sustainable synthesis through reduced numbers of reaction steps, reduced quantities of waste, and milder reaction conditions.^1^ Many of these facets can be achieved by exploring reaction technologies such as photo‐ or electrochemistry, which are complementary to traditional methods and allow controlled access to reaction manifolds that were previously unobtainable. Recently, we and others have been exploring mechanochemistry as a method to complement the synthetic toolkit.^2^ The method of solid state grinding or milling using electronically‐powered devices; mills, is attractive as it a) negates the requirement for bulk solvent use during the reaction step and b) provides a reproducible and sustainable energy input in comparison to a human operated mortar and pestle. Indeed, the crystal engineering and metal‐organic‐framework communities are far ahead in exploring the potential of this technique, and have uncovered a wealth of opportunities, including reduced reaction times, increased “space‐time yields”, new polymorphic forms, liquid assisted grinding and the use of grinding auxiliaries or “glidants”.^3^ As applied to organic synthesis there are already several examples of reduced reaction times, altered chemo‐selectivity and the ability to synthesize products that were previously unobtainable.2a However, when and how these observations will arise is currently not predictable.

Herein we report on the use of mechanochemistry as a technique to enable the form independent preparation of organozinc species, from the base metal zinc, and their engagement in mechanochemical Negishi cross‐coupling reactions. The method is operationally simple and requires no use of inert gases but is instead conducted in air. The reported method offers broad reaching opportunities for the in situ generation and use of organometallic compounds in synthesis from their base metals. The late‐stage modification of organic materials by metal mediated carbon‐carbon bond formation is a ubiquitous strategy for the discovery of new or improved chemicals in many sectors.^4^ Key to the adoption of any developed late‐stage technique is the breadth of substrate scope and application, in this regard, organozinc species are privileged compounds representing a class of organometallic reagents with excellent functional group compatibility. Unlike boronic acids, esters and boronates, organozinc reagents are not widely available commercially and must be prepared at the point of use. Owing to this limitation, the Negishi cross‐coupling reaction is under‐utilised.^5^ This problem is further compounded by the tedious preparation of organozinc species, which can be achieved by several methods (Scheme 1). Metallation of C−H bonds can be achieved through a transmetallation method, whereby direct deprotonation or directed‐*ortho*‐metallation with organomagnesium or organolithium provides the initial metalation and is followed by transmetallation to a zinc (II) species (A, Scheme 1).^6^ Indeed, such an approach often leads to a loss of the broad functional group tolerance afforded by organozinc reagents because they have been prepared via more reactive organometallic species. Knochel and co‐workers realized a solution to this through direct zincation of C−H bonds using zinc‐amide bases.^7^

**Scheme 1:**
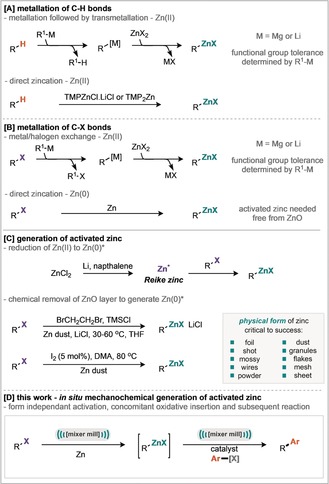
Formation of Organozinc reagents.

An alternative tactic involves the metalation of C−X bonds (B, Scheme 1), a strategy where metal‐halogen exchange is particularly popular, but again such an approach leads to a loss of the broad functional group tolerance afforded by organozinc reagents.^8^ More attractively, for C−X bonds, is the direct oxidative insertion of Zn^0^ from an activated zinc metal. Indeed, highly reactive Rieke zinc can be formed by the reduction of ZnCl_2_ using alkali metals and naphthalene, and will undergo oxidative addition with an alkyl or aryl halide to generate the desired organozinc species (C, Scheme 1).^9^ A complementary, and more popular approach to generate activated zinc metal is to remove zinc oxide from the metal surface by chemical reaction or entrainment, typically achieved using chemical additives such as TMSCl, 1,2‐dibromoethane, bromine, or iodine.^10^, ^11^ All of the methods described for preparing activated zinc require an inert atmosphere, and those derived from zinc metal are highly dependent on the physical form of zinc used, with multiple different forms of zinc commercially available (Scheme 2, and photograph in the Supporting Information). We have identified mechanical activation, by ball‐milling, as a general technique that could simplify and enable organometallic chemistry by obviating the need for strictly dry solvents and specific base metal forms whilst providing greater reproducibility in the generation of these important materials.10k With regards to zinc chemistry we sought to probe this hypothesis and develop a method to generate and subsequently use organozinc reagents (D, Scheme 1). Studies commenced by treating ethyl‐4‐bromobutanoate to grinding in the presence of granular zinc (20–30 mesh). Optimized conditions consisted of milling with 1.1 equivalents of zinc for 4 hours with 1.5 equivalents of DMA which afforded 76 % yield of the dehalogenated ethylbutanoate (**3**) after an acidic quench of the jar contents.^12^, ^17^ These conditions were then applied to a further 11 commercially available zinc forms (Scheme 2). Notably, these forms vary in their particle size, and consequently both their surface area to volume ratios and zinc oxide to zinc metal ratio. There appears to be a general trend that the forms with a lower surface area to volume ratio perform better, likely attributable to the increased amount of inactive zinc oxide in the forms with higher surface area to volume ratios. For example, puriss, shot, and mossy perform better than powder, flake, and dust. Having established the conditions required to generate organozinc reagents directly, the subsequent Negishi coupling was investigated in a one‐pot fashion.^13^ Initial reaction conditions used bromobenzene as the coupling partner and the Pd‐PEPPSI family of catalysts, which have been reported to possess high stabilities and exhibit good reactivity for the Negishi coupling.^14^ Tetrabutylammonium salts were explored as additives for the mechanochemical Negishi coupling reaction (Scheme 3, entries 3–5), with tetrabutylammonium bromide (TBAB) providing the best result with a 70 % yield.^15^ A range of polar aprotic additives were also investigated in the first step, this confirmed DMA as the most effective, with DMF and NMP performing similarly.

**Scheme 2:**
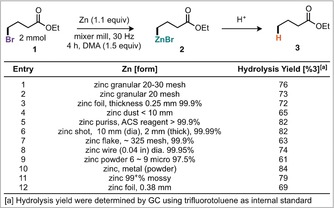
Mechanochemical preparation of organozinc reagents.

**Scheme 3:**
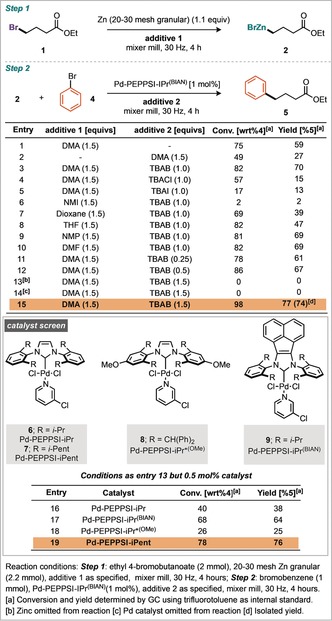
Optimization of one‐jar, two‐step reaction.

Increasing the quantity of TBAB to 1.5 equivalents further improved the yield to 77 % (GC) corresponding to a 74 % isolated yield (Scheme 3, entry 15). Notably, omission of zinc (Scheme 3, entry 13) or omission of palladium catalyst (Scheme 3, entry 14) from the reaction resulted in none of the desired product suggesting an innocent role of the grinding vessels and media.^16^ Finally a screen of four different Pd‐PEPPSI catalysts was undertaken, with Pd‐PEPPSI‐*i*Pent determined to be the most effective catalyst under these milling conditions (Scheme 3, Entry 17). With established conditions for the mechanochemical in situ synthesis of an organozinc species and subsequent Negishi coupling in a one‐pot two‐step manner in hand, the applicability of these conditions to a range of different substrates was explored (Scheme 4). Initially, formation of organozincs from sp^3^ hybridized organobromides and subsequent coupling with sp^2^ hybridized C−X coupling partners was investigated. It was found that chloro‐, bromo‐, and iodoarenes were successfully transformed in good yields. The excellent functional group tolerance exhibited by organozinc reagents is demonstrated, with molecules containing esters, nitriles, and ketones achieving high yields. Structures primed for further derivatization were also synthesized, such as boronate deriative **14**, which could potentially undergo subsequent Suzuki–Miyaura coupling. Orthogonal coupling, with selectivity between the C−Br and C−OTs was also demonstrated through the preparation of tosylate **12**. Secondary organozinc reagents were formed and reacted successfully, as exemplified by preparation of the cyclohexyl derivatives **15** and **17**. Electron‐rich aromatics also participated in the reaction process to furnish dimethoxy derivative **21** and thiofuran compound **22**, albeit in moderate yields. The possibility of sp^2^–sp^2^ coupling was investigated under similar conditions (Scheme 4).^17^ With these conditions a number of biaryl products were successfully synthesized in good yields. Sterically demanding *ortho*‐substituted bromoaryls were also competent coupling partners (**31, 35**, and **39**). Thiophenes (**33, 34**, and **40**) and a pyridine (**41**) derivative were also prepared, highlighting the applicability of this method to synthesize substituted heterocycles.

**Scheme 4:**
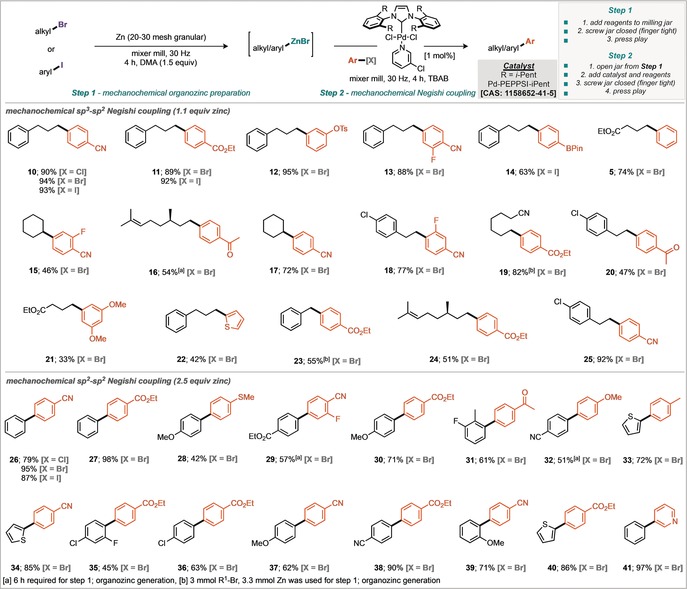
Scope of the mechanochemical Negishi reaction.

Encouraged by the success of this one‐pot two step procedure, we sought to push this methodology further and explore a one‐pot, one step protocol, whereby the organozinc reagent would be generated and consumed through palladium catalysis all within the same reaction jar.^18^ To bias the system against complex mixtures, we took advantage of the fact that alkyl organozincs form more readily than aryl organozinc species.9d Thus dosing both alkyl and aryl halide coupling partners with zinc, DMA, and Pd‐PEPPSI‐*i*Pent into the grinding jar along with the grinding ball and grinding for 8 hours afforded the desired sp^3^–sp^2^ bond formation (Scheme 5). Excellent yields were obtained for the three examples explored, demonstrating the ability to perform Negishi coupling without directly handling organozinc reagents. Moreover, this has been demonstrated with three different forms of zinc; 20–30 mesh granular, powder and puriss, all of which lead to the desired products in excellent yields.

**Scheme 5:**
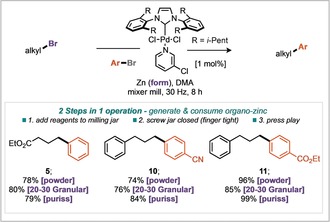
One‐pot, one‐step Negishi reaction.

In summary, we have developed a novel method for the synthesis and subsequent reaction of organozinc species under mechanochemical conditions, without the need to use inert atmosphere techniques or dry solvents. Organozincs can be generated from an alkyl halide irrespective of the physical form of commercially available zinc metal. A coupling partner could then be added to the reaction mixture along with a palladium catalyst and TBAB to perform the Negishi reaction in a one‐pot, two‐step process. Most excitingly, these conditions were successfully modified to realize the direct generation and consumption of organozinc reagents through a one‐pot Negishi coupling process for the synthesis C(sp^3^)–C(sp^2^) bonds thus rendering it more operationally simplistic than current procedures.^19^ The application of this technique to the generation and use of other organometallic species is currently underway in our laboratories.

## Conflict of interest

The authors declare no conflict of interest.

## Supporting information

As a service to our authors and readers, this journal provides supporting information supplied by the authors. Such materials are peer reviewed and may be re‐organized for online delivery, but are not copy‐edited or typeset. Technical support issues arising from supporting information (other than missing files) should be addressed to the authors.

Supplementary
