# Supplementary material for: Mechanochemical Activation of Zinc and Application to Negishi Cross‐Coupling
Source: Angew Chem Int Ed Engl. 2018 Aug 2;57(35):11339–43. doi: 10.1002/anie.201806480 (PMC6220771; doi:10.1002/anie.201806480)
Supplement: Supplementary file 1 — Supplementary [file ANIE-57-11339-s001.pdf]

Angewandte Chemie
Eine Zeitschrift der Gesellschaft Deutscher Chemiker

Supporting Information

Mechanochemical Activation of Zinc and Application to Negishi Cross-Coupling

*Qun Cao, Joseph L. Howard, Emilie Wheatley, and Duncan L. Browne**

anie_201806480_sm_miscellaneous_information.pdf

Supporting Information

Table of Contents

1. General Information..S2
2. Experimental Procedures S4
3. Mechanochemical Organozinc Generation....……………………………………….......S7
4. Characterization Data for Ligands and Pd(II) Complexes.......................................S10
5. Product Characterization Data……………………………………………………….S16
6. NMR data...S28
7. References...S77

1 General Information

Unless otherwise stated, all reagents were purchased from commercial source and used without further purification. The following palladium salts were purchased from Sigma Aldrich: palladium(II) acetate (≥99.9% trace metal basis), palladium(II) chloride (≥99.9%), Pd-PEPPSI-IPent catalyst (**7**) (≥95%). *N,N*-Dimethylacetamide (DMA, anhydrous 99.8%), *N,N*-dimethylformamide (DMF, anhydrous, 99.8%), 1-methyl-2-pyrrolidinone (NMP, anhydrous, 99.5%), 1-methylimidazole (NMI, 99%), tetrahydrofuran (THF, anhydrous, ≥99.9%), 1,4-dioxane (anhydrous, 99.8%) were purchased from Sigma Aldrich. Different zinc forms were purchased from different companies as listed below: (1) Zinc granular (20-30 mesh, ACS reagent, ≥98.8%; Sigma-Aldrich). (2) Zinc granular (20 mesh, Sigma-Aldrich). (3) Zinc foil (thickness 0.25 mm, 99.9%; Sigma-Aldrich). (4) Zinc dust (< 10 µm, ≥98%; Sigma-Aldrich). (5) Zinc puriss.p.a. (ACS reagent, ≥99.9%; Sigma-Aldrich). (6) Zinc shot, (10 mm (0.4 in) dia x 2 mm (0.08 in) thick, 99.99%; Alfa Aesar) (7) Zinc flake (-325 mesh, 99,9%; Alfa Aesar). (8) Zinc wire (1.0 mm (0.04 in) dia, 99.95%; Alfa Aesar). (9) Zinc powder (average 6-9 micro, 97.5%; Alfa Aesar). (10) Zinc Powder (Certified AR for Analysis, metal; Fisher Chemical). (11) Zinc mossy (+99%, ACROS Organics). (12) Zinc foil (about 0.38 mm, Fisher Scientific).

Thin layer chromatography (TLC) was carried out using Merck TLC silica gel 60 sheet, and visualized with ultraviolet light or potassium permanganate stain. Flash column chromatography (FCC) was performed with Sigma Aldrich silica gel 40-60 Å as the stationary phase and solvents employed were analytical grade. ^{1}H NMR spectra were recorded on a Bruker AVX500 (500 MHz) spectrometer at ambient temperature. ^{13}C NMR spectra were recorded on a Bruker AVX500 (125 MHz) spectrometer at ambient temperature. ^{19}F NMR spectra were recorded on a Bruker AVX500 (471 MHz) spectrometer at ambient temperature. Melting points were measured on a Gallenkamp melting point apparatus and are reported corrected by linear calibration to benzophenone (47 - 49 °C) and benzoic acid (121 - 123 °C).

High resolution mass spectroscopy (HRMS) data was obtained on a Thermo Scientific LTQ Orbitrap XL by the EPSRC UK National Mass Spectrometry Facility at Swansea University or on a Waters MALDI-TOF mx in Cardiff University. Spectra were obtained using electron impact ionization (EI), chemical ionization (CI), positive electrospray (ES), pneumatically-assisted electrospray (pNSI) or atmospheric solids analysis probe (ASAP+). Infrared spectra were recorded on a Shimadzu IR-Affinity-1S FTIR spectrometer.

Gas chromatography analysis was carried out using a Bruker Scion 456 gas chromatograph. An Agilent 19091J-413HP-5 column (30.0 m × 320 μm × 0.25 μm nominal) was employed for all of the separations using the following conditions: initial column temperature, 40 °C; initial hold time, 2 min; next temperature, 100 °C; hold time, 5 min; rate of temperature ramp 1, 4 °C/min, final temperature 300 °C; hold time, 5 min; rate of temperature ramp 2, 15 °C/min; injection temperature, 250 °C; injection volume 1 μL; detection temperature, 300 °C, split mode. The effluent was combusted in an H_2/air flame and detected using FID (flame ionization detector).

The ball mill used was a Retsch MM 400 mixer mill. Unless otherwise stated, mechanochemical reactions were performed in 10 mL stainless steel jars from Retsch with one stainless steel ball of mass 4 g. The longest time that this mill can be programmed to run for is 99 minutes. In order to run longer reaction times the mill was started, and then additional time added to the timer in order to ensure that the mill was running continuously for the desired reaction time.

The GC yield of products and conversion of substrates were determined by using the internal standard method. The response factor (RF) of analytes was determined by analyzing known quantities of internal standard (trifluorotoluene) against known quantities of substrate and product:

$$\mathrm{RF} = \frac{\mathrm{Area}_{\mathrm{internal\ standard}} \times \mathrm{Moles}_{\mathrm{analyte}}}{\mathrm{Area}_{\mathrm{analyte}} \times \mathrm{Moles}_{\mathrm{internal\ standard}}}$$

The quantity of an analyte was then calculated according to the following equation:

$$\mathrm{Moles}_{\mathrm{analyte}} = \frac{\mathrm{RF} \times \mathrm{Moles}_{\mathrm{internal\ standard}} \times \mathrm{Area}_{\mathrm{analyte}}}{\mathrm{Area}_{\mathrm{internal\ standard}}}$$

2 Experimental Procedures

General method for evaluation of types of zinc, additives and time for mechanochemical organozinc generation

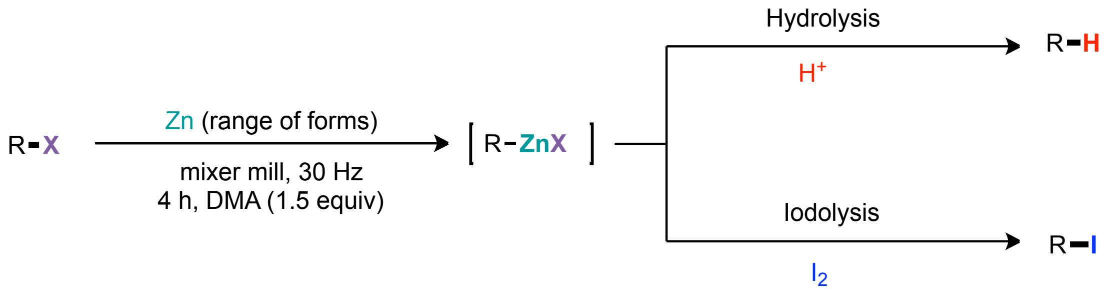

To a 10 mL Retsch stainless steel milling jar was added the organohalide (2 mmol), zinc (2.2 mmol, 0.148 g), and DMA (3 mmol, 0.261 g) under air atmosphere. A stainless steel ball of mass 4.0 g was added and the mixture was milled at 30 Hz for 4 hours.

(1) For analysis of organohalide conversion and its corresponding hydrolyzed product:

After the reaction was finished, the resulting grey color paste was transferred to a 50 mL flask, washing the residue with dichloromethane (20 mL). Internal standard trifluorotoluene (0.041 mL, 0.33 mmol) and 2 M HCl solution (10 mL) were added and the mixture was stirred for 20 min to hydrolyze any organozinc. The organic layer was washed through a pipette containing a small plug of silica gel using diethyl ether as eluent to remove leftover salts. The filtrate was collected and submitted for GC analysis.

(2) For analysis organozinc yield using iodolysis under air:

The iodolysis method used here was adjusted from the reported literature.[1] Anhydrous LiCl (0.636 g, 15 mmol) was added in a N_2 flushed 50 mL round bottom flask and dried under high vacuum for 4 hours at 140 °C. After the flask was cooled to room temperature, anhydrous THF (30 mL) was added and stirred for 4 hours to fully dissolve LiCl under N_2, resulting in a 0.5 M solution of LiCl in THF. Then to an oven dried flask, iodine (0.635 g, 2.5 mmol) was added and dissolved in 4 mL premade LiCl solution (0.5 M) under N_2 gas protection.

After the mechanochemical organozinc generation was finished, 4 mL of the previously made iodine solution was added immediately via syringe to the mix mill jar under air and the jar was shaken by hand for 1 min to allow iodine to fully react with the organozinc. Then Internal standard trifluorotoluene (0.041 mL, 0.33 mmol) was then added. Then the collected organic solution was washed through a silica plug with diethyl ether to remove salt. The filtrate was collected and submitted for GC analysis.

General Method 1: one-jar two-step Negishi cross coupling using mechanochemically generated organozinc

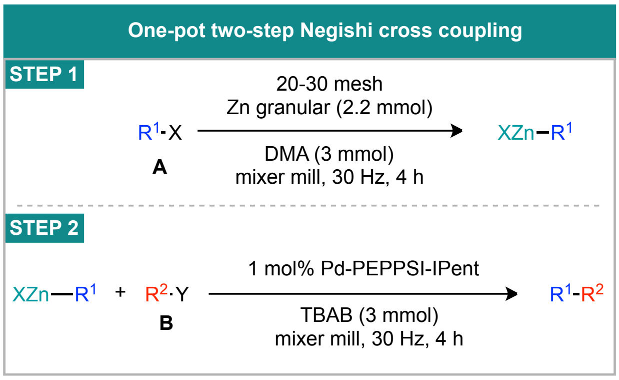

To a Retsch 10 mL stainless steel milling jar was added the organohalide **A** (2 mmol), Zn granular (20 - 30 mesh, 2.2 mmol, 0.148 g), and dimethylacetamide (3 mmol, 0.261 g) under air atmosphere. A stainless steel ball of mass 4.0 g was added and the mixture was milled at 30 Hz for 4 hours. After the formation of organozinc reagent under mechanochemical conditions, the milling jar was opened under air. Subsequently, TBAB (3 mmol, 0.967 g), Pd-PEPPSI-IPent (0.01 mmol, 0.008 g), and organohalide **B** (1 mmol) were added. Then the milling jar was closed and the reaction mixture was milled at 30 Hz for 4 hours. When the reaction was finished, the resulting grey color paste was transferred into a 50 mL flask by washing with dichloromethane (20 mL). Then 2 M HCl solution (10 mL) was added and the mixture was stirred for 20 min quench unreacted zinc and organozinc. Then the reaction mixture was transferred to a separating funnel. The aqueous layer was separated and back extracted with ethyl acetate (30 mL) twice. Then the combined organic layers were then dried over $MgSO_4$, filtered and concentrated under reduced pressure. The crude material was then purified by silica gel flash chromatography using the noted solvent systems.

Note: For the generation of C(Sp^2) organozinc using aryl hailides, Zn granular (20-30 mesh, 5 mmol, 0.327 g) was used. For cross coupling product bearing heteroaryl or acid sensitive groups, deionised water should be use to hydrolyze the leftover organozincs rather than 2 M HCl solution.

General Method 2: one-jar one-step Negishi cross coupling using mechanochemically generated organozinc

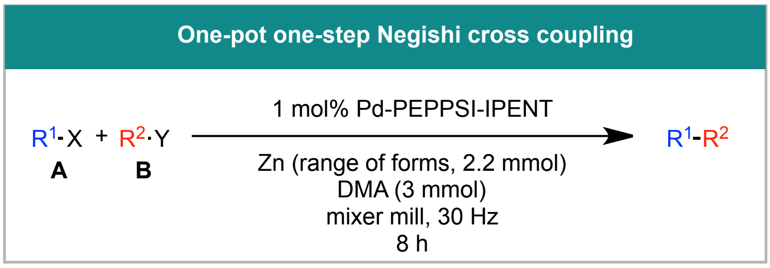

To a Retsch 10 mL stainless steel milling jar was added the organohalide **A** (2 mmol), the zinc (powder/granular/puriss, 2.2 mmol, 0.148 g), dimethylacetamide (3 mmol, 0.261 g), Pd-PEPPSI-IPent (0.01 mmol, 0.008 g), and organohalide **B** (1 mmol) under air atmosphere. A stainless steel ball of mass 4.0 g was added and the mixture was milled at 30 Hz for 8 hours. When reaction was finished, the milling jar was opened under air. When the reaction was finished, the resulting grey color paste was transferred into a 50 mL flask by washing with dichloromethane (20 mL). Then 2 M HCl solution (10 mL) was added and the mixture was stirred for 20 min to quench unreacted zinc and organozinc. The reaction mixture was then transferred to a separating funnel, the aqueous layer was separated and back extracted with ethyl acetate (30 mL) twice. The combined organic layers were dried over $MgSO_4$, filtered and concentrated under reduced pressure. The crude material was then purified by silica gel flash chromatography using the noted solvent systems.

3 Mechanochemical Organozinc Generation

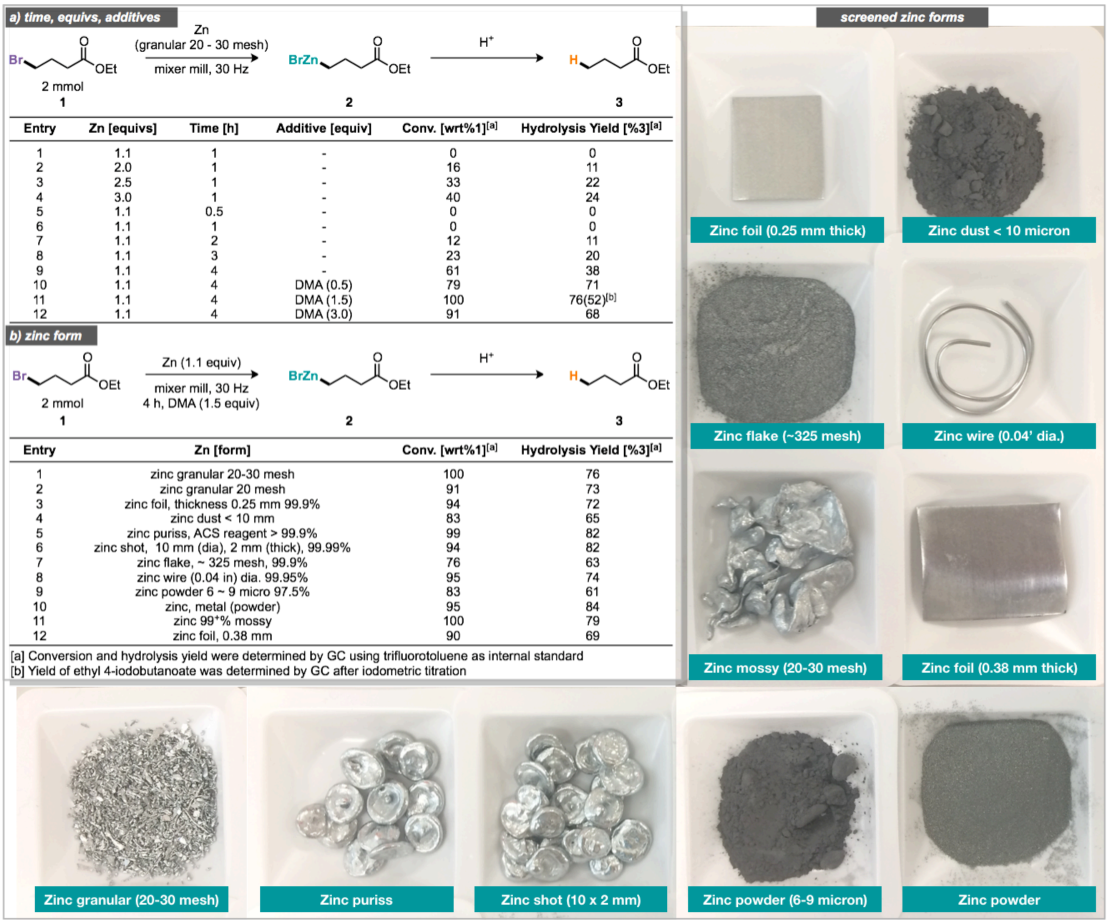

Entry	Zn [equivs]	Time [h]	Additive [equiv]	Conv. [wrt%1][a]	Hydrolysis Yield [%3][a]
1	1.1	1	-	0	0
2	2.0	1	-	16	11
3	2.5	1	-	33	22
4	3.0	1	-	40	24
5	1.1	0.5	-	0	0
6	1.1	1	-	0	0
7	1.1	2	-	12	11
8	1.1	3	-	23	20
9	1.1	4	-	61	38
10	1.1	4	DMA (0.5)	79	71
11	1.1	4	DMA (1.5)	100	76(52)[b]
12	1.1	4	DMA (3.0)	91	68

Entry	Zn [form]	Conv. [wrt%1][a]	Hydrolysis Yield [%3][a]
1	zinc granular 20-30 mesh	100	76
2	zinc granular 20 mesh	91	73
3	zinc foil, thickness 0.25 mm 99.9%	94	72
4	zinc dust < 10 mm	83	65
5	zinc puriss, ACS reagent > 99.9%	99	82
6	zinc shot, 10 mm (dia), 2 mm (thick), 99.99%	94	82
7	zinc flake, ~ 325 mesh, 99.9%	76	63
8	zinc wire (0.04 in) dia. 99.95%	95	74
9	zinc powder 6 ~ 9 micro 97.5%	83	61
10	zinc, metal (powder)	95	84
11	zinc 99+% mossy	100	79
12	zinc foil, 0.38 mm	90	69

[a] Conversion and hydrolysis yield were determined by GC using trifluorotoluene as internal standard
[b] Yield of ethyl 4-iodobutanoate was determined by GC after iodometric titration

Table S1: Optimazation of C(Sp^2) organozincs under mechanochemical condition

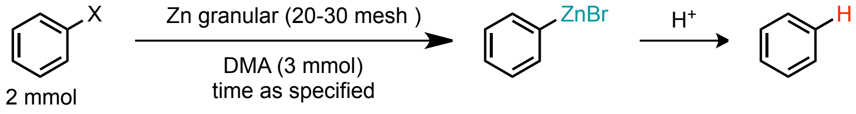

A B C

Entry	X	Zn [equivs]	Time [h]	Conv. [wrt% A][a]	Hydrolysis Yield [%C][a]
1	Cl	1.1	3	7	0
2	Br	1.1	3	29	19
3	I	1.1	3	63	50
4	I	1.5	4	82	66
5	I	2.5	4	95	80

[a] Conversion and hydrolysis yield were determined by GC using trifluorotoluene as internal standard

Table S2: Scope of mechanochemically generated C(Sp^3) organozinc reagents

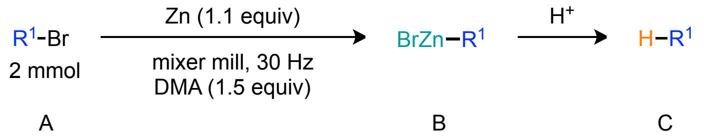

Entry	Substrate	Time [h]	Conv. [wrt% A][a]	Hydrolysis Yield [%C][a]
1	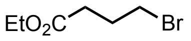	3 h<br>4 h	76<br>100	60<br>76 (52)[b]
2	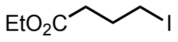	4 h	100	73
3	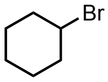	3 h	95	48
4	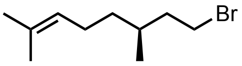	4 h	85	47
5	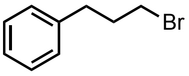	3 h<br>4 h	86<br>95	76<br>82
6	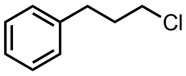	4 h	3	0
7	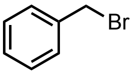	4 h	100	41
8	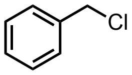	4 h	2	0
9	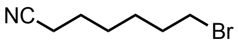	3 h	93	64
10	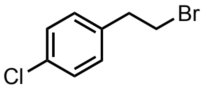	4h	90	87

Reaction conditions: alkyl halide (2 mmol), 20-30 mesh Zn granular (2.2 mmol), DMA (3 mmol), mixer mill, 30 Hz, time as specified
[a] Conversion and hydrolysis yield were determined by GC using trifluorotoluene as internal standard
[b] Yield of ethyl 4-iodobutanoate was determined by GC after iodometric titration

Table S3: Scope of mechanochemically generated C(Sp^2) organozinc reagents

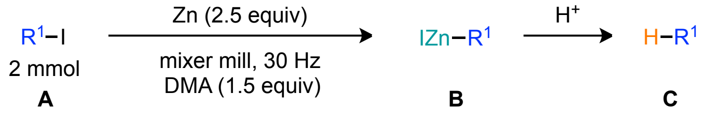

Entry	Substrate	Time [h]	Conv. [wrt% A][a]	Hydrolysis Yield [%C][a]
1	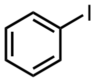	4 h	100	84
2	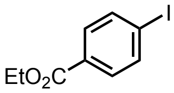	4 h<br>6 h	87<br>95	63<br>69
3	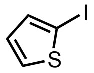	4 h	100	83
4	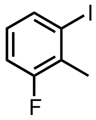	4 h	91	61
5	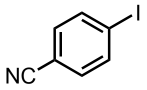	4 h	80	48
6	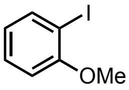	4 h	93	92
7	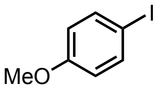	4 h	94	90
8	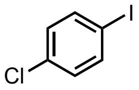	4 h	100	58
9	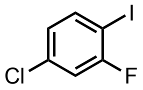	4 h	100	82

Reaction conditions: aryl iodide (2 mmol), 20-30 mesh Zn granular (5 mmol), DMA (3 mmol), mixer mill, 30 Hz, time as specified
[a] Conversion and hydrolysis yield were determined by GC using trifluorotoluene as internal standard

4 Characterization Data for Ligands and Pd(II) Complexes

1,4-bis(2,6-diisopropylphenyl)diazabuadiene (IPr)

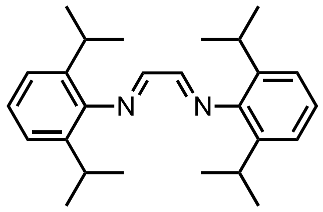

The title compound was synthesized using a method adapted from the literature.[2] To a 50 mL round-bottom flask, a solution of glyoxal (4.2 mmol, 0.603 g, 40 wt% in water), n-propanol (7 mL), 2,6-diisopropylaniline (1.62 g, 9.2 mmol) and H_2O (2 mL) were added. Then the reaction mixture was heated at 70 °C for 2 h. When the reaction was finished, 10 mL H_2O was added resulting in a bright yellow precipitate. Then the solid was filtered, washed with methanol (20 mL) and dried under *vacuo*. Yield: 0.312 g, 20%. **^{1}H NMR** (500 MHz, $CDCl_3$) δ 8.13 (s, 2H), 7.26 - 7.12 (m, 6H), 2.97 (hept, *J* = 6.9 Hz, 4H), 1.24 (d, *J* = 6.9 Hz, 24H) **^{13}C NMR** (126 MHz, $CDCl_3$) δ 163.2, 148.2, 136.9, 126.3, 123.3, 28.2, 23.6. NMR data is consistent with literature values.[2] HRMS (FTMS+) calcd for $[M+H]^+$ $C_{26}H_{37}N_2$: 377.2951, found: 377.2953.

IPr imidazolium chloride

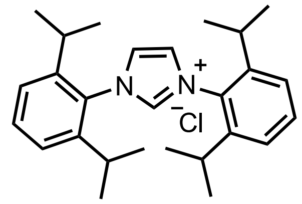

The title compound was prepared using a method modified from the literature.[3] **IPr** (0.753 g, 2 mmol) and methoxy(methyl)chloride (3.220 g, 40 mmol) were added to a glass vial. The vessel was sealed and stirred at 40 °C for 16 hours. When the reaction was finished, the mixture was cooled to room temperature and subsequent addition of diethylether (Et_2O, 15 mL) resulted a bright yellow precipitate. The bright yellow solid was then filtered off and washed with 50 mL Et_2O and dried in *vacuo*. Yield: 0.440 g, 52%. **^{1}H NMR** (500 MHz, $CDCl_3$) δ 9.58 (s, 1H), 8.07 (s, 2H), 7.57 (t, *J* = 7.8 Hz, 2H), 7.35 (dd, *J* = 7.9, 1.3 Hz, 4H), 2.43 (hept, *J* = 6.9 Hz, 4H), 1.28 (d, *J* = 6.8 Hz, 12 H), 1.22 (d, *J* = 6.9 Hz, 12H) **^{13}C NMR** (126 MHz, $CDCl_3$) δ 145.1, 138.1, 132.4, 129.9, 127.0, 124.9, 29.3, 24.9, 24.0. NMR data is consistent with literature values.[2] HRMS (ES+) calcd for $[M-Cl]^+$ $C_{27}H_{37}N_2$: 389.2957, found: 389.2961.

Pd-PEPPSI-IPr (6)

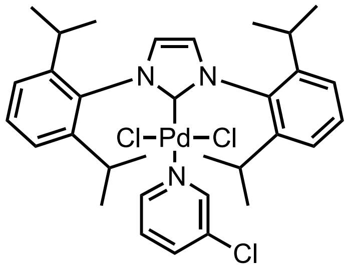

The title compound was prepared using a method modified from the literature.[4] To a thick wall glass vial $PdCl_2$ (0.088 g, 0.5 mmol), IPr imidazolium chloride (0.254 g, 0.6 mmol), K_2CO_3 (0.345 g, 2.5 mmol), 3-chloropyridine (2.5 mL) and a stirrer bar were added. Then the vial was sealed and the reaction mixture was then heated at 90 °C for 24 h. After cooling to room temperature, the mixture was diluted with 15 mL DCM and passed through a short pad of silica and washed with DCM (40 mL). The DCM in the solution was evaporated using rotary evaporator and 3-chloropyridine was distilled for reuse. Then the solid material was dissolved using a minimum amount of DCM and precipitated in hexane. The resulting yellow powder was filtered and dried under *vacuo*. Yield: 0.160 g, 47%. **^{1}H NMR** (500 MHz, $CDCl_3$) δ 8.62 (d, *J* = 2.4 Hz, 1H), 8.55 (dd, *J* = 5.5 Hz, 1.4 Hz, 1H), 7.57 (ddd, *J* = 8.2, 2.4, 1.4 Hz, 1H), 7.52 (t, *J* = 7.7 Hz, 2H), 7.38 (d, *J* = 7.7 Hz, 4H), 7.16 (s, *J* = 7.7 Hz, 2H), 7.09 (dd, *J* = 8.3, 5.5 Hz, 1H), 3.18 (hept, *J* = 6.8 Hz, 4H), 1.50 (d, *J* = 6.6 Hz, 12 H), 1.15 (d, *J* = 6.9 Hz, 12H). **^{13}C NMR** (126 MHz, $CDCl_3$) δ 153.6, 150.6, 149.6, 146.8, 137.6, 135.1, 132.1, 130.5, 125.3, 124.5, 124.2, 28.9, 26.5, 23.4. NMR data is consistent with literature values.[4] HRMS (ASAP+) calcd for $[M-Cl]^+$ $C_{32}H_{40}N_3Cl_2Pd$: 642.1634, found: 642.1638.

2,6-dibenzhydryl-4-methoxyaniline

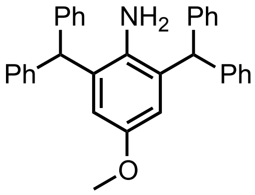

The title compound was synthesized using a method modified from the literature.[5] A 250 mL round bottom flask was charged with *p*-anisidine (2.980 g, 240 mmol) and diphenylmethanol (8.920 g, 480 mmol) and heated to 160 °C open to air. After the reaction mixture became homogeneous, a premade solution of anhydrous zinc chloride (1.62 g, 120 mmol) in concentrated HCl acid (37% in H_2O, 2 mL) was added dropwise. After 30 min, the reaction mixture became solid, the mixture was then cooled to room temperature and dissolved in 50 mL DCM. The DCM solution was washed with water (50 mL) multiple times, then dried over anhydrous $MgSO_4$. The solvent was evaporated, and the purple solid was crystalized using methanol to form white crystal. Yield: 7.42 g, 68%. **^{1}H NMR** (500 MHz, $CDCl_3$) δ 7.36 – 7.20 (m, 12 H), 7.17 – 7.06 (m, 8H), 6.22 (s, 2H), 5.51 (s, 2H), 3.45 (s, 3H), 3.16 (br s, 2H) **^{13}C NMR** (126 MHz, $CDCl_3$) δ 152.0, 142.6, 136.0, 131.0, 129.6, 128.7, 126.8, 114.5, 55.3, 52.6. NMR data is consistent with literature values.[5] HRMS (AP+) calcd for $[M+H]^+$ $C_{33}H_{30}NO$: 456.2327, found: 456.2327.

***N,N'*-Bis(2,6-bis(diphenylmethyl)-*p*-anisidyl)diazabutadiene (IPr*$^{(OMe)}$)**

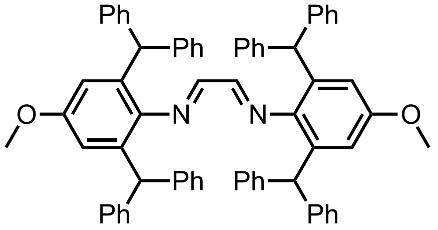

The title compound was synthesized using a method modified from the literature.[5] A solution of 2,6-dibenzhydryl-4-methoxyaniline (4.55 g, 10 mmol) in DCM (100 mL) was treated with anhydrous $MgSO_4$ (2.450 g, 20 mmol) followed by an aqueous solution of glyoxal (0.740 g, 5.1 mmol, 40 wt% in H_2O) and formic acid (0.05 mL, 1.3 mmol). The reaction was stirred at room temperature for 4 days before filtering and concentrating under vacuum to afford a brown solid. The crude solid was then recrystallized from boiling toluene to provide a pure diimine as bright yellow solid. Yield: 3.385 g, 73%. **^{1}H NMR** (500 MHz, $CDCl_3$) δ 7.24 - 7.12 (m, 26 H), 7.03 - 6.94 (m, 16H), 6.42 (s, 4H), 5.26 (s, 4H), 3.51 (s, 6H). **^{13}C NMR** (126 MHz, $CDCl_3$) δ 164.3, 156.3, 143.7, 143.0, 133.8, 129.6, 128.4, 126.6, 114.3, 55.2, 51.4. NMR data is consistent with literature values.[5] HRMS (FTMS+) calcd for $[M+H]^+$ $C_{68}H_{57}N_2O_2$: 933.4415, found: 933.4410.

IPr*(OMe) imidazolium chloride

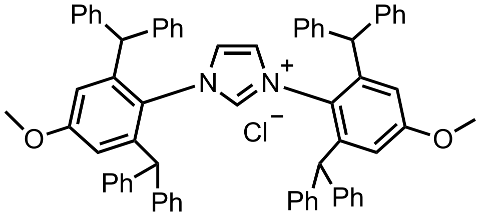

The title compound was prepared using a method modified from the literature.[3] **IPr*OMe** (1.745 g, 2 mmol) and ethoxy(methyl)chloride (1.860 g, 20 mmol) were added to a glass vial. The vessel was sealed and stirred at 100 °C for 16 hours. Once the reaction is finished, the mixture was cooled to room temperature and subsequent addition of diethylether (Et_2O, 15 mL) resulted a white precipitate. The white solid was then filtered off and washed with 50 mL Et_2O and dried in *vacuo*. Yield: 1.191 g, 61%. **^{1}H NMR** (500 MHz, $CDCl_3$) δ 12.88 (s, 1H), 7.32 - 7.24 (m, 18H), 7.23 - 7.11(m, 16H), 6.68 - 6.80 (m, 8H), 6.49 (s, 4H), 5.44 (s, 2H), 5.32 (s, 4H), 3.54 (s, 6H) **^{13}C NMR** (126 MHz, $CDCl_3$) δ 160.7, 142.6, 142.3, 141.6, 129.3, 129.0, 128.6, 128.5, 126.9, 126.8, 125.2, 123.3, 115.5, 55.1, 51.4. NMR data is consistent with literature values.[5] HRMS (EI+) calcd for $[M-Cl]^+$ $C_{69}H_{57}N_2O_2$: 945.4420, found: 945.4421.

Pd-PEPPSI-IPr*(OMe) (8)

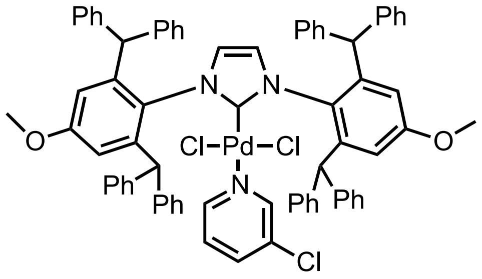

The title compound was prepared using a method modified from the literature.[6] To a thick wall glass vial $PdCl_2$ (0.088 g, 0.5 mmol), IPr*(OMe) imidazole chloride (0.539 g, 0.55 mmol), K_2CO_3 (0.345 g, 2.5 mmol), 3-chloropyridine (2.5 mL) and a stir bar were added. The vial was sealed and the reaction mixture was heated at 100 °C for 24 h. After cooling to room temperature, the mixture was diluted with 15 mL DCM and passed through a short pad of silica and washed with DCM (40 mL). The DCM in the solution was evaporated using rotary evaporator and 3-chloropyridine was distilled for reuse. The solid material was dissolved using a minimum amount of DCM and precipitated with hexane. The resulting off white powder was filtered and dried under *vacuo*. Yield: 0.127 g, 21%. **^{1}H NMR** (500 MHz, $CDCl_3$) δ 9.20 (d, *J* = 2.1 Hz, 1H), 9.07 (dd, *J* = 5.5, 1.3 Hz, 1H), 7.82 (ddd, *J* = 8.2, 2.4, 1.3 Hz, 1H), 7.45 (d, *J* = 7.3 Hz, 8H), 7.35 (ddd, *J* = 8.2, 5.5, 0.6 Hz, 1H), 7.26 – 7.18 (m, 12 H), 7.10 - 7.02 (m, 12H), 6.80 - 6.76 (m, 2H), 6.50 (s, 4H), 6.34 (s, 4H), 4.85 (s, 2H), 3.53 (s, 6H). **^{13}C NMR** (126

MHz, $CDCl_3$) δ 159.0, 151.0, 150.3, 149.9, 144.4, 143.9, 143.8, 138.1, 132.7, 130.9, 130.6, 129.5, 128.3, 128.0, 126.4, 126.3, 124.8, 124.3, 115.6, 55.0, 51.2. HRMS (ES+) calcd for $[M-Cl-pyCl]^+$ $C_{69}H_{56}N_2O_2ClPd$: 1085.3065, found: 1085.3087. **IR** ν 1597, 1493, 1464, 1094, 698 cm^{-1}

1,2-bis[(2,6-diisopropylphenyl)imino]acenaphthene (BIAN)

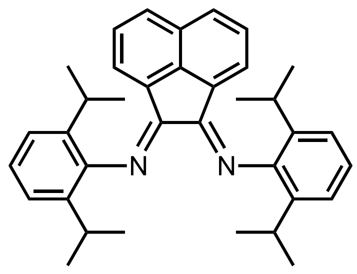

The title compound was prepared using a method modified from the literature.[7] In a 500 mL round bottom flask, acenaphthenequinone (3.500 g, 19.2 mmol) was suspended in acetonitrile (200 mL) and heated under reflux for 60 min. Then acetic acid (80 mL) was added and the mixture was refluxed for another 60 min. Following this 2,6-diisopropylphenylaniline (8.000 g, 45 mmol) was added dropwise to the hot solution. The mixture was further heated under reflux for another 5 hours. Once the reaction was finished, the reaction was cooled to room temperature. The resulting orange-yellow solid was filtered and washed with hexane (40 mL) three times, and dried under vacuum. Yield: 7.698 g, 80%. **^{1}H NMR** (500 MHz, $CDCl_3$) δ 7.90 (d, J = 8.3 Hz, 2H), 7.38 (t, J = 7.7 Hz, 2H), 7.34 - 7.21 (m, 6H), 6.66 (d, J = 7.2 Hz, 2H), 3.14 - 2.91 (m, 4H), 2.16 (d, J = 6.8 Hz, 12H), 0.99 (d, J = 6.9 Hz, 12H) **^{13}C NMR** (126 MHz, $CDCl_3$) δ 161.1, 147.6, 140.9, 135.6, 131.3, 129.7, 129.0, 128.0, 124.4, 123.6, 123.5, 28.8, 23.6, 23.3. NMR data is consistent with literature values.[8] HRMS (FTMS+) calcd for $[M+H]^+$ $C_{36}H_{41}N_2$: 501.3270, found: 501.3253.

IPr(BIAN) imidazolium chloride

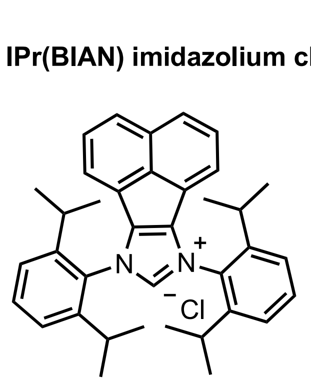

The title compound was prepared using a method modified from the literature.[3] BIAN (1.000 g, 2 mmol) and methoxy(methyl)chloride (3.220 g, 40 mmol) were added to a glass vial. The vessel was sealed and stirred at 100 °C for 16 hours. Once the reaction was finished, the mixture was cooled to room temperature and whereupon addition of diethylether (Et_2O 15 mL)

resulted a yellow precipitate. Then the yellow solid was filtered off and washed with 50 mL Et_2O and dried in *vacuo*. Yield: 0.850 g, 78%. **^{1}H NMR** (500 MHz, $CDCl_3$) δ 12.2 (s, 1H), 7.99 (d, *J* = 8.3 Hz, 2H), 7.66 (t, *J* = 7.9 Hz, 2H), 7.57 (dd, *J* = 8.3, 7.1 Hz), 7.46 (d, *J* = 7.9 Hz, 4H), 7.22 (d, *J* = 7.1 Hz, 2H), 2.73 (hept, *J* = 6.8 Hz, 4H), 1.96 (s, 4H), 1.40 (d, *J* = 6.8 Hz, 12H), 1.16 (d, *J* = 6.8 Hz, 12 H). **^{13}C NMR** (126 MHz, $CDCl_3$) δ 145.1, 144.6, 137.6, 132.3, 130.8, 130.4, 130.2, 129.5, 128.4, 125.1, 123.6, 122.9, 29.63, 24.9, 23.7. NMR data is consistent with literature values.[3] HRMS (FTMS+) calcd for $[M-Cl]^+$ $C_{37}H_{41}N_2$: 513.3264, found: 513.3254.

Pd-PEPPSI-IPr$^{(BIAN)}$ (9)

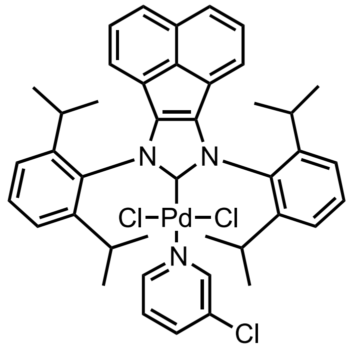

The title compound was prepared using a method modified from the literature.[6] To a thick wall glass vial $PdCl_2$ (0.355 g, 2 mmol), IPr(BIAN) imidazolium chloride (1.316g, 2.4 mmol), K_2CO_3 (2.760 g, 20 mmol), 3-chloropyridine (5 mL) and a stirrer bar were added. Then the vial was sealed and the reaction mixture was heated at 90 °C for 24 h. After cooling to room temperature, the mixture was diluted with 15 mL DCM and passed through a short pad of silica and washed with DCM (40 mL). The DCM in the solution was evaporated using rotary evaporator and 3-chloropyridine was distilled for reuse. Then the solid material was dissolved using a minimum amount of DCM and precipitated with hexane. The resulting yellow powder was filtered and dried under *vacuo*. Yield: 0.917 g, 58%. **^{1}H NMR** (500 MHz, $CDCl_3$) δ 8.68 (d, *J* = 2.4 Hz, 1H), 8.61 (dd, *J* = 5.5, 1.4 Hz, 1H), 7.70 (d, *J* = 8.2 Hz, 2H), 7.64 (t, *J* = 7.8 Hz, 2H), 7.57 (ddd, *J* = 8.2, 2.4, 1.4Hz, 2H), 7.48 (d, *J* = 7.8 Hz, 4H), 7.34 (dd, *J* = 8.3, 7.0 Hz, 2H), 7.10 (dd, *J* = 8.2, 5.5 Hz, 1H), 6.8 (d, *J* = 7.0 Hz, 2H), 3.40 (hept, *J* = 6.7 Hz, 4H), 1.46 (d, *J* = 6.6Hz, 12 H), 0.92 (d, *J* = 6.9 Hz, 12H) **^{13}C NMR** (126 MHz, $CDCl_3$) δ 159.1, 150.6, 149.6, 147.3, 140.4, 137.4, 133.8, 131.9, 130.7, 129.6, 129.1, 128.1, 127.3, 126.1, 124.8, 124.3, 122.2, 28.9, 25.8, 24.3. NMR data is consistent with literature values.[6] HRMS (FTMS+) calcd for $[M-Cl]^+$ $C_{42}H_{44}N_3Pd$: 766.1951, found: 766.1936.

5 Product Characterization data

Ethyl 4-phenylbutanoate (5)

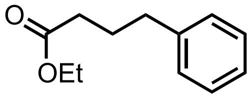

Prepared according to General Method 1. Purified by column chromatography (Ethyl Acetate/Petroleum ether = 20%) to give the product (0.142 g, 74%) as a colourless oil. **^{1}H NMR** (500 MHz, $CDCl_3$) δ 7.33 - 7.26 (m, 2H), 7.26 - 7.17 (m, 3H), 4.16 (q, J = 7.1 Hz, 2H), 2.68 (t, J = 7.5 Hz, 2H), 2.35 (t, J = 7.5 Hz, 2H), 2.05 - 1.90 (m, 2H), 1.28 (t, J = 7.1 Hz, 3H). **^{13}C NMR** (126 MHz, $CDCl_3$) δ 173.5, 141.5, 128.5, 128.4, 126.0, 60.3, 35.2, 33.7, 26.6, 14.3. NMR data is consistent with literature values.[9] **HRMS** (FTMS+) calc. for $[M+H]^+$ $C_{12}H_{17}O_2$: 193.1228 found: 193.1222.

Note: The title compound could also be prepared using a one-pot one-step method with different zinc forms (General Method 2). **20-30 mesh zinc granular:** Yield = 80%, 0.154 g; **zinc powder:** Yield = 78%, 0.150 g; **zinc puriss** Yield = 79%, 0.151 g.

4-(3-phenylpropyl)benzonitrile (10)

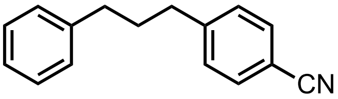

Prepared according to General Method 1. Purified by column chromatography (Ethyl Acetate/Petroleum ether = 10%) to give the product (0.211 g, 94%) as a yellow oil. **^{1}H NMR** (500 MHz, $CDCl_3$) δ 7.52 (d, J = 8.4 Hz, 2H), 7.28 - 7.20 (m, 4H), 7.13 (m, 3H), 2.69 - 2.57 (m, 4H), 1.97 - 1.87 (m, 2H). **^{13}C NMR** (126 MHz, $CDCl_3$) δ 148.1, 141.7, 132.3, 129.4, 128.6, 128.5, 126.1, 119.3, 109.8, 35.6, 35.4, 32.6. NMR data is consistent with literature values.[10] **HRMS** (ASAP+) calcd for $[M+H]^+$ $C_{16}H_{16}N$: 222.1283, found: 222.1281.

Note: The title compound could also be prepared using a one-pot one-step method with different zinc forms (General Method 2). **20-30 mesh zinc granular:** Yield = 76%, 0.168 g; **zinc powder:** Yield = 74%, 0.164 g; **zinc puriss** Yield = 84%, 0.186 g.

Ethyl 4-(3-phenylpropyl)benzoate (11)

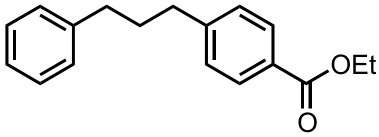

Prepared according to General Method 1. Purified by column chromatography (Ethyl Acetate/Petroleum ether = 10%) to give the product (0.239 g, 89%) as colourless oil. **^{1}H NMR**

(500 MHz, $CDCl_3$) δ 7.99 (d, *J* = 8.3, 2H), 7.30 (ddd, *J* = 18.4, 8.6, 7.1 Hz, 4H), 7.24 - 7.18 (m, 3H), 4.40 (q, *J* = 7.1 Hz, 2H), 2.76 - 2.64 (m, 4H), 2.01 (ddt, *J* = 13.6, 9.2, 6.9 Hz, 2H), 1.42 (t, *J* = 7.1 Hz, 3H). **^{13}C NMR** (126 MHz, $CDCl_3$) δ 166.8, 147.8, 142.1, 129.8, 128.6, 128.5, 128.3, 126.0, 60.9, 35.6, 35.5, 32.8, 14.5. NMR data is consistent with literature values.[10] **HRMS** (FTMS+) calcd for $[M+H]^+$ $C_{18}H_{20}O_2$: 269.1536, found: 269.1538.

Note: The title compound could also be prepared using a one-pot one-step method with different zinc forms (General Method 2). **20-30 mesh zinc granular:** Yield = 85%, 0.228 g; **zinc powder:** Yield = 96%, 0.257 g; **zinc puriss** Yield = 99%, 0.267 g.

2-(3-phenylpropyl)phenyl 4-methylbenzenesulfonate (12)

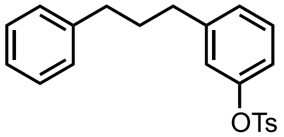

Prepared according to General Method 1. Purified by column chromatography (Ethyl Acetate/Petroleum ether = 20%) to give the product (0.353 g, 95%) as a colourless sticky oil. **^{1}H NMR** (500 MHz, $CDCl_3$) δ 7.74 - 7.70 (m, 2H), 7.34 - 7.27 (m, 4H), 7.25 - 7.15 (m, 4H), 7.09 - 7.05 (m, 1H), 6.87 - 6.83 (m, 1H), 6.82 - 6.79 (m, 1H), 2.58 (dt, *J* = 10.9, 7.7 Hz, 4H), 2.40 (s, 3H), 1.95 - 1.77 (m, 2H). **^{13}C NMR** (126 MHz, $CDCl_3$) δ 149.8, 145.4, 144.4, 142.0, 132.6, 129.8, 129.5, 128.7, 128.5, 128.5, 127.4, 126.0, 122.5, 119.9, 35.3, 35.1, 21.8. **HRMS** (ASAP+) calcd for $[M+H]^+$ $C_{22}H_{23}O_3S$: 367.1368, found: 367.1369. **IR** ν 2361, 1599, 1371, 1177, 550, 365 cm^{-1}.

2-fluoro-4-(3-phenylpropyl)benzonitrile (13)

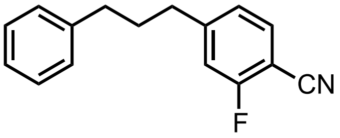

Prepared according to General Method 1. Purified by column chromatography (Ethyl Acetate/Petroleum ether = 10%) to give the product (0.218 g, 88%) as a yellow oil. **^{1}H NMR** (500 MHz, $CDCl_3$) δ 7.55 – 7.49 (m, 1H), 7.33 – 7.27 (m, 2H), 7.24 - 7.14 (m, 3H), 7.09 – 7.00 (m, 2H), 2.73 - 2.63 (m, 4H), 1.97 (dq, *J* = 15.4, 7.7, 2H). **^{13}C NMR** (126 MHz, $CDCl_3$) δ 163.4 (d, *J* = 259 Hz), 151.4 (d, *J* = 8 Hz), 141.4, 133.4, 128. 6, 128.5, 126.2, 125.1 (d, *J* = 3 Hz), 116.4 (d, *J* = 19 Hz), 114.4, 98.9 (d, *J* = 16 Hz), 35.5 (d, *J* = 2 Hz), 35.3, 32.2. **^{19}F NMR** (376 MHz, $CDCl_3$) δ -107.0. **HRMS** (FTMS) calcd for $[M+NH_4]^+$ $C_{16}H_{14}FN$: 257.1449, found: 257.1451. **IR** ν 2932, 2230, 1620, 1566, 1497, 1427.32, 1250, 1111, 826, 748, 694 cm^{-1}.

4,4,5,5-tetramethyl-2-(4-(3-phenylpropyl)phenyl)-1,3,2-dioxaborolane (14)

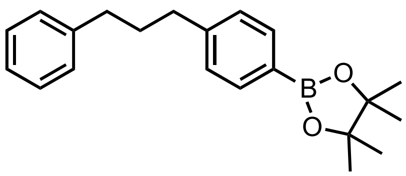

Prepared according to General Method 1. Purified by column chromatography (Ethyl Acetate/Petroleum ether = 10%) to give the product (0.202 g, 63%) as a yellow oil. **^{1}H NMR** (500 MHz, $CDCl_3$) δ 7.75 (d, *J* = 7.9, 2H), 7.31 - 7.27 (m, 2H), 7.20 (dd, *J* = 13.3, 7.8, 5H), 2.70 - 2.62 (m, 4H), 1.97 (dq, *J* = 15.4, 7.7, 2H), 1.35 (s, 12H). **^{13}C NMR** (126 MHz, $CDCl_3$) δ 145.9, 142.4, 135.0, 128.6, 128.4, 128.1, 125.9, 83.8, 35.8, 35.5, 32.9, 25.0. NMR data is consistent with literature values.[11] **HRMS** (ES+) calcd for $[M+H]^+$ $C_{13}H_9N$: 323.2182, found: 323.2186. **IR** v 2976, 2930, 1611, 1398, 1360, 1319, 1271, 1144, 1090, 737, 700, 660 cm^{-1}.

4-cyclohexyl-2-fluorobenzonitrile (15)

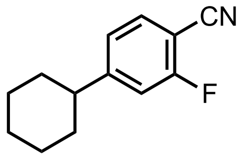

Prepared according to General Method 1. Purified by column chromatography (Ethyl Acetate/Petroleum ether = 5%) to give the product (0.095 g, 46%) as a yellow oil. **^{1}H NMR** (500 MHz, $CDCl_3$) δ 7.52 (dd, *J* = 7.9, 6.8 Hz, 1H), 7.07 (ddd, *J* = 11.7, 9.2, 1.3 Hz, 2H), 2.56 (dd, *J* = 10.0, 7.5 Hz, 1H), 1.86 (d, *J* = 9.4 Hz, 4H), 1.80 - 1.72 (m, 1H), 1.46 - 1.32 (m, 4H), 1.31 - 1.19 (m, 1H). **^{13}C NMR** (126 MHz, $CDCl_3$) δ 163.5 (d, *J* = 258 Hz), 157.0 (d, *J* = 7 Hz), 133.3, 123.7 (d, *J* = 3 Hz), 114.8 (d, *J* = 19 Hz), 114.5, 98.7 (d, *J* = 16 Hz), 44.8 (d, *J* = 1.4 Hz), 33.9, 26.6, 25.9. **^{19}F NMR** (376 MHz, $CDCl_3$) δ -107.0. **HRMS** (FTMS+) calcd for $[M+NH_4]^+$ $C_{13}H_{18}N_2F$: 221.1449, found: 221.1449. **IR** v 2926, 2853, 2236, 1618, 1566, 1497, 1449, 1429, 1250, 1111, 949, 824 cm^{-1}.

(S)-1-(4-[3,7-dimethyloct-6-en-1-yl)phenyl]ethan-1-one) (16)

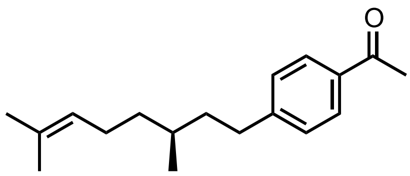

Prepared according to General Method 1. Purified by column chromatography (Ethyl Acetate/Petroleum ether = 5%) to give the product (0.138 g, 54%) as a yellow oil. **^{1}H NMR** (500 MHz, $CDCl_3$) δ 7.88 (d, *J* = 8.2 Hz, 2H), 7.27 (d, *J* = 6.2 Hz, 2H), 5.12 - 5.07 (m, 1H), 2.71 (ddd, *J* = 15.5, 10.5, 5.3 Hz, 1H), 2.66 - 2.60 (m, 1H), 2.59 (s, 3H), 2.07 - 1.90 (m, 2H), 1.69 (s, 3H), 1.66 - 1.63 (m, 1H), 1.60 (s, 3H), 1.52 - 1.34 (m, 3H), 1.24 - 1.15 (m, 1H), 0.95 (d, *J* = 6.3 Hz, 3H). **^{13}C NMR** (126 MHz, $CDCl_3$) δ 198.0, 149.2, 135.0, 131.4, 128.7, 128.6, 124.9, 38.6, 37.0, 33.7, 32.3, 26.7, 25.9, 25.6, 19.6, 17.8. **HRMS** (FTMS) calcd for $[M+H]^+$

$C_{18}H_{26}O$: 257.1449, found: 257.1451. **IR** ν 2959, 2911, 2853, 1682, 1605, 1570, 1412, 1375, 1356, 1265, 1180, 1016, 955, 818 cm^{-1}.

4-cyclohexylbenzonitrile (17)

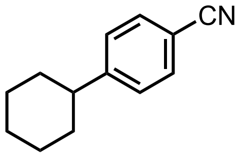

Prepared according to General Method 1. Purified by column chromatography (Ethyl Acetate/Petroleum ether = 10%) to give the product (0.133 g, 72%) as a yellow oil. **^{1}H NMR** (500 MHz, $CDCl_3$) δ 7.57 (d, *J* = 8.3 Hz, 2H), 7.30 (d, *J* = 8.1 Hz, 2H), 2.61 - 2.50 (m, 1H), 1.86 (d, *J* = 8.9 Hz, 4H), 1.80 - 1.72 (m, 1H), 1.40 (dd, *J* = 13.1, 7.1 Hz, 4H), 1.32 - 1.20 (m, 1H). **^{13}C NMR** (126 MHz, $CDCl_3$) δ 153.6, 132.3, 127.8, 119.4, 109.7, 44.9, 34.1, 26.7, 26.0. NMR data is consistent with literature values.[12] **HRMS** (FTMS) calcd for $[M+NH_4]^+$ $C_{13}H_{19}N_2$: 203.1543, found: 203.1543.

4-(4-chlorophenethyl)-3-fluorobenzonitrile (18)

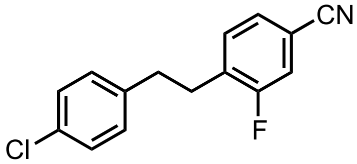

Prepared according to General Method 1. Purified by column chromatography (Ethyl acetate/Petroleum = 0 - 10%) to yield the product as a white solid (0.201 g, 0.77 mmol, 77%). **^{1}H NMR** (500 MHz, $CDCl_3$) δ 7.41 (t, *J* = 7.3 Hz, 1H), 7.15 (d, *J* = 8.3 Hz, 2H), 7.04 - 6.86 (m, 4H), 2.94 - 2.73 (m, 4H). **^{13}C NMR** (126 MHz, $CDCl_3$) δ 163.2 (d, *J* = 259 Hz), 150.1 (d, *J* = 8 Hz), 138.6, 133.4, 132.3, 129.8, 128.7, 125.2 (d, *J* = 3 Hz), 116.5 (d, *J* = 19 Hz), 114.2, 99.1 (d, *J* = 16 Hz), 37.7 (d, *J* = 2 Hz), 36.2. **^{19}F NMR** (471 MHz, $CDCl_3$) δ -106.82 (dd, *J* = 9.7, 6.2 Hz). **HRMS** (EI+) calc. for $C_{15}H_{11}NFCl$: 259.0564, found 259.0560. **IR**: 2936, 2234, 1620, 1566, 1493, 1431, 1288, 1250, 1111, 1092, 1015, 953, 891, 826, 748, 660, 617, 536, 501 cm^{-1}. Melting point: (ethyl acetate) 82 - 83 °C.

ethyl 4-(6-cyanohexyl)benzoate (19)

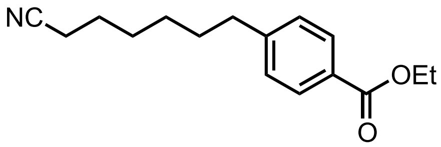

Prepared according to General Method 1. Purified by column chromatography (Ethyl Acetate/Petroleum ether = 20%) to give the product (0.215 g, 82%) as colourless oil. **^{1}H NMR** (500 MHz, $CDCl_3$) δ 7.99 - 7.82 (m, 2H), 7.23 - 7.06 (m, 2H), 4.29 (q, *J* = 7.1 Hz, 2H), 2.60 (t, *J* = 7.7 Hz, 2H), 2.25 (t, *J* = 7.1 Hz, 2H), 1.63 - 1.51 (m, 4H), 1.47 - 1.37 (m, 2H), 1.33 - 1.20 (m, 5H). **^{13}C NMR** (126 MHz, $CDCl_3$) δ 166.8, 147.9, 129.8, 128.5, 128.3, 119.8, 60.9, 35.9, 30.9, 28.6, 28.4, 25.4, 17.2, 14.5. **IR** v 2932, 2859, 1711, 1611, 1271, 1103 cm^{-1}

1-(4-(4-chlorophenethyl)phenyl)ethanone (20)

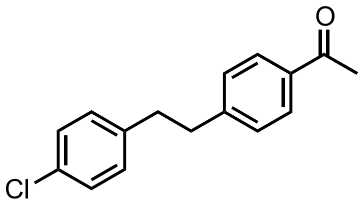

Prepared according to General Method 1. Purified by column chromatography (Ethyl acetate/Petroleum ether = 0 - 10%) to yield the product as a white solid (0.122 g, 47%). **^{1}H NMR** (400 MHz, $CDCl_3$) δ 7.79 (d, *J* = 8.3 Hz, 2H), 7.21 - 7.08 (m, 4H), 6.97 (d, *J* = 8.3 Hz, 2H), 2.96 - 2.77 (m, 4H), 2.50 (s, 3H). **^{13}C NMR** (101 MHz, $CDCl_3$) δ 197.9, 147.0, 139.5, 135.3, 131.9, 129.9, 128.8, 128.6, 128.5, 37.7, 36.7, 26.6. NMR data is consistent with literature values.[13] **IR** v 1674, 1601, 1489, 1406, 1360, 1263, 1090, 1013, 955, 828, 579, 517 cm^{-1}. **HRMS** (EI+) calc. for $C_{16}H_{15}OCl$: 258.0811, found: 258.0811. Melting point (chloroform): 104 -105 °C.

Ethyl 4-(3,5-dimethoxyphenyl)butanoate (21)

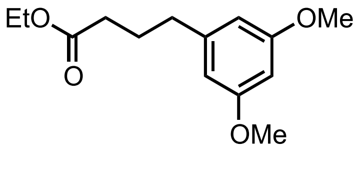

Prepared according to General Method 1. Purified by column chromatography (Ethyl Acetate/Petroleum ether = 5-10%) and Kugelrhor distillation (200 °C. 4 mbar) to give the product (0.076 g, 33%) as a colourless oil. **^{1}H NMR** (500 MHz, $CDCl_3$) δ 6.34 (d, *J* = 2.3 Hz, 2H), 6.31 (t, *J* = 2.3 Hz, 1H), 4.13 (q, *J* = 7.1 Hz, 2H), 3.78 (s, 6H), 2.62 - 2.57 (m, 2H), 2.32 (t, *J* = 7.5 Hz, 2H), 2.00 - 1.89 (m, 2H), 1.25 (t, *J* = 7.1 Hz, 3H). **^{13}C NMR** (126 MHz, $CDCl_3$) δ 173.7, 160.9, 144.0, 106.7, 98.1, 60.4, 55.4, 35.6, 33.8, 26.5, 14.4. NMR data is consistent with literature values.[14] **HRMS** (ES+) calc. for $[M+Na]^+$ $C_{14}H_{20}O_4$: 275.1259, found: 275.1272. **IR** v 2940, 2839, 2361, 1728, 1597, 1458, 1427, 1204, 1150, 1057, 833 cm^{-1}.

2-(3-phenylpropyl)thiophene (22)

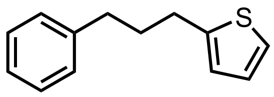

Prepared according to General Method 1. Purified by column chromatography (Ethyl Acetate/Petroleum ether = 5-10%) to give the product **(**0.085 g, 42%) as a colourless oil. **^{1}H NMR** (500 MHz, $CDCl_3$) δ 7.20 (dd, *J* = 8.4, 6.6 Hz, 2H), 7.13 - 7.07 (m, 3H), 7.02 (dd, *J* = 5.1, 1.2 Hz, 1H), 6.83 (dd, *J* = 5.1, 3.4 Hz, 1H), 6.72 - 6.68 (m, 1H), 2.77 (t, *J* = 7.4 Hz, 2H), 2.60 (t, *J* = 7.6 Hz, 2H), 1.93 (dt, *J* = 15.2, 7.6 Hz, 2H). **^{13}C NMR** (126 MHz, $CDCl_3$) δ 145.3, 142.1, 128.6, 128.5, 126.8, 126.0, 124.3, 123.1, 35.3, 33.4, 29.5. **HRMS** (EI+) calcd for $[M]^+$ $C_{13}H_{14}S$: 202.0816, found: 202.0813. **IR** ν 3024, 2928, 2855, 1493, 1454, 1238.30, 1076, 1030, 849, 822, 741, 691, 490 cm^{-1}.

Ethyl-4-benzylbenzoate (23)

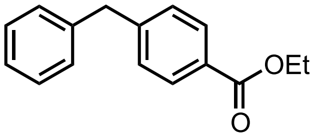

Prepared according to General Method 1. Purified by column chromatography (Ethyl Acetate/Petroleum ether = 5%) to give the product (0.132 g, 55%) as a colourless oil. **^{1}H NMR** (500 MHz, $CDCl_3$) δ 7.99 (d, *J* = 8.3 Hz, 2H), 7.36 - 7.22 (m, 5H), 7.22 - 7.17 (m, 2H), 4.39 (q, *J* = 7.1 Hz, 2H), 4.06 (s, 2H), 1.41 (t, *J* = 7.1Hz, 3H). **^{13}C NMR** (126 MHz, $CDCl_3$) δ 166.7, 146.5, 140.3, 129.9, 129.08, 129.1, 128.7, 128.6, 126.5, 61.0, 42.1, 14.5. NMR data is consistent with literature values.[15] **HRMS** (ES+) calcd for $[M+H]^+$ $C_{16}H_{16}O_2$: 241.1229, found: 241.1228.

Ethyl (S)-4-(3,7-dimethyloct-6-en-1-yl)benzoate (24)

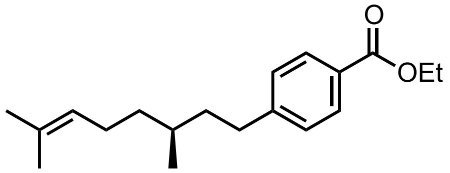

Prepared according to General Method 1. Initial purification by column chromatography (Ethyl Acetate/Petroleum ether = 0 - 10%). Further purified by Kugelrohr distillation (120 °C, 4 mbar) to yield the product as a colourless oil (0.148 g, 51%). **^{1}H NMR** (400 MHz, $CDCl_3$) δ 7.86 (d, *J* = 8.3 Hz, 2H), 7.13 (d, *J* = 8.3 Hz, 2H), 5.19 – 4.85 (m, 1H), 4.26 (q, *J* = 7.1 Hz, 2H), 2.75 - 2.44 (m, 2H), 1.97 - 1.80 (m, 2H), 1.59 (s, 3H), 1.56 – 1.52 (m, 1H), 1.50 (s, 3H), 1.38 - 1.32 (m, 3H), 1.28 (t, *J* = 7.1 Hz, 3H), 1.15 - 1.04 (m, 1H), 0.84 (d, *J* = 6.2 Hz, 3H). **^{13}C NMR** (101 MHz, $CDCl_3$) δ 166.7, 148.6, 131.2, 129.7, 128.4, 128.0, 124.8, 60.8, 38.6, 37.0, 33.6, 32.1, 25.8, 25.5, 19.5, 17.7, 14.4. **HRMS** (EI+) calc. for $C_{19}H_{28}O_2$: 288.2089, found: 288.2097. **IR** ν 2916, 1713, 1612, 1458, 1373, 1273, 1173, 1103, 1026, 849, 764, 702 cm^{-1}.

4-(4-chlorophenethyl)benzonitrile (25)

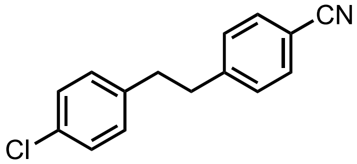

Prepared according to General Method 1. Purified by column chromatography (Ethyl Acetate/Petroleum ether = 5 - 10%) to give the product (0.223 g, 92%) as a white solid. **^{1}H NMR** (500 MHz, $CDCl_3$) δ 7.55 (d, *J* = 8.2 Hz, 2H), 7.22 (dd, *J* = 11.4, 8.3 Hz, 4H), 7.03 (d, *J* = 8.3 Hz, 2H), 3.00 - 2.93 (m, 2H), 2.92 - 2.86 (m, 2H). **^{13}C NMR** (126 MHz, $CDCl_3$) δ 146.9, 139.1, 132.4, 132.2, 129.9, 129.5, 128.7, 119.1, 110.2, 37.9, 36.7. **HRMS** (EI+) calcd for $[M]^+$ $C_{15}H_{12}ClN$: 241.0658, found: 241.0660. **IR** ν 2928, 2862, 2226, 1605, 1505, 1489, 1456, 1406, 1177, 1090, 1013, 828, 557, 519. Melting point (ethyl acetate): 107 - 111 °C.

[1,1'-biphenyl]-4-carbonitrile (26)

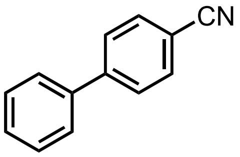

Prepared according to General Method 1. Purified by column chromatography (Ethyl Acetate/Petroleum ether = 20%) to give the product (0.125 g, 87%) as a white solid. **^{1}H NMR** (500 MHz, $CDCl_3$) δ 7.73 (d, *J* = 8.2 Hz, 2H), 7.69 (d, *J* = 8.4 Hz, 2H), 7.62 - 7.56 (m, 2H), 7.49 (dd, *J* = 8.1, 6.7 Hz, 2H), 7.45 - 7.40 (m, 1H). **^{13}C NMR** (126 MHz, $CDCl_3$) δ 145.8, 139.3, 132.7, 129.3, 128.8, 127.9, 127.4, 119.1, 111.1. NMR data is consistent with literature values.[16] **HRMS** (ASAP+) calcd for $[M+H]^+$ $C_{13}H_9N$: 180.0813, found: 180.0814. Melting point (ethyl acetate): 84 - 86 °C.

Ethyl [1,1'-biphenyl]-4-carboxylate (27)

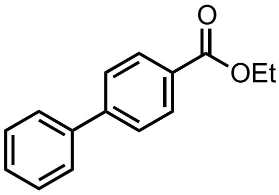

Prepared according to General Method 1. Purified by column chromatography (Ethyl Acetate/Petroleum ether = 5%) to give the product (0.216 g, 98%) as a white crystalline solid. **^{1}H NMR** (500 MHz, $CDCl_3$) δ 8.12 (d, *J* = 8.6 Hz, 2H), 7.69 - 7.60 (m, 4H), 7.47 (t, *J* = 7.5 Hz, 2H), 7.42 - 7.37 (m, 1H), 4.41 (q, *J* = 7.1 Hz, 2H), 1.42 (t, *J* = 7.1 Hz, 3H). **^{13}C NMR** (126 MHz, $CDCl_3$) 166.7, 145.7, 140.2, 130.2, 129.4, 129.1, 128.3, 127.4, 127.2, 61.1, 14.5. NMR data is consistent with literature values.[16] **HRMS** (ES+) calcd for $[M+H]^+$ $C_{15}H_{14}O_2$: 227.1072, found: 227.1070. Melting point (ethyl acetate): 49 - 51 °C.

(4'-methoxy-[1,1'-biphenyl]-4-yl)(methyl)sulfane (28)

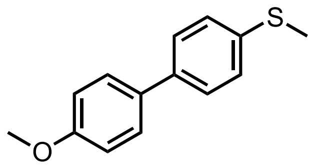

Prepared according to General Method 1. Purified by column chromatography (Ethyl Acetate/Petroleum ether = 10%) to give the product (0.094 g, 42%) as a white solid. **^{1}H NMR** (500 MHz, $CDCl_3$) δ 7.49 (dd, *J* = 11.6, 8.7 Hz, 4H), 7.31 (d, *J* = 8.6 Hz, 2H), 6.97 (d, *J* = 8.9 Hz, 2H), 3.85 (s, 3H), 2.52 (s, 3H). **^{13}C NMR** (126 MHz, $CDCl_3$) δ 159.2, 137.9, 136.8, 133.2, 128.0, 127.9, 127.2, 114.4, 55.5, 16.2. NMR data is consistent with literature values.[17] **HRMS** (ASAP+) calcd for $[M+H]^+$ $C_{14}H_{14}OS$: 231.0838, found: 231.0839. Melting point (ethyl acetate): 149 - 153 °C.

Ethyl 4'-cyano-3'-fluoro-[1,1'-biphenyl]-4-carboxylate (29)

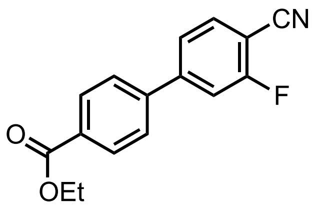

Prepared according to General Method 1. Purified by column chromatography (Ethyl Acetate/Petroleum ether = 10%) to give the product (0.156 g, 57%) as a white solid. **^{1}H NMR** (500 MHz, $CDCl_3$) δ 8.16 (d, *J* = 8.6 Hz, 2H), 7.72 (dd, *J* = 8.0, 6.7 Hz, 1H), 7.64 (d, *J* = 8.5 Hz, 2H), 7.51 (dd, *J* = 8.1, 1.6 Hz, 1H), 7.46 (dd, *J* = 10.0, 1.5 Hz, 1H), 4.42 (q, *J* = 7.1 Hz, 2H), 1.42 (t, *J* = 7.1 Hz, 3H). **^{13}C NMR** (126 MHz, $CDCl_3$) δ 166.1, 163.6 (d, *J* = 259 Hz), 147.5 (d, *J* = 8 Hz), 142.2 (d, *J* = 2 Hz), 134.1, 131.3, 130.6, 127.3, 123.7 (d, *J* = 3 Hz), 115.3 (d, *J* = 20 Hz), 114.0, 100.9 (d, *J* = 16 Hz), 61.5, 14.5. **^{19}F NMR** (376 MHz, $CDCl_3$) δ -105.64. NMR data is consistent with literature values.[18] **HRMS** (EI+) calcd for $[M]^+$ $C_{16}H_{16}O_2$: 269.0852, found: 269.0848. Melting point (ethyl acetate): 131 - 134 °C.

Ethyl 4'-methoxy-[1,1'-biphenyl]-4-carboxylate (30)

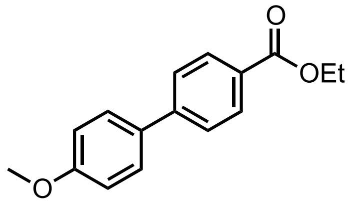

Prepared according to General Method 1. Purified by column chromatography (Ethyl Acetate/Petroleum ether = 1 - 3%) to give the product (0.182 g, 71%) as a white solid. **^{1}H NMR** (500 MHz, $CDCl_3$) δ 8.08 (d, *J* = 8.4 Hz, 2H), 7.62 (d, *J* = 8.4 Hz, 2H), 7.57 (d, *J* = 8.8 Hz, 2H), 7.00 (d, *J* = 8.8 Hz, 2H), 4.40 (q, *J* = 7.1 Hz, 2H), 3.86 (s, 3H), 1.41 (t, *J* = 7.1 Hz, 3H). **^{13}C NMR** (126 MHz, $CDCl_3$) δ 166.7, 160.0, 145.4, 132.6, 130.2, 128.8, 128.5, 126.6,

114.5, 61.1, 55.5, 14.5. NMR data is consistent with literature values.[19] **HRMS** (EI+) calcd for $[M]^+$ $C_{16}H_{16}O_3$: 256.1099, found: 256.1105. Melting point (ethyl acetate): 100 - 103 °C.

1-(3'-fluoro-2'-methyl-[1,1'-biphenyl]-4-yl)ethan-1-one (31)

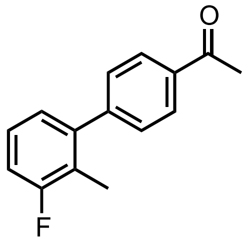

Prepared according to General Method 1. Initially purified by column chromatography (Ethyl Acetate/Petroleum ether = 0 - 10%) to give a mixture containing the desired product. The impurity was removed by Kugelrohr distillation (120 °C, 4 mbar) to yield the product as a pale yellow solid (0.140 g, 61%). **^{1}H NMR** (400 MHz, $CDCl_3$) δ 8.02 (d, *J* = 8.6 Hz, 2H), 7.41 (d, *J* = 8.6 Hz, 2H), 7.26 - 7.17 (m, 1H), 7.11 - 6.99 (m, 2H), 2.65 (s, 3H), 2.17 (d, *J* = 2.4 Hz, 3H). **^{13}C NMR** (101 MHz, $CDCl_3$) δ 197.9, 161.6 (d, *J* = 245 Hz), 145.6 (d, *J* = 3 Hz), 143.1 (d, *J* = 5 Hz), 136.0, 129.6, 128.4, 126.9 (d, *J* = 9 Hz), 125.1 (d, *J* = 3 Hz), 122.8 (d, *J* = 17 Hz), 114.5 (d, *J* = 23 Hz), 26.8, 12.2 (d, *J* = 5 Hz). **^{19}F NMR** (376 MHz, $CDCl_3$) δ -115.22. **HRMS** (EI+) calc. for $C_{15}H_{13}OF$: 228.0950, found 228.0959. **IR** ν 1674, 1605, 1566, 1458, 1404, 1350, 1265, 1234, 1111, 841, 787, 594 cm^{-1}. Melting point (ethyl acetate): 85 - 87 °C.

4’-methoxy-[1,1’-biphenyl]-4-carbonitrile (32)

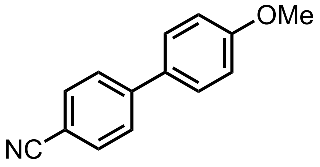

Prepared according to General Method 1. Purified by column chromatography (Ethyl Acetate/Petroleum ether = 10%) to give the product (0.107 g, 51%) as a white solid. **^{1}H NMR** (500 MHz, $CDCl_3$) δ 7.69 (d, *J* = 8.6 Hz, 2H), 7.64 (d, *J* = 8.6 Hz, 2H), 7.54 (d, *J* = 8.9 Hz, 2H), 7.01 (d, *J* = 8.8 Hz, 2H), 3.87 (s, 3H). **^{13}C NMR** (126 MHz, $CDCl_3$) δ 160.4, 145.4, 132.7, 131.7, 128.5, 127.3, 119.2, 114.7, 110.3, 55.6. NMR data is consistent with literature values.[20] **HRMS** (FTMS+) calcd for $[M+H]^+$ $C_{14}H_{11}NO$: 210.0913, found: 210.0912. Melting point (ethyl acetate): 100 - 102 °C.

2-(*p*-tolyl)thiophene (33)

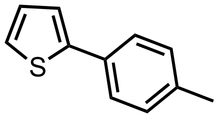

Prepared according to General Method 1. Purified by column chromatography (Ethyl Acetate/Petroleum ether = 0 - 5%) to give the product **(**0.137 g, 72%) as a blue solid. **^{1}H NMR** (500 MHz, $CDCl_3$) δ 7.51 (d, *J* = 8.0 Hz, 2H), 7.26 (dd, *J* = 12.5, 4.3 Hz, 2H), 7.19 (d, *J* = 8.0 Hz, 2H), 7.09 - 7.05 (m, 1H), 2.37 (s, 3H). **^{13}C NMR** (126 MHz, $CDCl_3$) δ 144.7, 137.5, 131.8, 129.7, 128.1, 126.0, 124.4, 122.7, 21.3. NMR data is consistent with literature values.[21] **HRMS** (ASAP+) calcd for $[M]^+$ $C_{11}H_{10}S$: 174.0503, found: 174.0505. Melting point (ethyl acetate): 64 - 66 °C.

4-(thiophen-2-yl)benzonitrile (34)

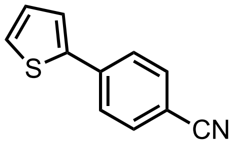

Prepared according to General Method 1. Purified by column chromatography (Ethyl Acetate/Petroleum ether = 1 - 5%) to give the product (0.157 g, 85%) as a yellow solid. **^{1}H NMR** (500 MHz, $CDCl_3$) δ 7.68 (dd, *J* = 22.2, 8.6 Hz, 4H), 7.41 (ddd, *J* = 6.1, 4.4, 1.0 Hz, 2H), 7.13 (dd, *J* = 5.1, 3.7 Hz, 1H). **^{13}C NMR** (126 MHz, $CDCl_3$) δ 142.2, 138.8, 132.9, 128.7, 127.2, 126.2, 125.3, 119.0, 110.7. NMR data is consistent with literature values.[22] **HRMS** (ASAP+) calcd for $[M+H]^+$ $C_{11}H_7NS$: 186.0377, found: 186.0383. Melting point (ethyl acetate): 92 - 94 °C.

Ethyl 4'-chloro-2'-fluoro-[1,1'-biphenyl]-4-carboxylate (35)

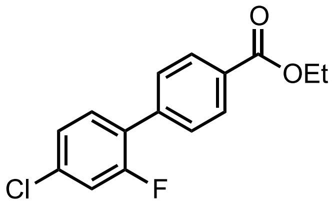

Prepared according to General Method 1. Purified by column chromatography (Ethyl Acetate/Petroleum ether = 10%) to give the product (0.122 g, 45%) as a white solid. **^{1}H NMR** (500 MHz, $CDCl_3$) δ 8.14 - 8.09 (m, 2H), 7.58 (dd, *J* = 8.6 Hz, 1.7, 2H), 7.40 (dd, *J* = 12.2, 4.4 Hz, 1H), 7.25 - 7.19 (m, 2H), 4.41 (q, *J* = 7.1 Hz, 2H), 1.41 (t, *J* = 7.1 Hz, 3H). **^{13}C NMR** (126 MHz, $CDCl_3$) δ 166.4, 159.6 (d, *J* = 253 Hz), 139.3 (d, *J* = 1 Hz), 134.9 (d, *J* = 10 Hz), 131.5 (d, *J* = 4 Hz), 130.1, 129.9, 129.0 (d, *J* = 3 Hz), 126.9 (d, *J* = 13 Hz), 125.1 (d, *J* = 4 Hz), 117.2 (d, *J* = 26 Hz), 61.2, 14.5. **^{19}F NMR** (376 MHz, $CDCl_3$) δ -114.764. **HRMS** (EI+) calcd for $[M]^+$ $C_{15}H_{12}ClFO_2$: 278.0510, found: 278.0515. **IR** ν 2361, 1705, , 1265, 1211, 1180, 1096, 1026, 1003, 895, 856, 818, 764, 718, 702 cm^{-1}. Melting point (ethyl acetate): 37 - 38 °C.

Ethyl 4'-chloro-[1,1'-biphenyl]-4-carboxylate (36)

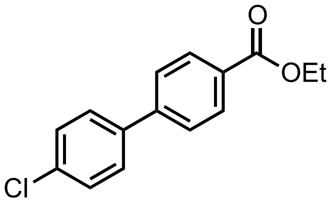

Prepared according to General Method 1. Purified by column chromatography (Ethyl Acetate/Petroleum ether = 2.5%) to give the product (0.167 g, 63%) as a white solid. **^{1}H NMR** (500 MHz, $CDCl_3$) δ 8.11 (d, *J* = 8.4 Hz, 2H), 7.62 (d, *J* = 8.4 Hz, 2H), 7.55 (d, *J* = 8.5 Hz, 2H), 7.44 (d, *J* = 8.5 Hz, 2H), 4.40 (q, *J* = 7.1 Hz, 2H), 1.42 (t, *J* = 7.1 Hz, 3H). **^{13}C NMR** (126 MHz, $CDCl_3$) δ 166.5, 144.4, 138.6, 134.5, 130.3, 129.7, 129.3, 128.7, 127.0, 61.2, 14.5. NMR data is consistent with literature values.[23] **HRMS** (ASAP+) calcd for $[M+H]^+$ $C_{15}H_{13}ClO_2$: 261.0687, found: 261.0687. Melting point (ethyl acetate): 66 - 69 °C.

4'-methoxy-[1,1'-biphenyl]-4-carbonitrile (37)

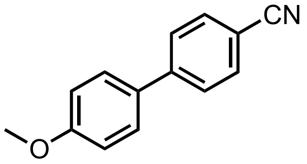

Prepared according to General Method 1. Purified by column chromatography (Ethyl Acetate/Petroleum ether = 10%) to give the product (0.130 g, 62%) as a white solid. **^{1}H NMR** (500 MHz, $CDCl_3$) δ 7.69 (d, *J* = 8.6 Hz, 2H), 7.64 (d, *J* = 8.6 Hz, 2H), 7.54 (d, *J* = 8.9 Hz, 2H), 7.01 (d, *J* = 8.8 Hz, 2H), 3.87 (s, 3H). **^{13}C NMR** (126 MHz, $CDCl_3$) δ 160.4, 145.4, 132.7, 131.7, 128.5, 127.3, 119.2, 114.7, 110.3, 55.6. NMR data is consistent with literature values.[20] **HRMS** (FTMS+) calcd for $[M+H]^+$ $C_{14}H_{11}NO$: 210.0913, found: 210.0912. Melting point (ethyl acetate): 100 - 102 °C.

Ethyl 4'-cyano-[1,1'-biphenyl]-4-carboxylate (38)

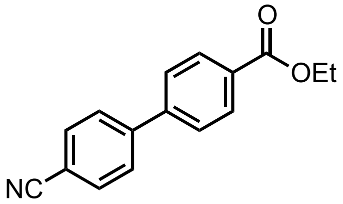

Prepared according to General Method 1. Purified by column chromatography (Ethyl Acetate/Petroleum ether = 10%) to give the product (0.227 g, 90%) as a white solid. **^{1}H NMR** (500 MHz, $CDCl_3$) δ 8.15 (d, *J* = 8.5 Hz, 2H), 7.76 (d, *J* = 8.5 Hz, 2H), 7.72 (d, *J* = 8.5 Hz, 2H), 7.65 (d, *J* = 8.5 Hz, 2H), 4.42 (q, *J* = 7.1 Hz, 2H), 1.42 (t, *J* = 7.1 Hz, 3H). **^{13}C NMR** (126 MHz, $CDCl_3$) δ 166.3, 144.6, 143.5, 132.9, 130.7, 130.5, 128.1, 127.3, 118.8, 111.9, 61.4, 14.5. NMR data is consistent with literature values.[24] **HRMS** (ASAP+) calcd for $[M]^+$ $C_{16}H_{13}NO_2$: 251.0946, found: 251.0947. Melting point (ethyl acetate): 117 - 120 °C

2'-methoxy-[1,1'-biphenyl]-4-carbonitrile (39)

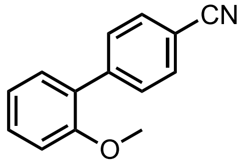

Prepared according to General Method 1. Purified by column chromatography (Ethyl Acetate/Petroleum ether = 7.5%) to give the product (0.148 g, 71%) as a white solid. **^{1}H NMR** (500 MHz, $CDCl_3$) δ 7.69 (d, *J* = 8.6 Hz, 2H), 7.64 (d, *J* = 8.6 Hz, 2H), 7.39 (ddd, *J* = 8.3, 7.5, 1.7 Hz, 1H), 7.30 (dd, *J* = 7.5, 1.7 Hz, 1H), 7.06 (td, *J* = 7.5, 1.0 Hz, 1H), 7.01 (d, *J* = 8.3 Hz, 1H), 3.83 (s, 3H). **^{13}C NMR** (126 MHz, $CDCl_3$) δ 156.5, 143.5, 131.9, 130.8, 130.4, 130.1, 128.8, 121.2, 119.3, 111.5, 110.6, 55.7. NMR data is consistent with literature values.[25] **HRMS** (ASAP+) calcd for $[M+H]^+$ $C_{14}H_{11}NO$: 210.0913, found: 210.0912. Melting point (ethyl acetate): 77 - 78 °C.

Ethyl 4-(thiophen-2-yl)benzoate (40)

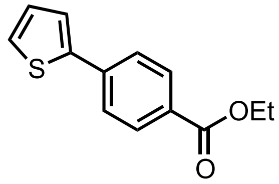

Prepared according to General Method 1. Purified by column chromatography (Ethyl Acetate/Petroleum ether = 5%) to give the product (0.195 g, 86%) as a white solid. **^{1}H NMR** (500 MHz, $CDCl_3$) δ 8.05 (d, *J* = 8.6 Hz, 2H), 7.67 (d, *J* = 8.6 Hz, 2H), 7.42 (dd, *J* = 3.6, 1.1 Hz, 1H), 7.36 (dd, *J* = 5.1, 1.1 Hz, 1H), 7.12 (dd, *J* = 5.1, 3.6 Hz, 1H), 4.39 (q, *J* = 7.1 Hz, 2H), 1.41 (t, *J* = 7.1 Hz, 3H). **^{13}C NMR** (126 MHz, $CDCl_3$) δ 166.4, 143.3, 138.7, 130.4, 129.3, 128.5, 126.4, 125.6, 124.6, 61.1, 14.5. NMR data is consistent with literature values.[22] **HRMS** (ASAP+) calcd for $[M+H]^+$ $C_{13}H_{12}O_2S$: 233.0631, found: 233.0631. Melting point (ethyl acetate): 64 - 66 °C.

3-phenylpyridine (41)

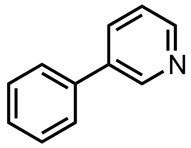

Prepared according to General Method 1. Purified by column chromatography ((Ethyl Acetate/Petroleum ether = 0 - 10%) to yield the product as a brown oil (0.150 g, 97%).**^{1}H NMR** (400 MHz, $CDCl_3$) δ 8.85 (s, 1H), 8.60 (d, *J* = 3.7 Hz, 1H), 7.88 (ddd, *J* = 7.9, 2.4, 1.6 Hz, 1H), 7.62 – 7.56 (m, 2H), 7.51 - 7.46 (m, 2H), 7.43 - 7.35 (m, 2H). **^{13}C NMR** (101 MHz, $CDCl_3$) δ 148.6, 148.5, 138.0, 136.8, 134.5, 129.2, 128.2, 127.3, 123.7. NMR data is consistent with literature values.[26] **HRMS** (EI+) calc. for $C_{11}H_9N$: 155.0735, found 155.0737.

6 NMR data

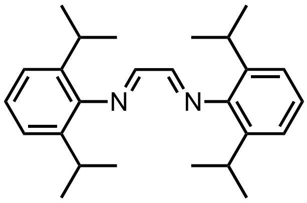
N
N

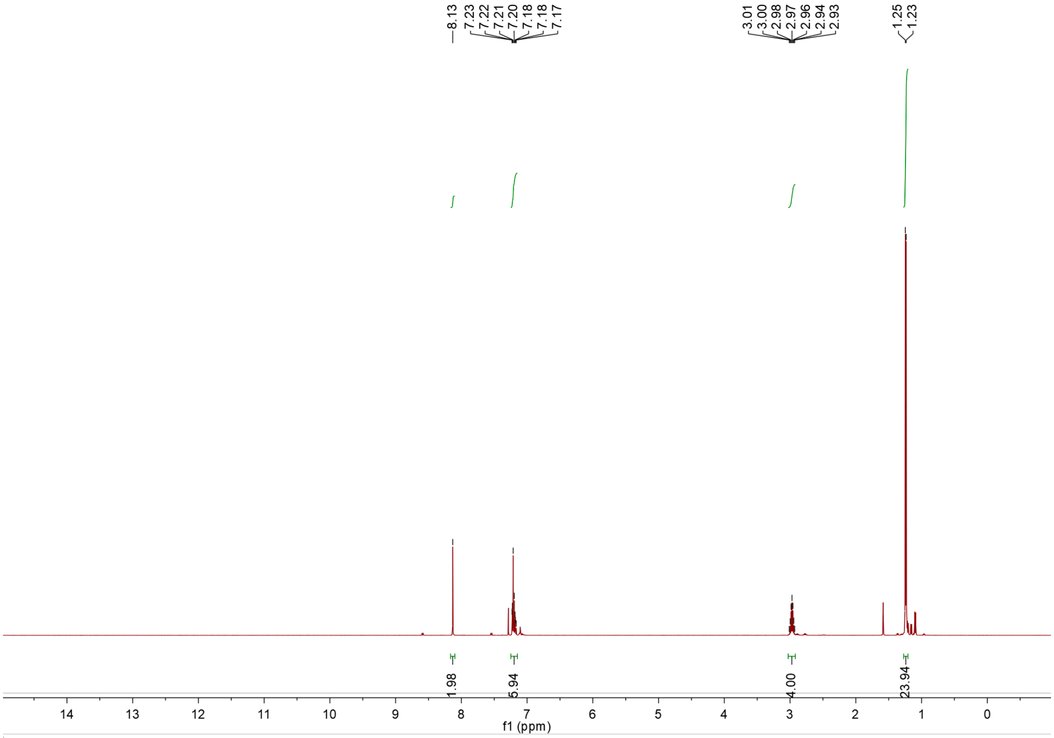
8.13
7.23
7.22
7.21
7.20
7.18
7.17
3.01
3.00
2.98
2.97
2.96
2.94
2.93
1.25
1.23
1.98
5.94
4.00
23.94
14 13 12 11 10 9 8 7 6 5 4 3 2 1 0
f1 (ppm)

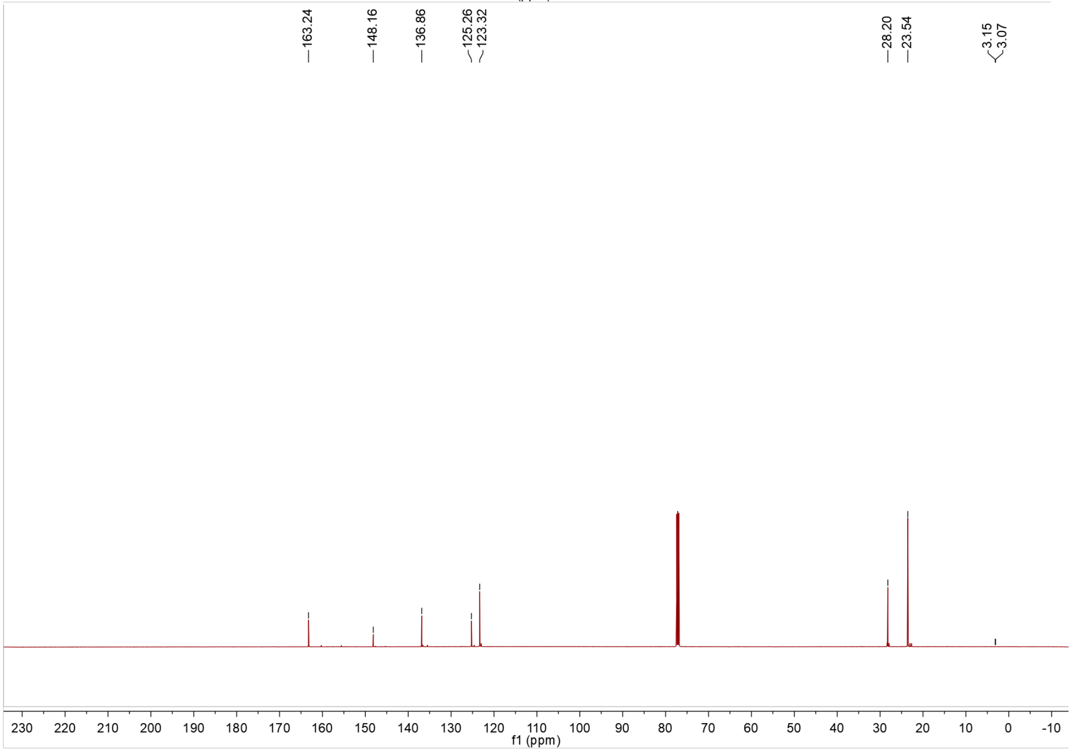
163.24
148.16
136.86
125.26
123.32
28.20
23.54
3.15
3.07
230 220 210 200 190 180 170 160 150 140 130 120 110 100 90 80 70 60 50 40 30 20 10 0 -10
f1 (ppm)

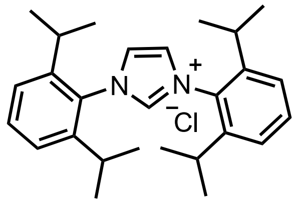
N
N
+
-
Cl

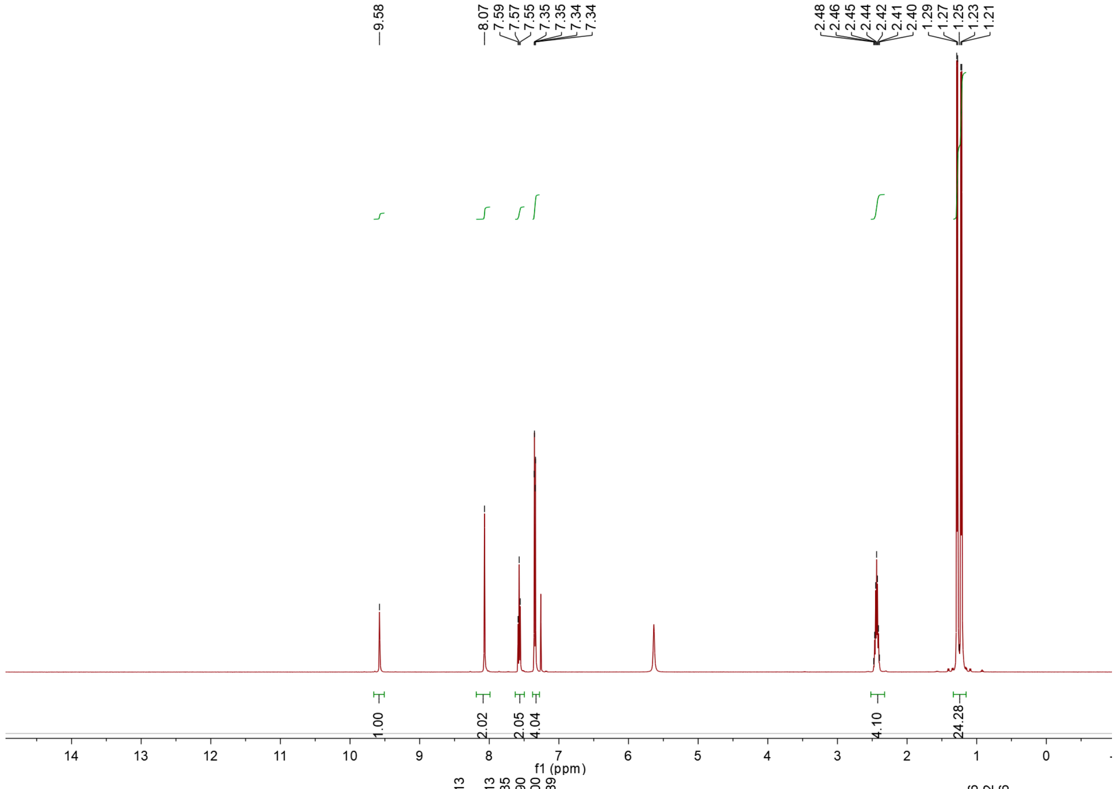
9.58
8.07
7.59
7.57
7.55
7.35
7.35
7.34
7.34
2.48
2.46
2.45
2.44
2.42
2.41
2.40
1.29
1.27
1.25
1.23
1.21
1.00
2.02
2.05
4.04
4.10
24.28
14
13
12
11
10
9
8
7
6
5
4
3
2
1
0
f1 (ppm)

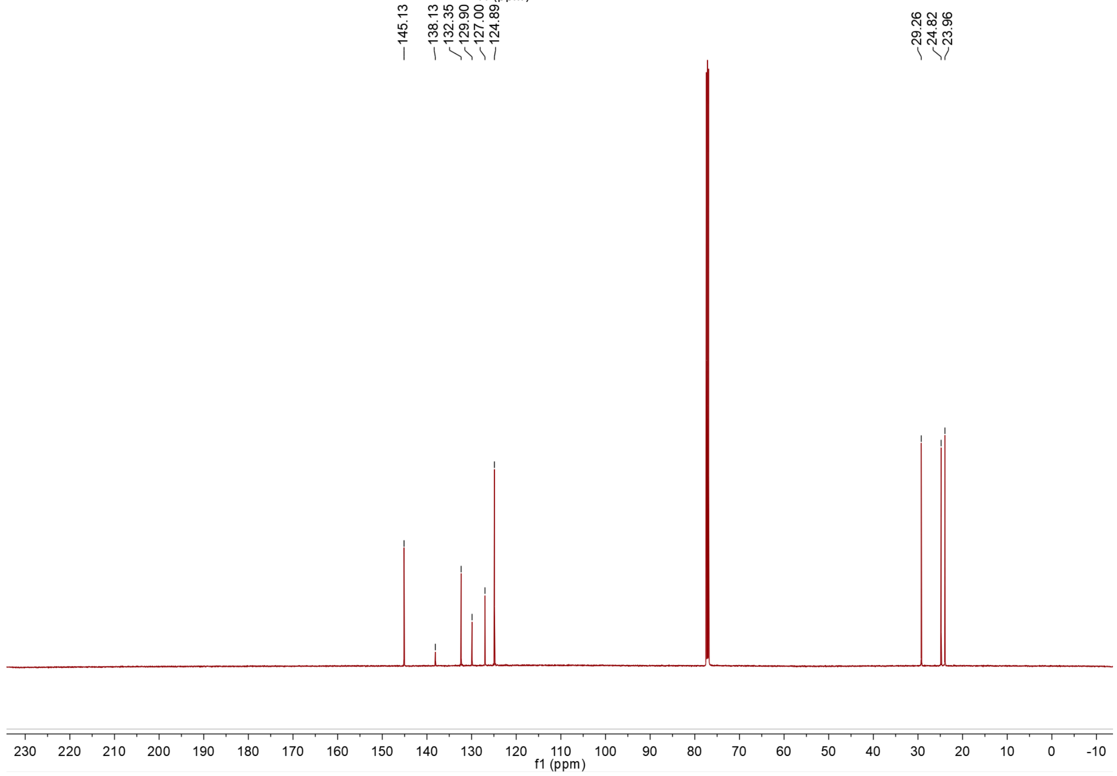
145.13
138.13
132.35
129.90
127.00
124.89
29.26
24.82
23.96
230
220
210
200
190
180
170
160
150
140
130
120
110
100
90
80
70
60
50
40
30
20
10
0
-10
f1 (ppm)

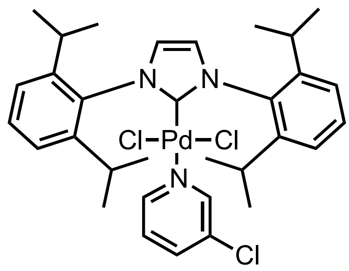
N
N
Cl
Pd
Cl
N
Cl

6

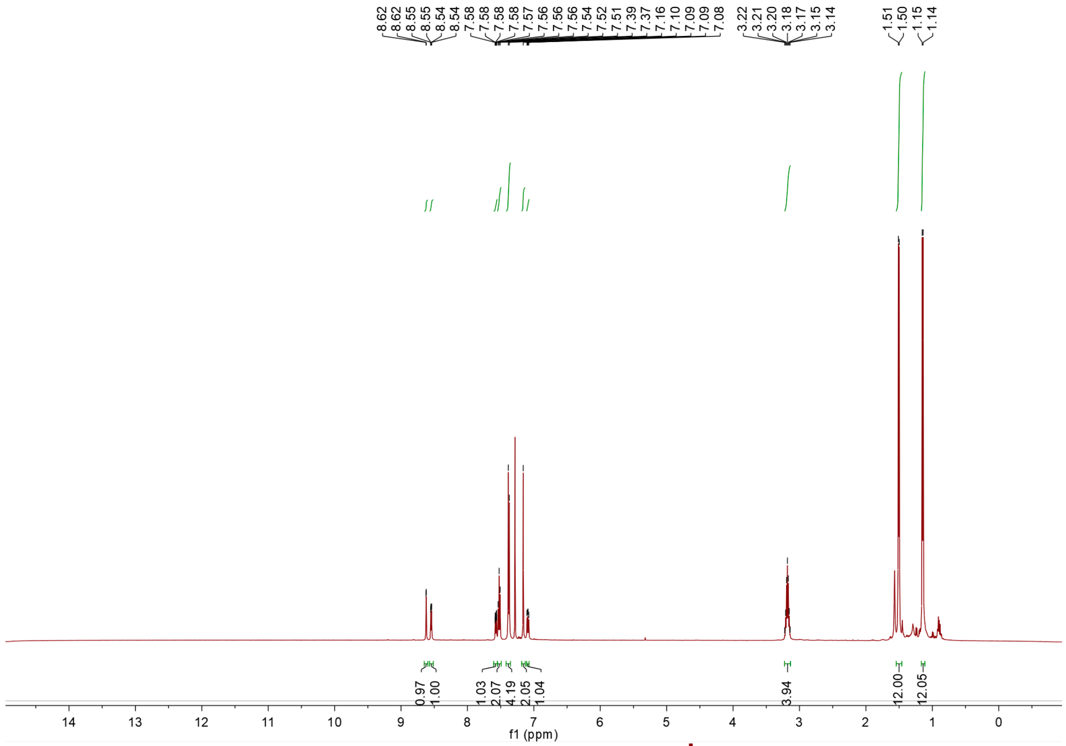
8.62
8.62
8.55
8.55
8.54
8.54
7.58
7.58
7.58
7.58
7.57
7.56
7.56
7.56
7.54
7.52
7.51
7.39
7.37
7.16
7.10
7.09
7.09
7.08
3.22
3.21
3.20
3.18
3.17
3.15
3.14
1.51
1.50
1.15
1.14
0.97
1.00
1.03
2.07
4.19
2.05
1.04
3.94
12.00
12.05
f1 (ppm)

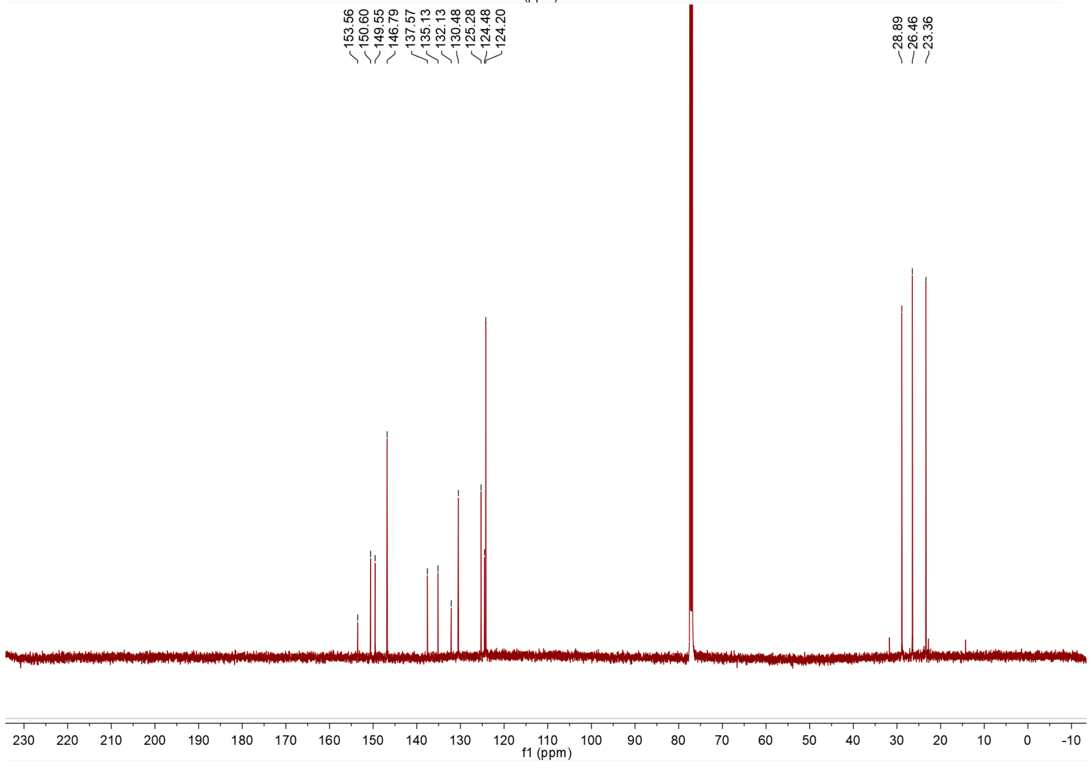
153.56
150.60
149.55
146.79
137.57
135.13
132.13
130.48
125.28
124.48
124.20
28.89
26.46
23.36
f1 (ppm)

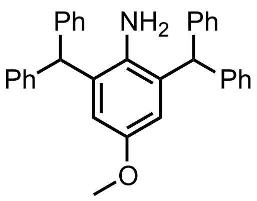
Ph
NH2
Ph
Ph
Ph
O

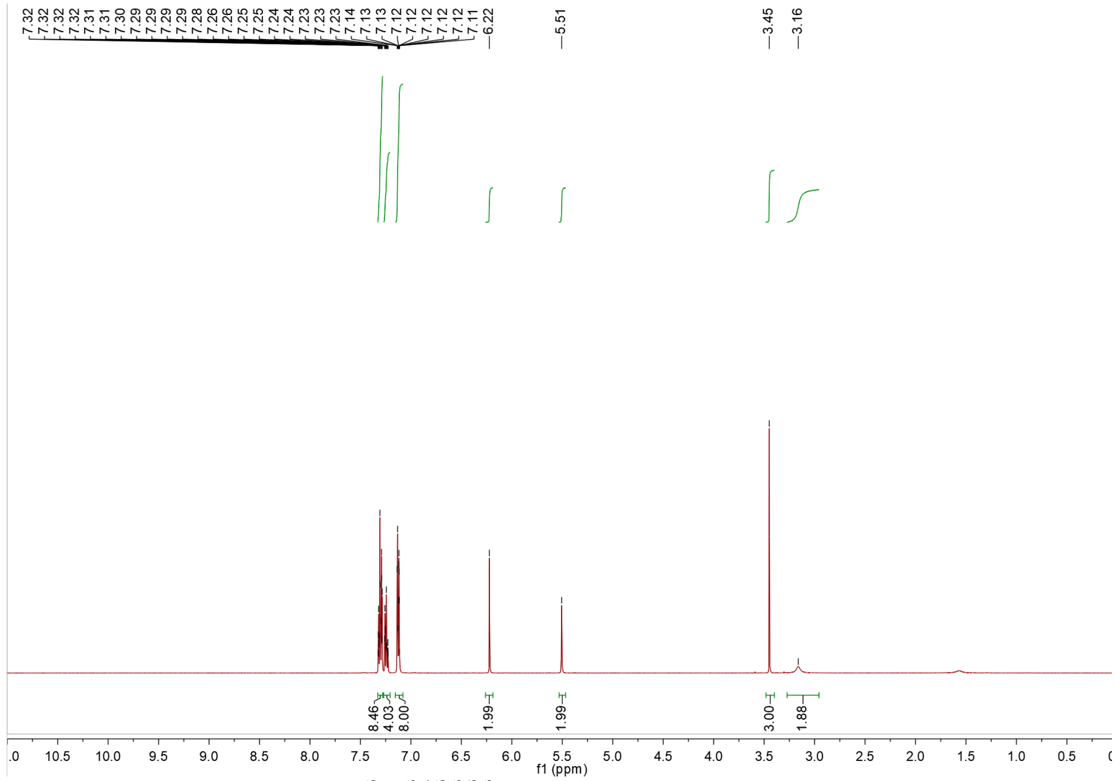
7.32
7.32
7.32
7.32
7.31
7.30
7.29
7.29
7.29
7.29
7.28
7.26
7.26
7.25
7.25
7.24
7.24
7.23
7.23
7.23
7.14
7.13
7.13
7.12
7.12
7.12
7.12
7.12
7.11
6.22
5.51
3.45
3.16
8.46
4.03
8.00
1.99
1.99
3.00
1.88
f1 (ppm)

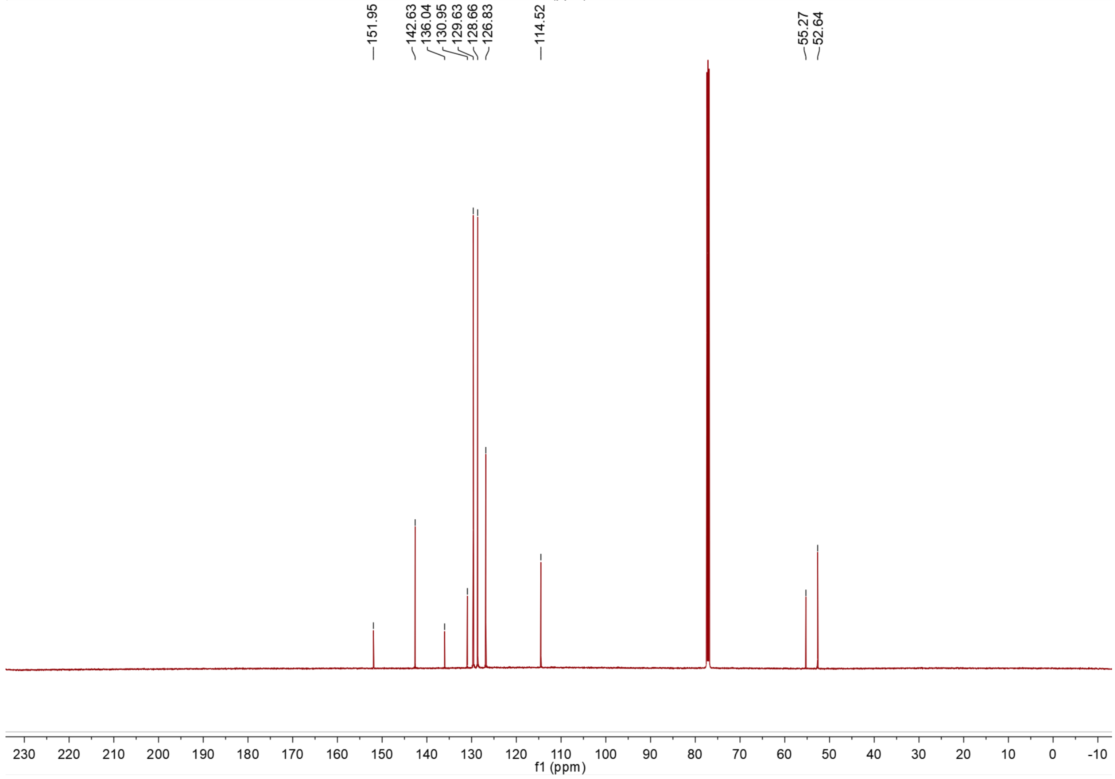
151.95
142.63
136.04
130.95
129.63
128.66
126.83
114.52
55.27
52.64
f1 (ppm)

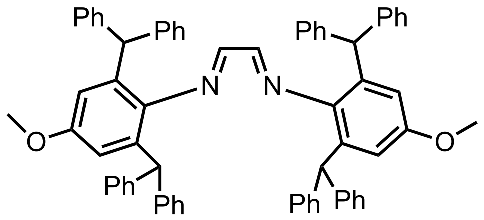
Ph
Ph
N
N
O
O

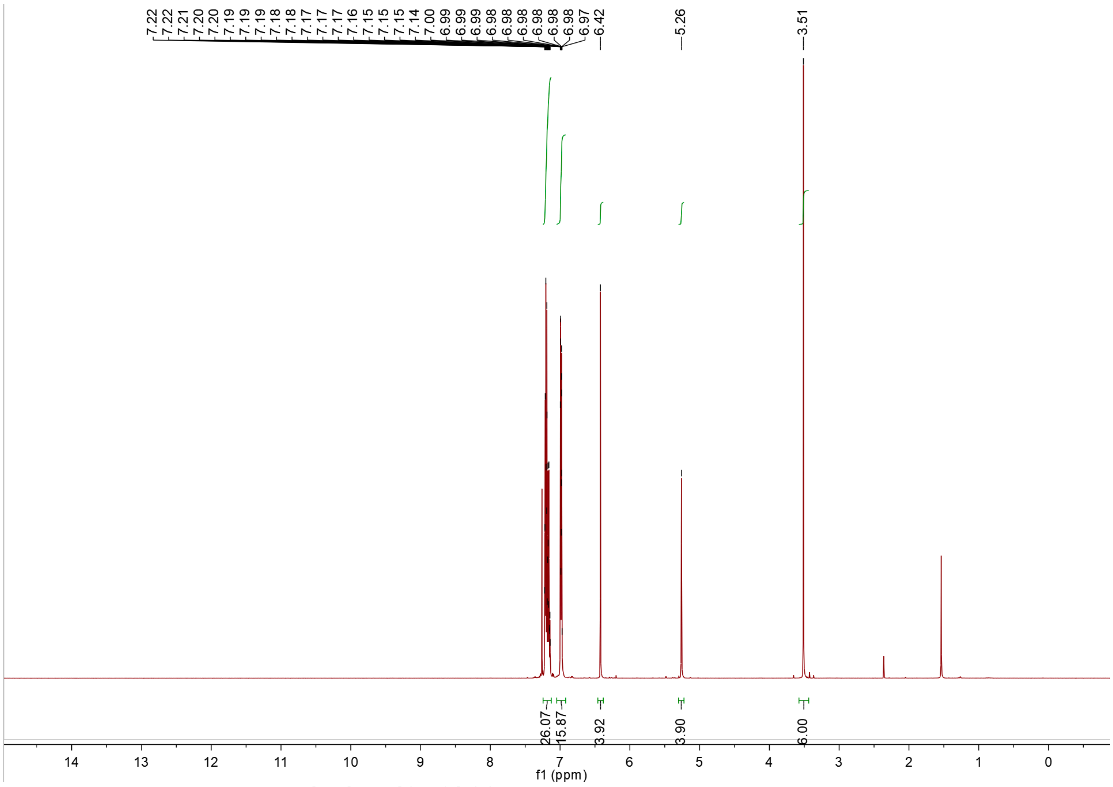
7.22
7.22
7.21
7.20
7.20
7.19
7.19
7.19
7.18
7.18
7.17
7.17
7.16
7.15
7.15
7.15
7.14
7.00
6.99
6.99
6.99
6.98
6.98
6.98
6.98
6.98
6.98
6.98
6.97
6.42
5.26
3.51
26.07
15.87
3.92
3.90
6.00
14
13
12
11
10
9
8
7
6
5
4
3
2
1
0
f1 (ppm)

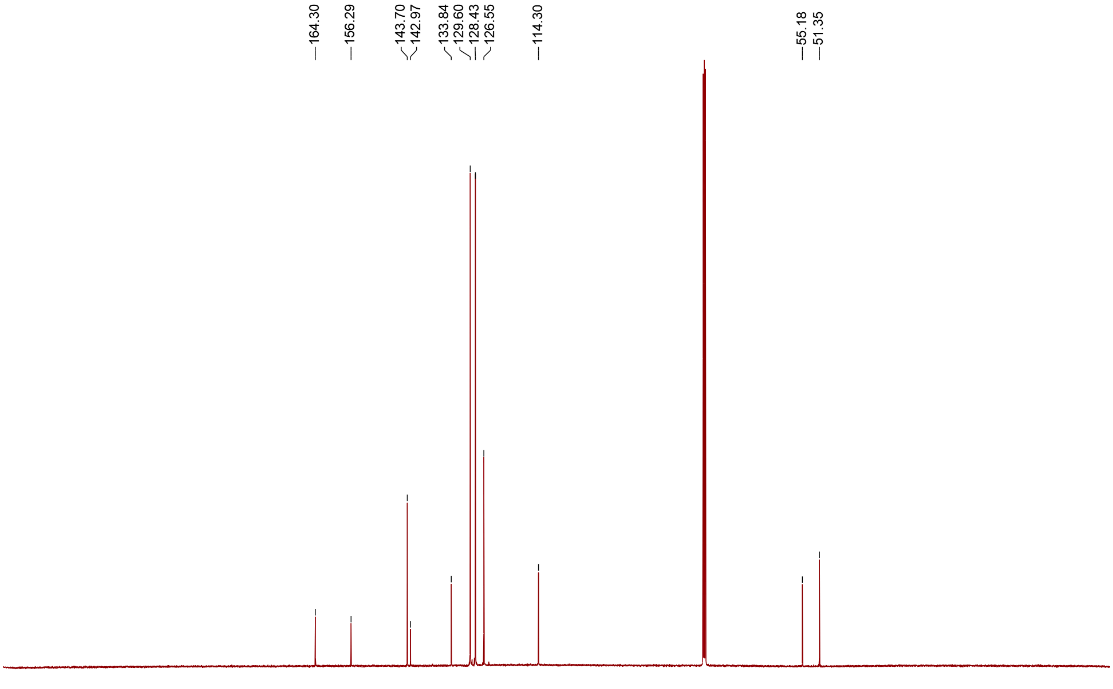
164.30
156.29
143.70
142.97
133.84
129.60
128.43
126.55
114.30
55.18
51.35
230
220
210
200
190
180
170
160
150
140
130
120
110
100
90
80
70
60
50
40
30
20
10
0
-10
f1 (ppm)

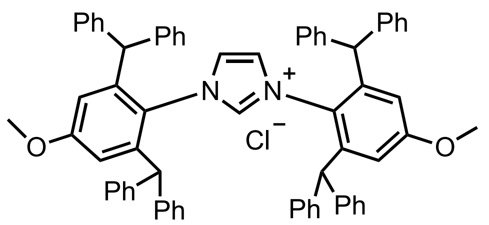
Ph
Ph
Ph
Ph
N
N
+
Cl
−
O
O
Ph
Ph
Ph
Ph

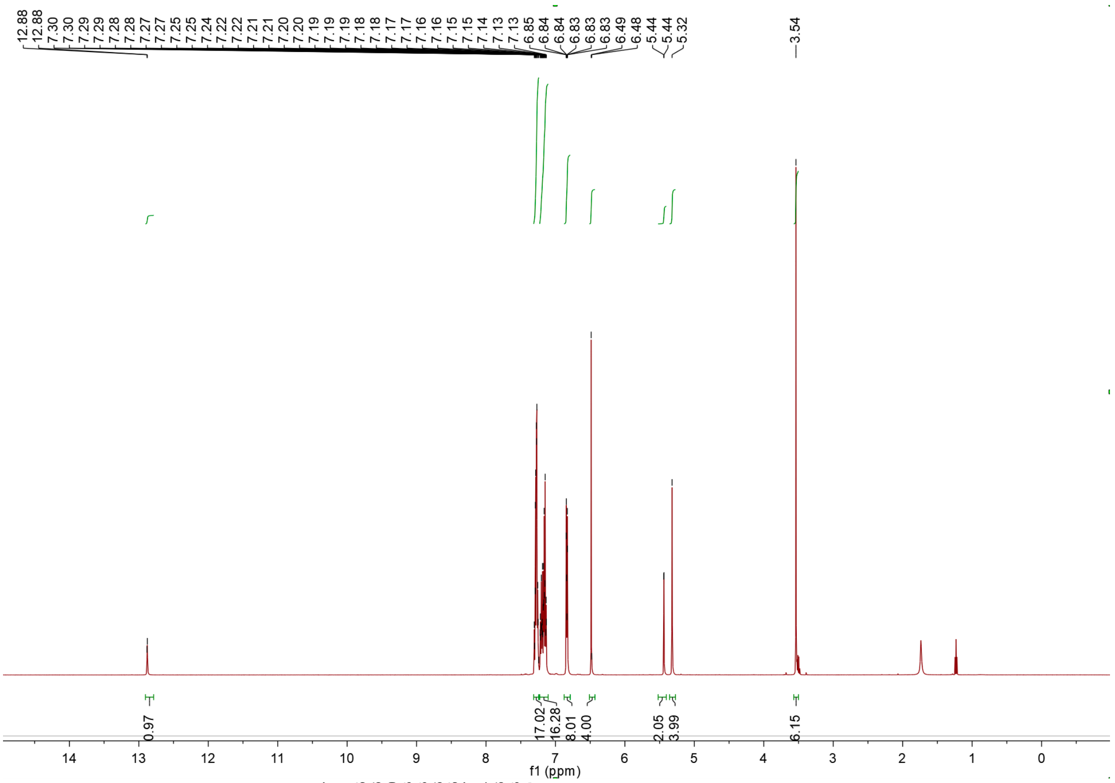
12.88
12.88
7.30
7.29
7.29
7.28
7.28
7.27
7.27
7.25
7.25
7.24
7.22
7.22
7.21
7.21
7.20
7.20
7.19
7.19
7.19
7.18
7.18
7.17
7.17
7.16
7.16
7.15
7.15
7.14
7.13
7.13
6.85
6.84
6.84
6.83
6.83
6.83
6.49
6.48
5.44
5.44
5.32
3.54
0.97
17.02
16.28
8.01
4.00
2.05
3.99
6.15
f1 (ppm)

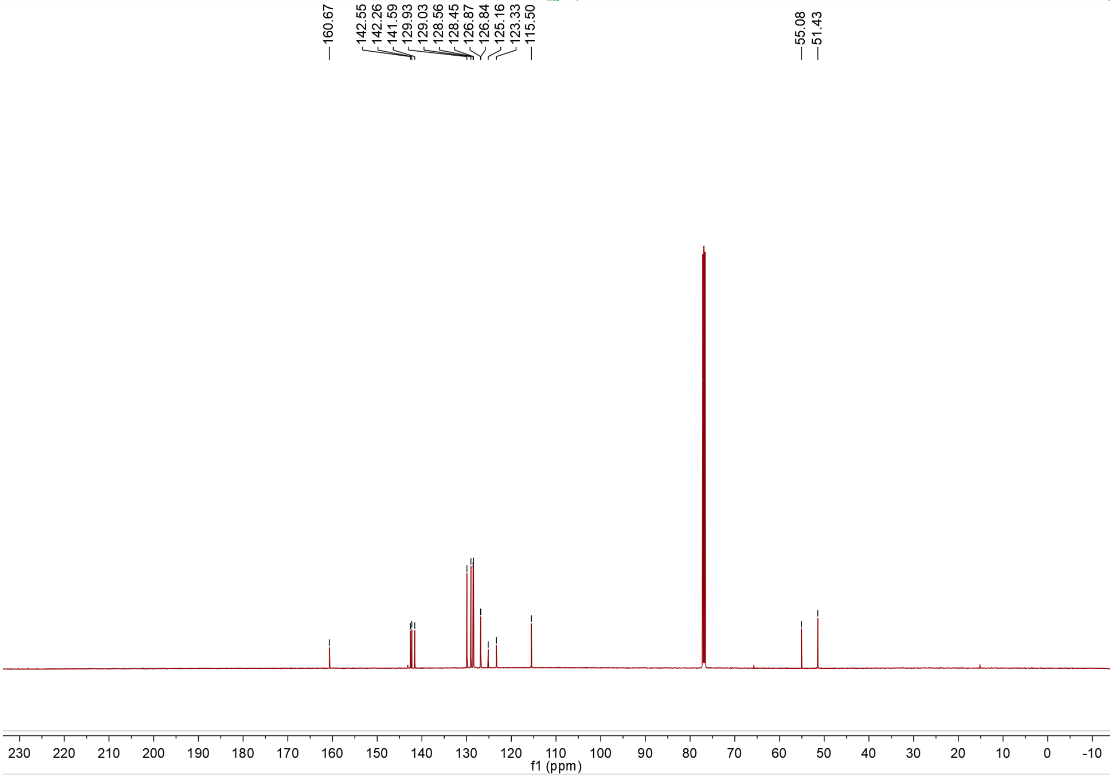
160.67
142.55
142.26
141.59
129.93
129.03
128.56
128.45
126.87
126.84
125.16
123.33
115.50
55.08
51.43
f1 (ppm)

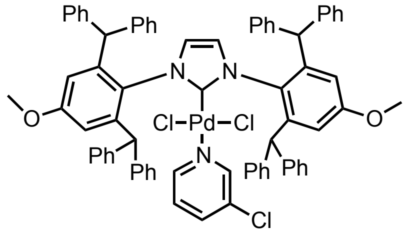

Ph
Ph
Ph
Ph
N
N
Cl
Pd
Cl
N
O
O
Ph
Ph
Ph
Ph
Cl

8

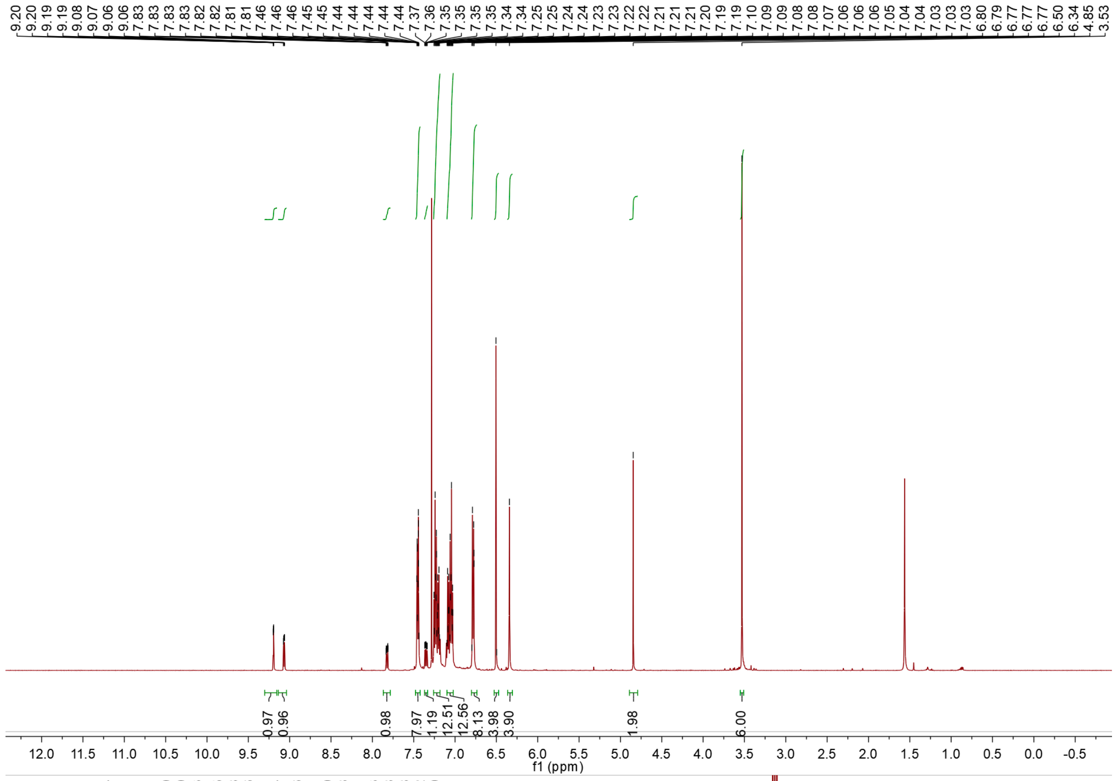

9.20 9.20 9.19 9.19 9.08 9.07 9.06 9.06 7.83 7.83 7.83 7.83 7.82 7.81 7.81 7.46 7.46 7.46 7.45 7.45 7.44 7.44 7.44 7.44 7.44 7.37 7.36 7.35 7.35 7.35 7.35 7.34 7.25 7.24 7.24 7.23 7.23 7.22 7.22 7.21 7.21 7.20 7.19 7.19 7.10 7.09 7.09 7.08 7.08 7.07 7.06 7.06 7.06 7.05 7.04 7.04 7.04 7.03 7.03 6.80 6.79 6.77 6.77 6.50 6.34 4.85 3.53
0.97 0.96 0.98 7.97 1.19 12.51 12.56 8.13 3.98 3.90 1.98 6.00
12.0 11.5 11.0 10.5 10.0 9.5 9.0 8.5 8.0 7.5 7.0 6.5 6.0 5.5 5.0 4.5 4.0 3.5 3.0 2.5 2.0 1.5 1.0 0.5 0.0 -0.5
f1 (ppm)

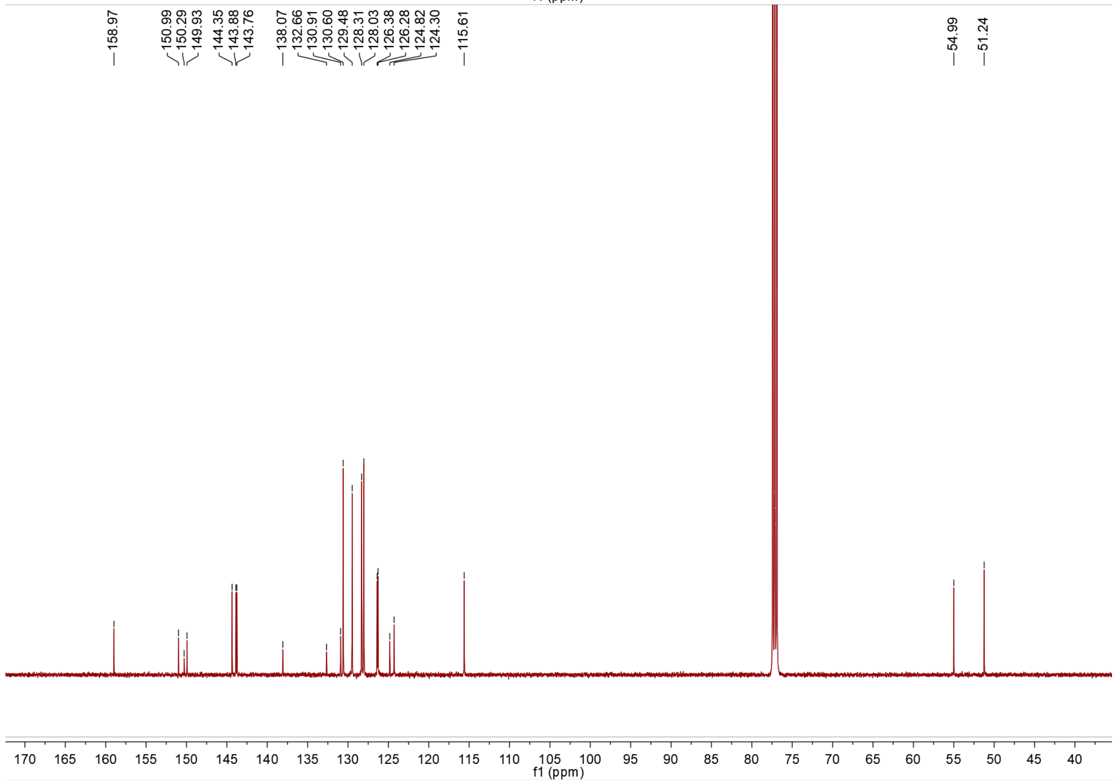

158.97
150.99 150.29 149.93 144.35 143.88 143.76
138.07 132.66 130.91 130.60 129.48 128.31 128.03 126.38 126.28 124.82 124.30
115.61
54.99 51.24
170 165 160 155 150 145 140 135 130 125 120 115 110 105 100 95 90 85 80 75 70 65 60 55 50 45 40
f1 (ppm)

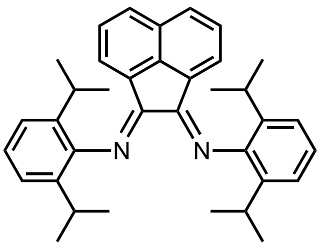
N
N

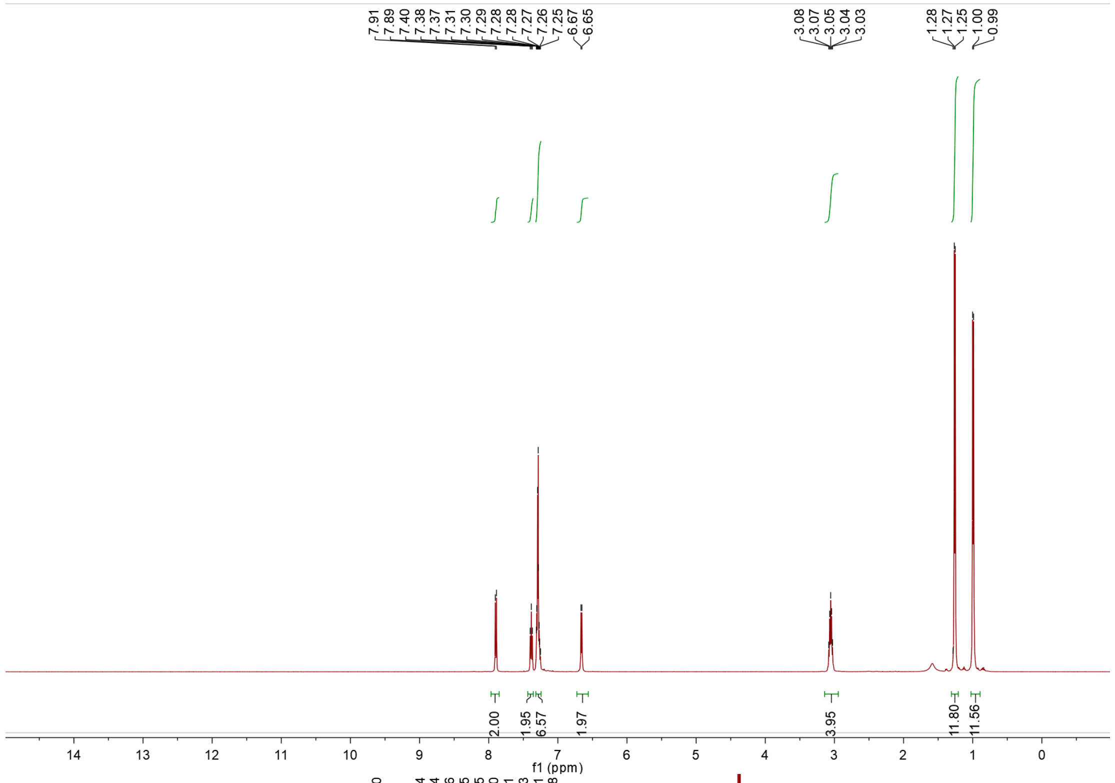
7.91
7.89
7.40
7.38
7.37
7.31
7.30
7.29
7.28
7.28
7.27
7.26
7.25
6.67
6.65
3.08
3.07
3.05
3.04
3.03
1.28
1.27
1.25
1.00
0.99
2.00
1.95
6.57
1.97
3.95
11.80
11.56
14 13 12 11 10 9 8 7 6 5 4 3 2 1 0
f1 (ppm)

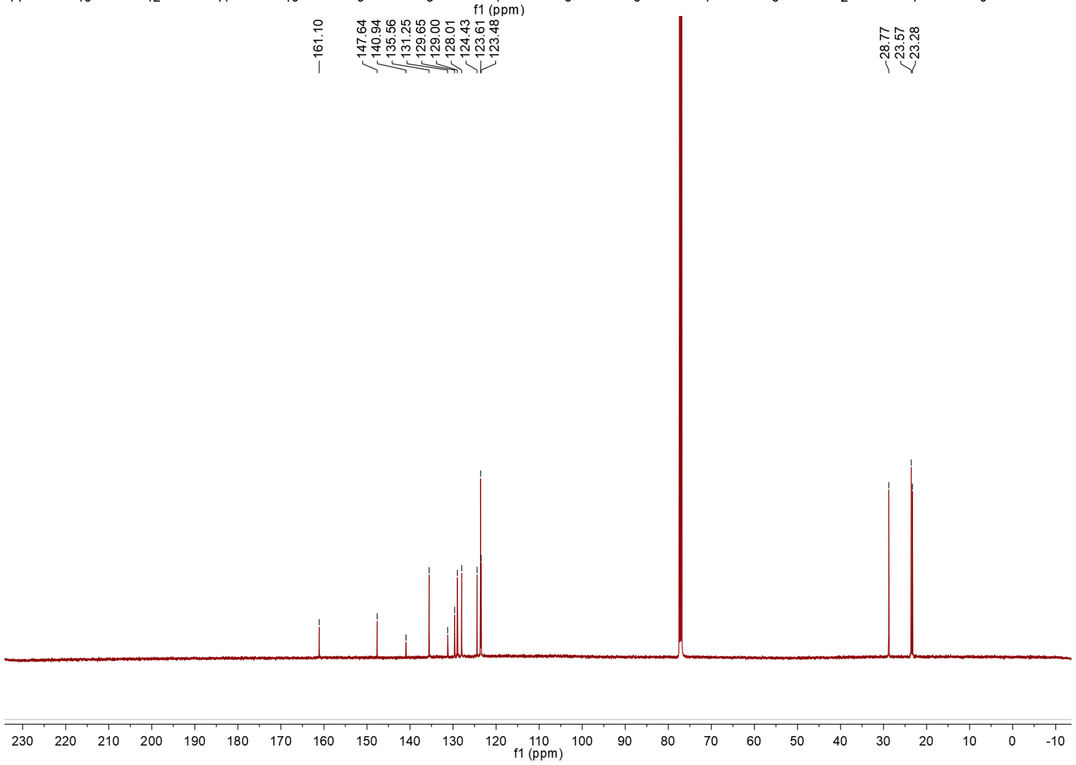
161.10
147.64
140.94
135.56
131.25
129.65
129.00
128.01
124.43
123.61
123.48
28.77
23.57
23.28
230 220 210 200 190 180 170 160 150 140 130 120 110 100 90 80 70 60 50 40 30 20 10 0 -10
f1 (ppm)

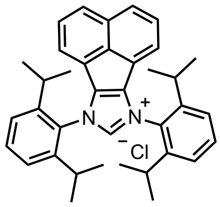
N
N
+
-
Cl

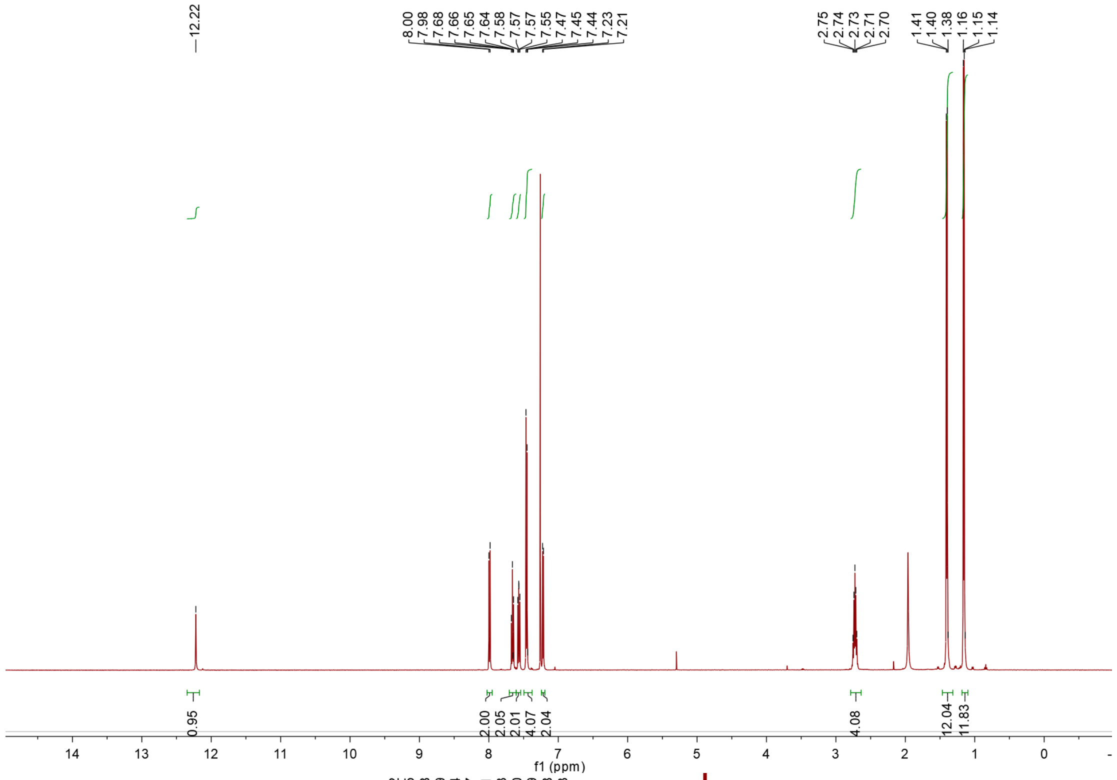
12.22
8.00
7.98
7.68
7.66
7.65
7.64
7.58
7.57
7.57
7.55
7.47
7.45
7.44
7.23
7.21
2.75
2.74
2.73
2.71
2.70
1.41
1.40
1.38
1.16
1.15
1.14
0.95
2.00
2.05
2.01
4.07
2.04
4.08
12.04
11.83
f1 (ppm)

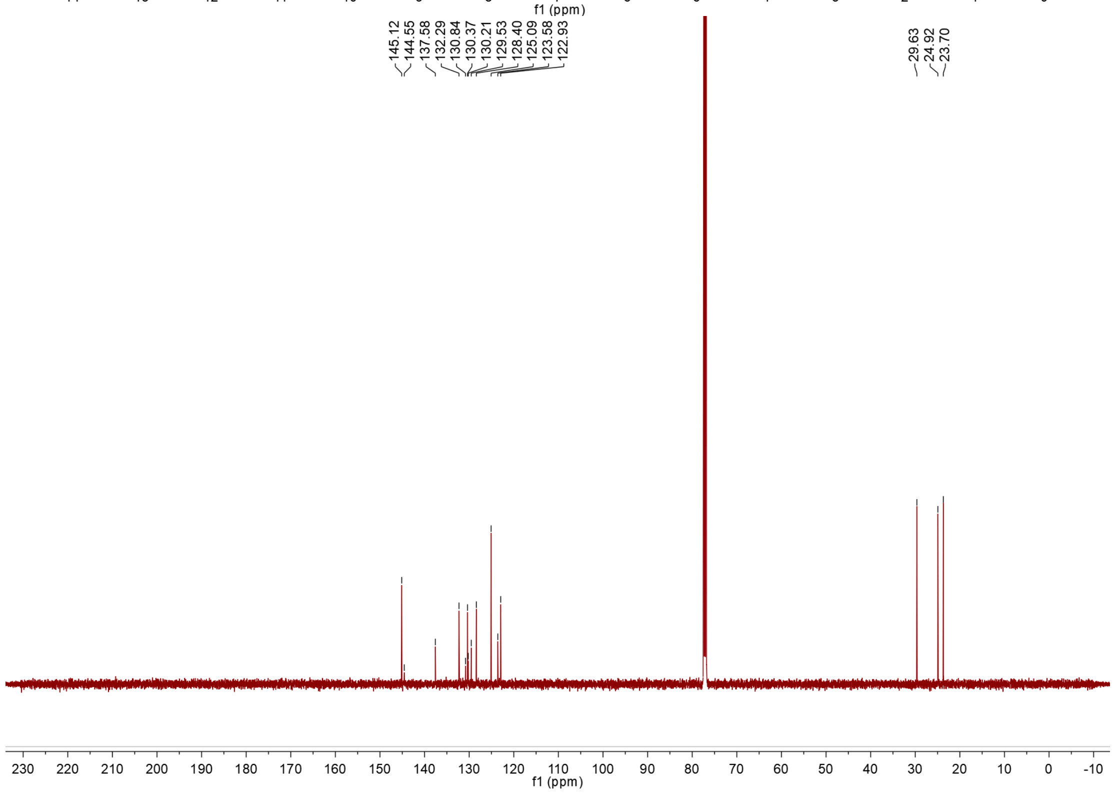
145.12
144.55
137.58
132.29
130.84
130.37
130.21
129.53
128.40
125.09
123.58
122.93
29.63
24.92
23.70
f1 (ppm)

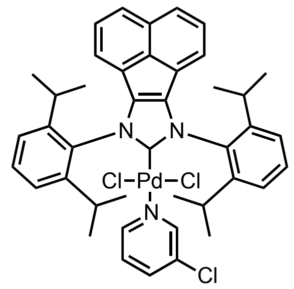

9

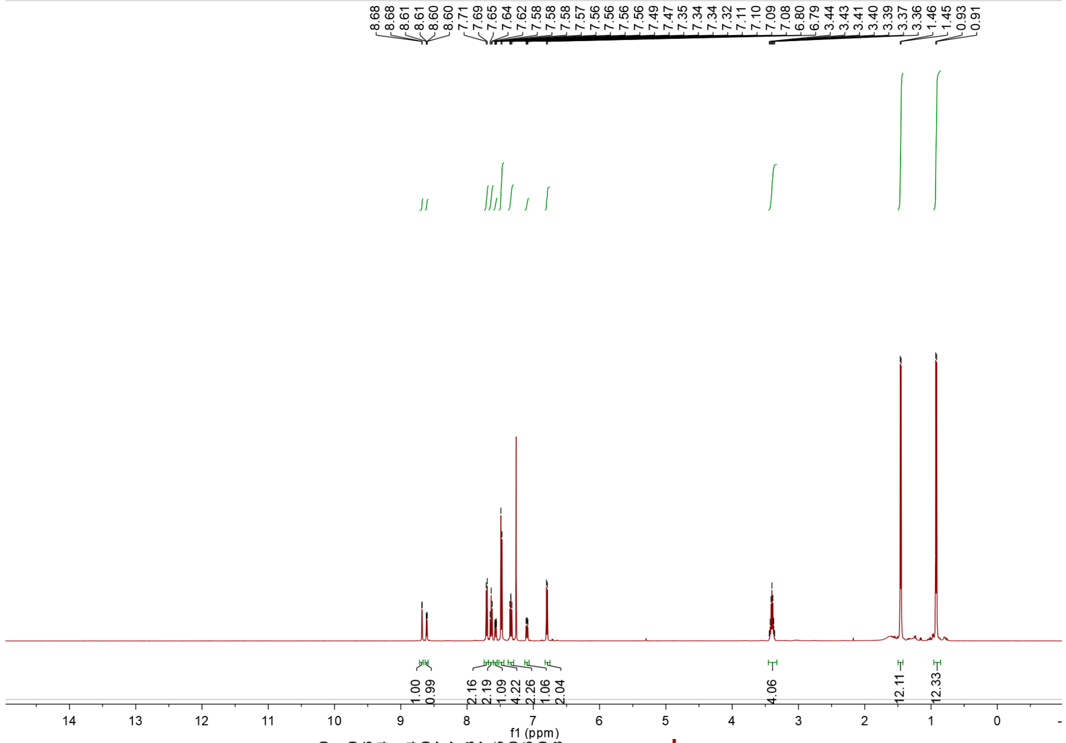
8.68
8.68
8.61
8.61
8.60
7.71
7.69
7.65
7.64
7.62
7.58
7.58
7.58
7.57
7.56
7.56
7.56
7.56
7.49
7.47
7.35
7.34
7.34
7.32
7.11
7.10
7.09
7.08
6.80
6.79
3.44
3.43
3.41
3.40
3.39
3.37
3.36
1.46
1.45
0.93
0.91
1.00
0.99
2.16
2.19
1.09
4.22
2.26
1.06
2.04
4.06
12.11
12.33
f1 (ppm)

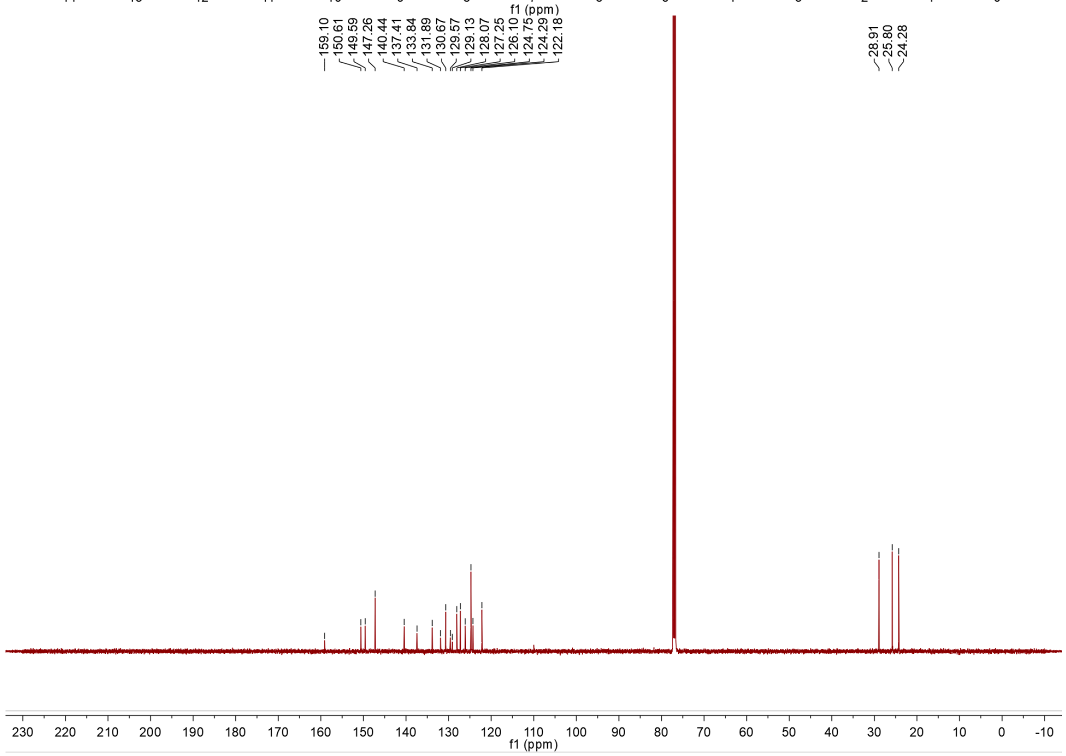
159.10
150.61
149.59
147.26
140.44
137.41
133.84
131.89
130.67
129.57
129.13
128.07
127.25
126.10
124.75
124.29
122.18
28.91
25.80
24.28
f1 (ppm)

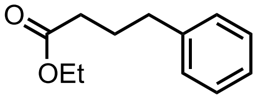
O
OEt

5

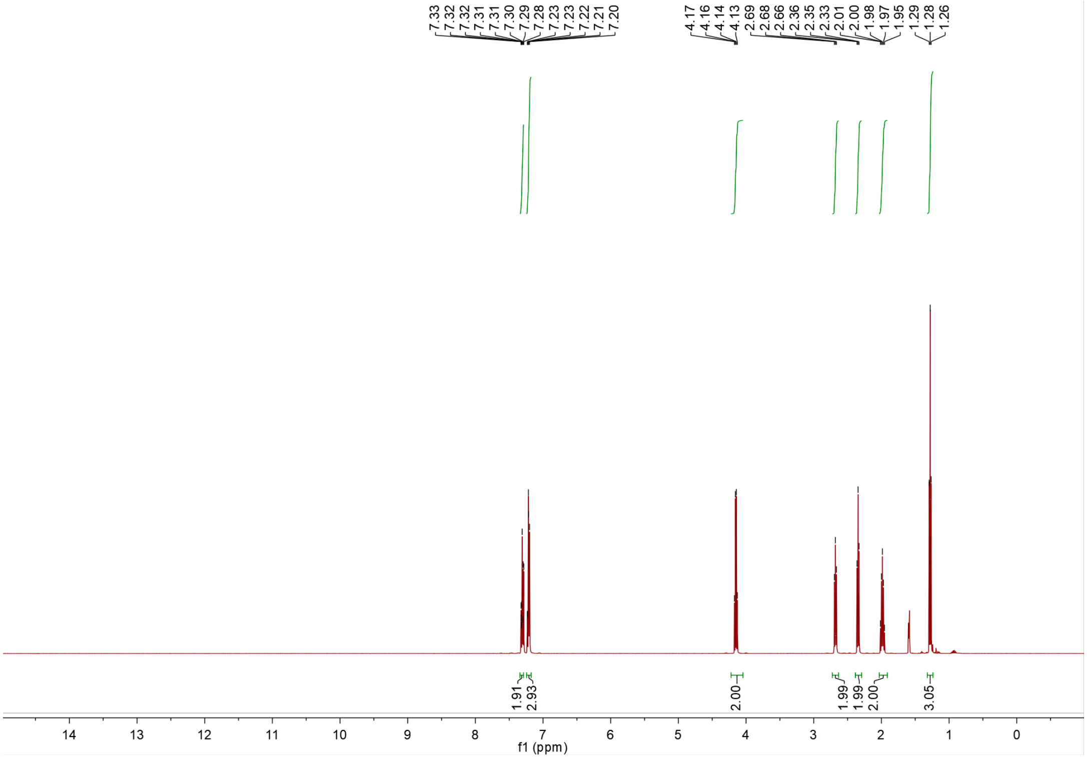
7.33 7.32 7.32 7.31 7.31 7.30 7.29 7.28 7.23 7.23 7.22 7.21 7.20
4.17 4.16 4.14 4.13 2.69 2.68 2.66 2.36 2.35 2.33 2.01 2.00 1.98 1.97 1.95 1.29 1.28 1.26
1.91 2.93 2.00 1.99 1.99 2.00 3.05
f1 (ppm)

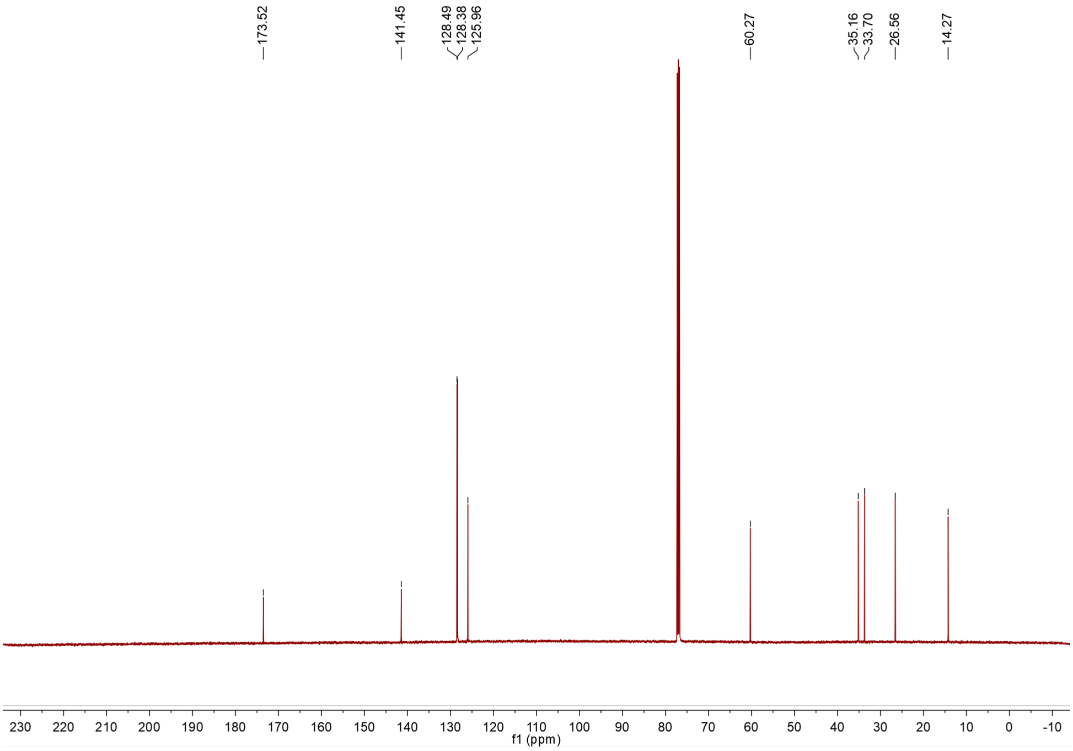
173.52
141.45
128.49 128.38 125.96
60.27
35.16 33.70 26.56 14.27
f1 (ppm)

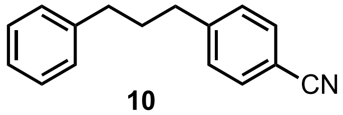
CN

10

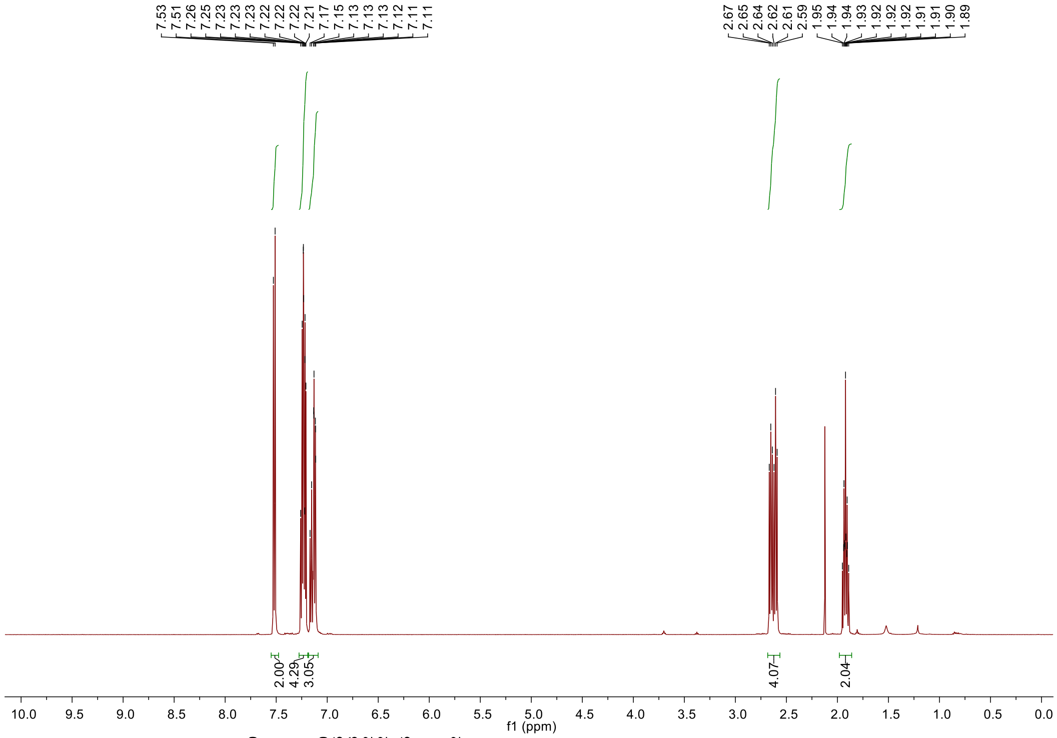
7.53 7.51 7.26 7.25 7.23 7.23 7.23 7.22 7.22 7.22 7.21 7.17 7.15 7.13 7.13 7.13 7.12 7.11 7.11
2.67 2.65 2.64 2.62 2.61 2.59 1.95 1.94 1.94 1.93 1.92 1.92 1.91 1.91 1.90 1.89
2.00
4.29
3.05
4.07
2.04
10.0 9.5 9.0 8.5 8.0 7.5 7.0 6.5 6.0 5.5 5.0 4.5 4.0 3.5 3.0 2.5 2.0 1.5 1.0 0.5 0.0
f1 (ppm)

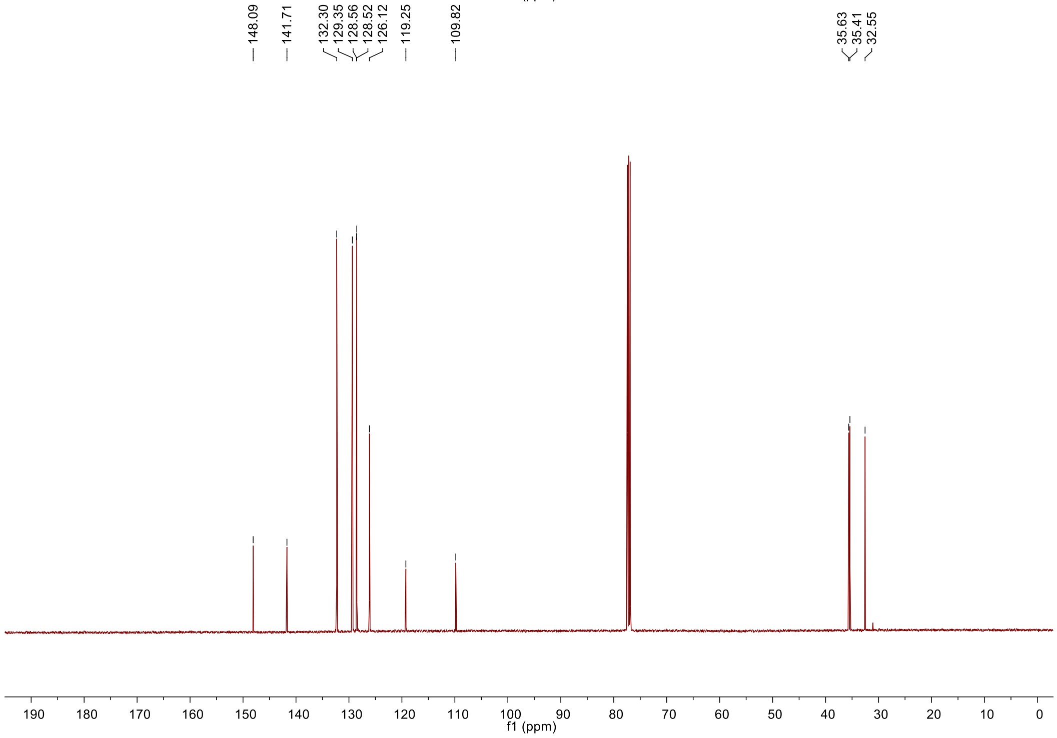
148.09
141.71
132.30 129.35 128.56 128.52 126.12
119.25
109.82
35.63 35.41 32.55
190 180 170 160 150 140 130 120 110 100 90 80 70 60 50 40 30 20 10 0
f1 (ppm)

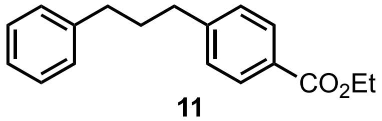

11

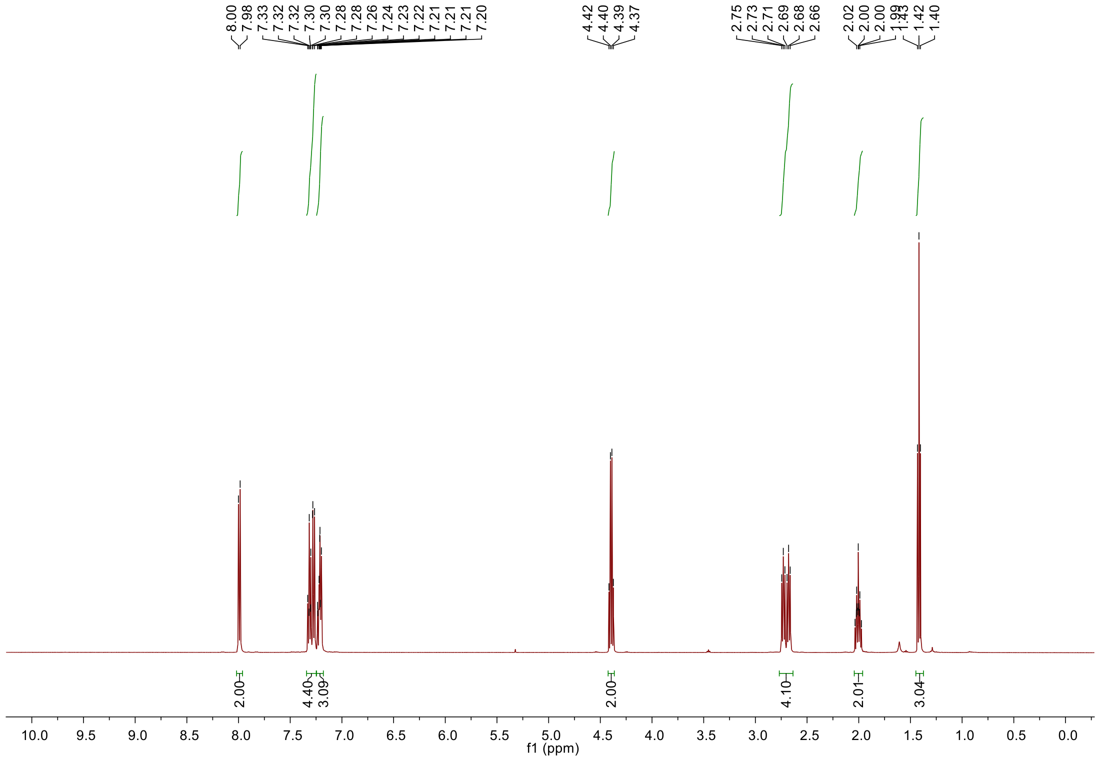

8.00
7.98
7.33
7.32
7.32
7.30
7.30
7.28
7.26
7.24
7.23
7.22
7.21
7.21
7.21
7.20
4.42
4.40
4.39
4.37
2.75
2.73
2.71
2.69
2.68
2.66
2.02
2.00
2.00
1.42
1.40
2.00
4.40
3.09
2.00
4.10
2.01
3.04
f1 (ppm)

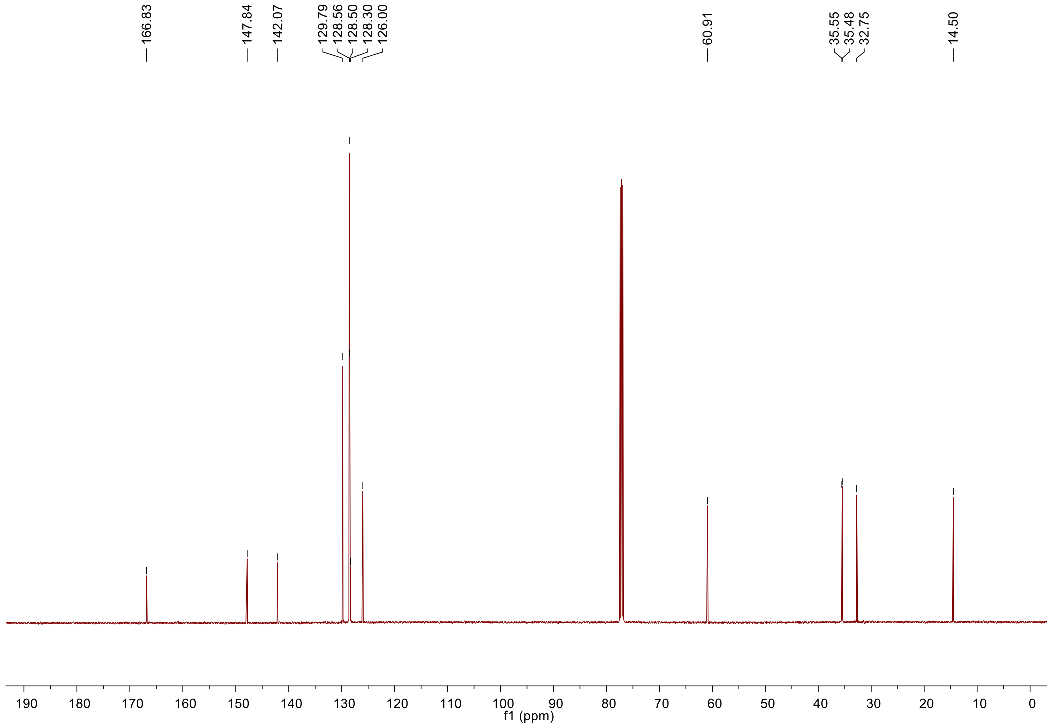

166.83
147.84
142.07
129.79
128.56
128.50
128.30
126.00
60.91
35.55
35.48
32.75
14.50
f1 (ppm)

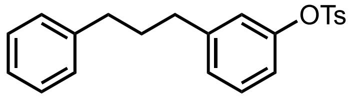
OTs

12

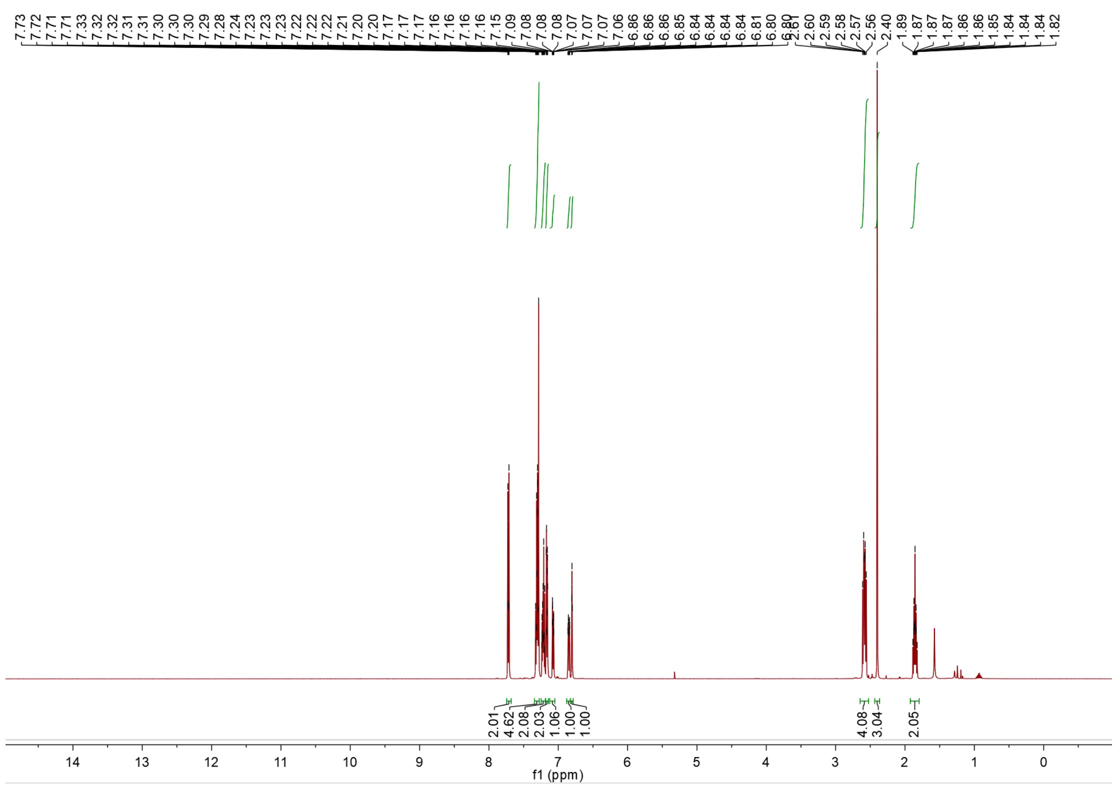
7.73
7.72
7.71
7.71
7.33
7.32
7.32
7.31
7.30
7.30
7.29
7.28
7.24
7.23
7.23
7.23
7.22
7.22
7.22
7.21
7.20
7.20
7.17
7.17
7.16
7.16
7.16
7.15
7.09
7.08
7.08
7.08
7.07
7.07
7.07
7.06
6.86
6.86
6.86
6.85
6.84
6.84
6.84
6.81
6.80
6.80
2.61
2.60
2.59
2.58
2.57
2.56
2.40
1.89
1.87
1.87
1.87
1.86
1.86
1.85
1.84
1.84
1.84
1.82
2.01
4.62
2.08
2.03
1.06
1.00
1.00
4.08
3.04
2.05
14 13 12 11 10 9 8 7 6 5 4 3 2 1 0
f1 (ppm)

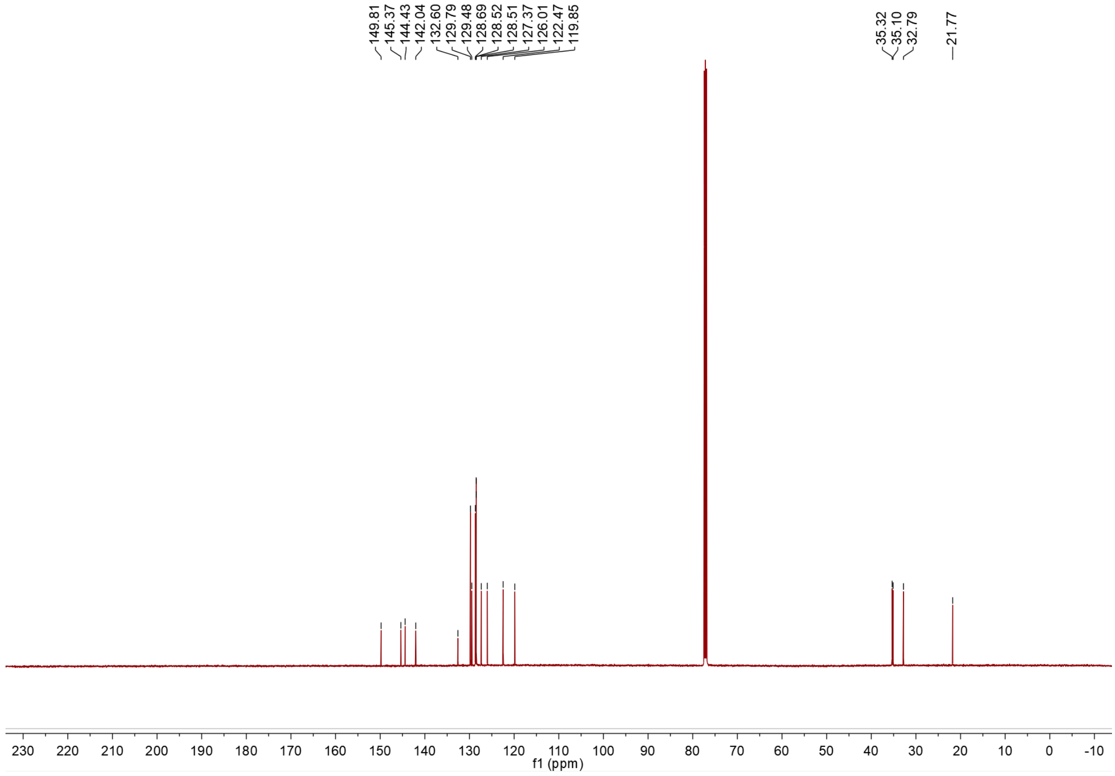
149.81
145.37
144.43
142.04
132.60
129.79
129.48
128.69
128.52
128.51
127.37
126.01
122.47
119.85
35.32
35.10
32.79
21.77
230 220 210 200 190 180 170 160 150 140 130 120 110 100 90 80 70 60 50 40 30 20 10 0 -10
f1 (ppm)

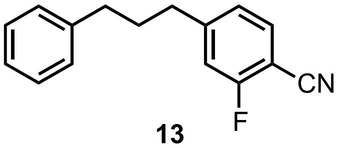

13

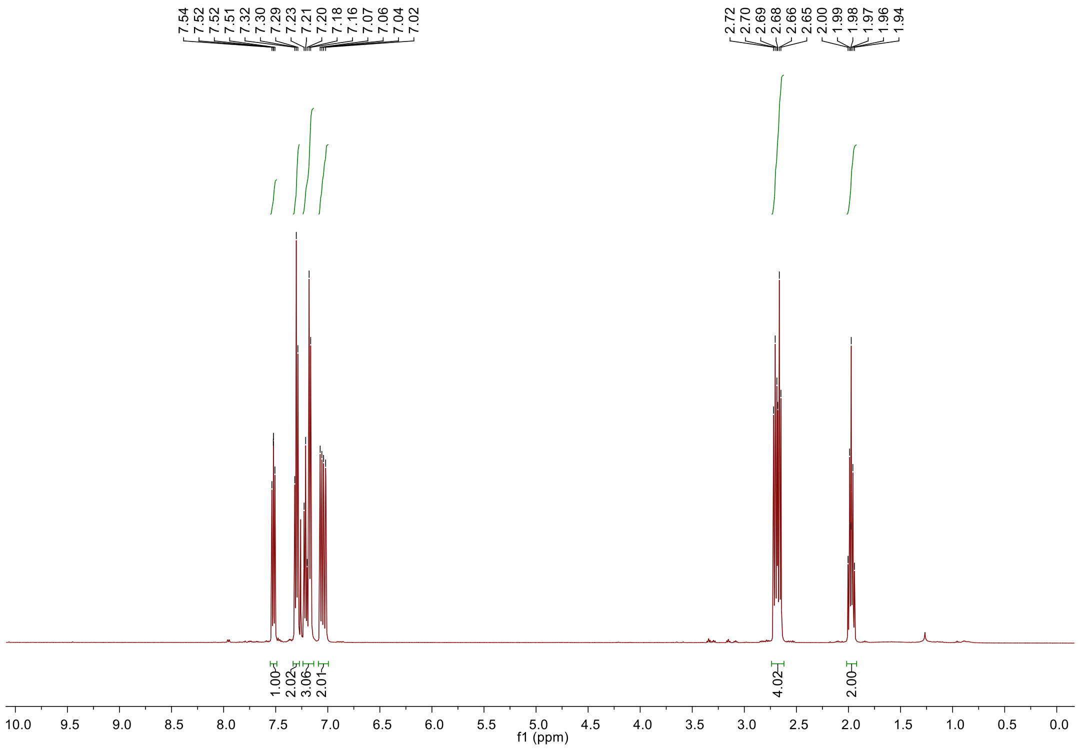

7.54
7.52
7.52
7.51
7.32
7.30
7.29
7.23
7.21
7.20
7.18
7.16
7.07
7.06
7.04
7.02
2.72
2.70
2.69
2.68
2.66
2.65
2.00
1.99
1.98
1.97
1.96
1.94
1.00
2.02
3.06
2.01
4.02
2.00
f1 (ppm)

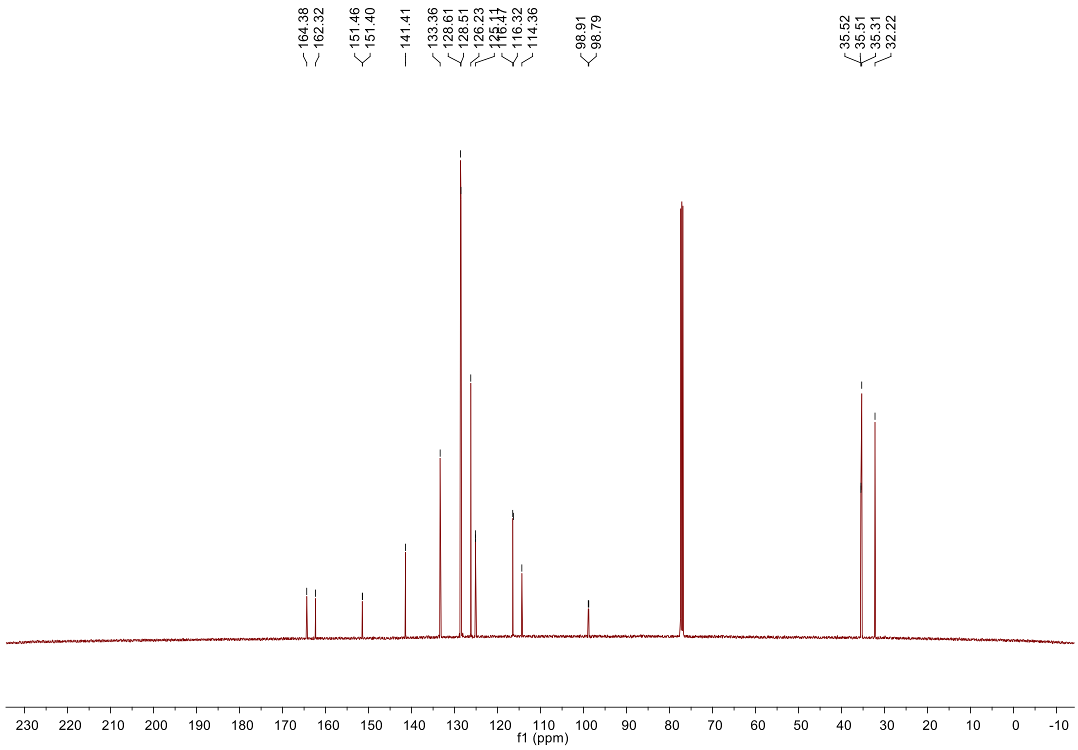

164.38
162.32
151.46
151.40
141.41
133.36
128.61
128.51
126.23
116.32
114.36
98.91
98.79
35.52
35.51
35.31
32.22
f1 (ppm)

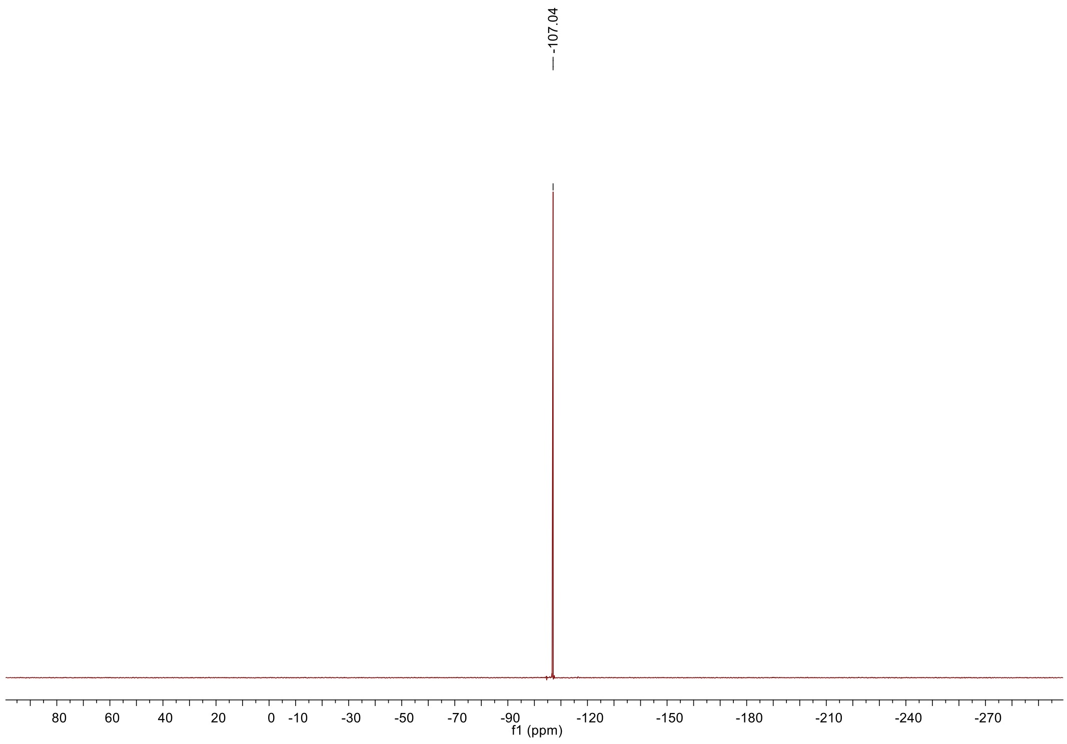
-107.04
80
60
40
20
0
-10
-30
-50
-70
-90
-120
-150
-180
-210
-240
-270
f1 (ppm)

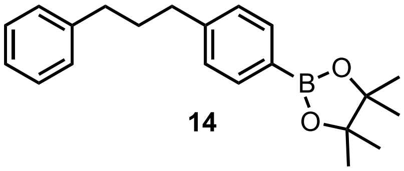

14

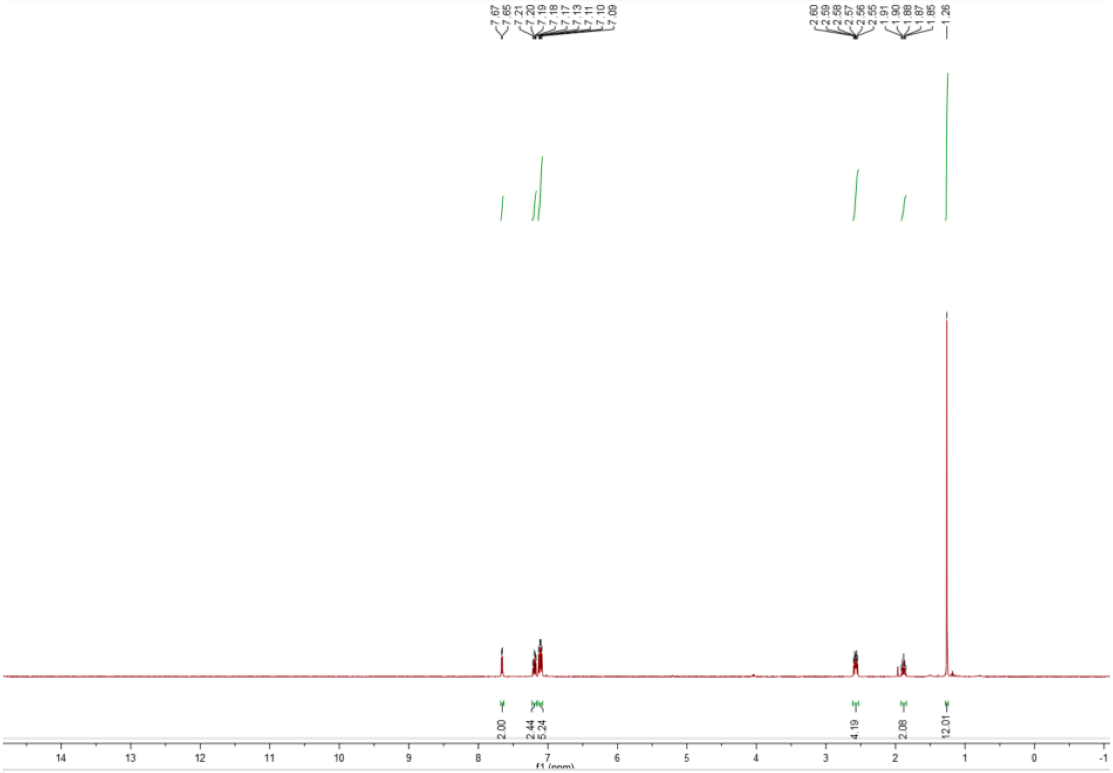
7.67
7.65
7.21
7.20
7.19
7.18
7.17
7.13
7.11
7.10
7.09
2.60
2.59
2.58
2.57
2.56
2.55
1.91
1.90
1.88
1.87
1.85
1.26
2.00
2.44
5.24
4.19
2.08
12.01
14
13
12
11
10
9
8
7
6
5
4
3
2
1
0
-1
f1 (ppm)

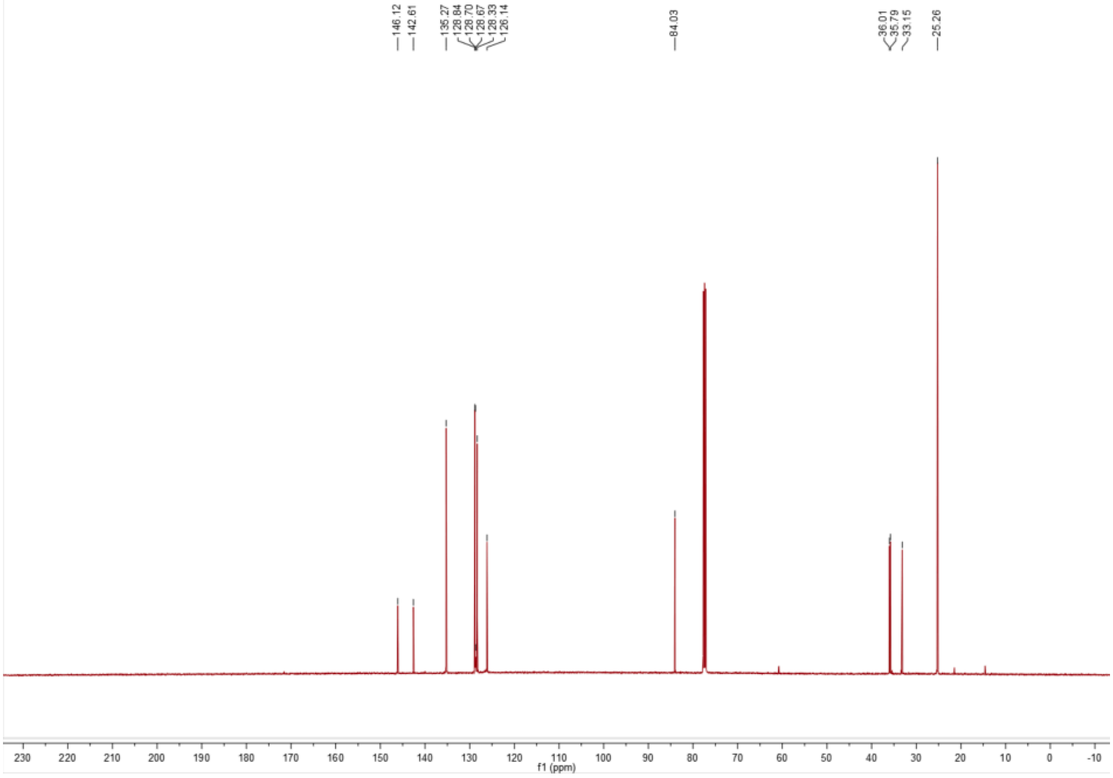
146.12
142.61
135.27
128.84
128.70
128.67
128.33
126.14
84.03
36.01
35.79
33.15
25.26
230
220
210
200
190
180
170
160
150
140
130
120
110
100
90
80
70
60
50
40
30
20
10
0
-10
f1 (ppm)

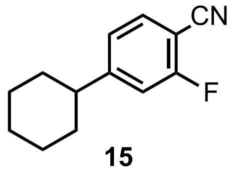
CN
F

15

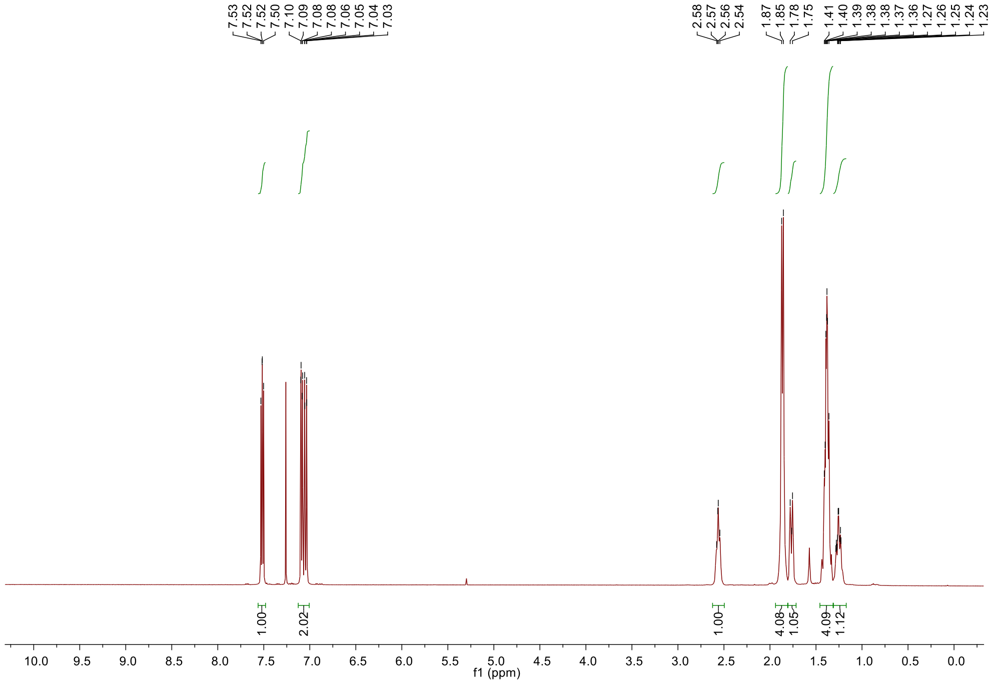
7.53
7.52
7.52
7.50
7.10
7.09
7.08
7.08
7.06
7.05
7.04
7.03
2.58
2.57
2.56
2.54
1.87
1.85
1.78
1.75
1.41
1.40
1.39
1.38
1.38
1.37
1.36
1.27
1.26
1.25
1.24
1.23
1.00
2.02
1.00
4.08
1.05
4.09
1.12
f1 (ppm)

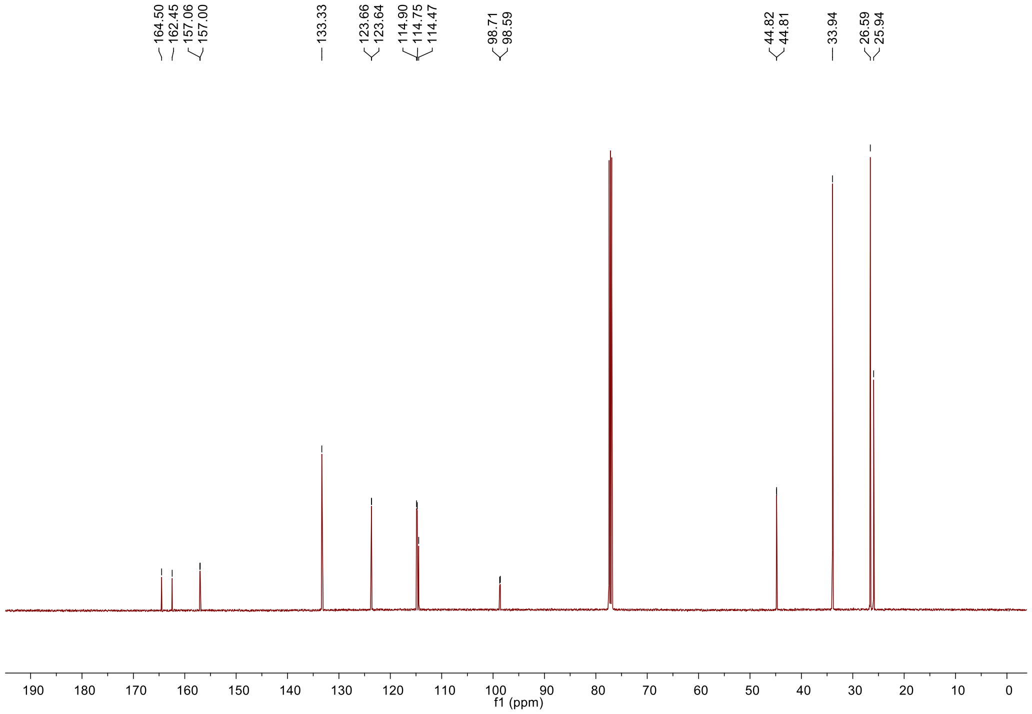
164.50
162.45
157.06
157.00
133.33
123.66
123.64
114.90
114.75
114.47
98.71
98.59
44.82
44.81
33.94
26.59
25.94
f1 (ppm)

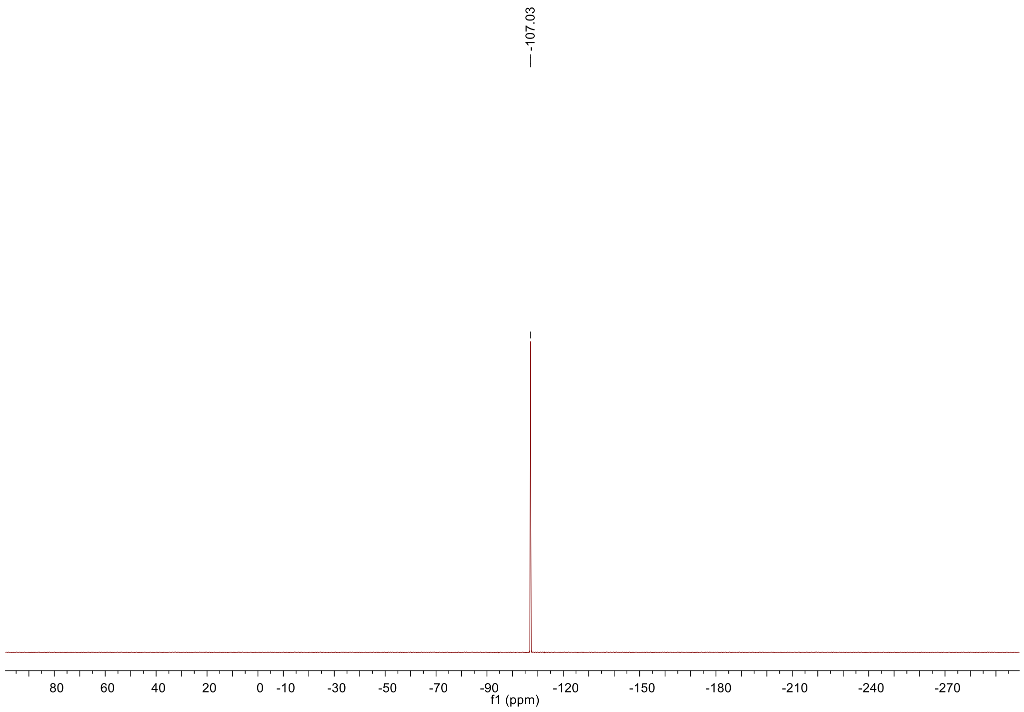
-107.03
80
60
40
20
0
-10
-30
-50
-70
-90
-120
-150
-180
-210
-240
-270
f1 (ppm)

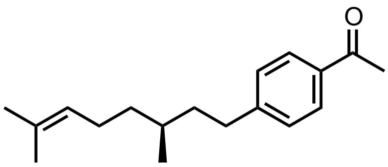
O

16

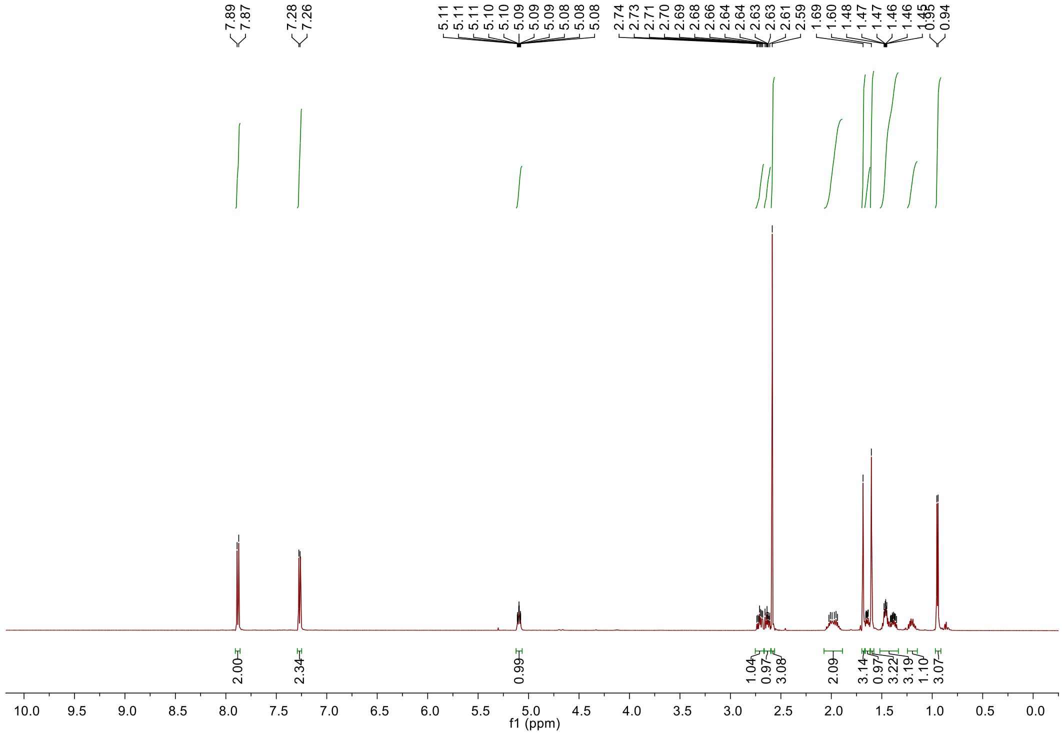
7.89
7.87
7.28
7.26
5.11
5.11
5.11
5.10
5.09
5.09
5.09
5.08
5.08
5.08
2.74
2.73
2.71
2.70
2.69
2.68
2.66
2.64
2.64
2.63
2.63
2.61
2.59
1.69
1.60
1.48
1.47
1.47
1.46
1.46
1.45
0.95
0.94
2.00
2.34
0.99
1.04
0.97
3.08
2.09
3.14
0.97
3.22
3.19
1.10
3.07
10.0 9.5 9.0 8.5 8.0 7.5 7.0 6.5 6.0 5.5 5.0 4.5 4.0 3.5 3.0 2.5 2.0 1.5 1.0 0.5 0.0
f1 (ppm)

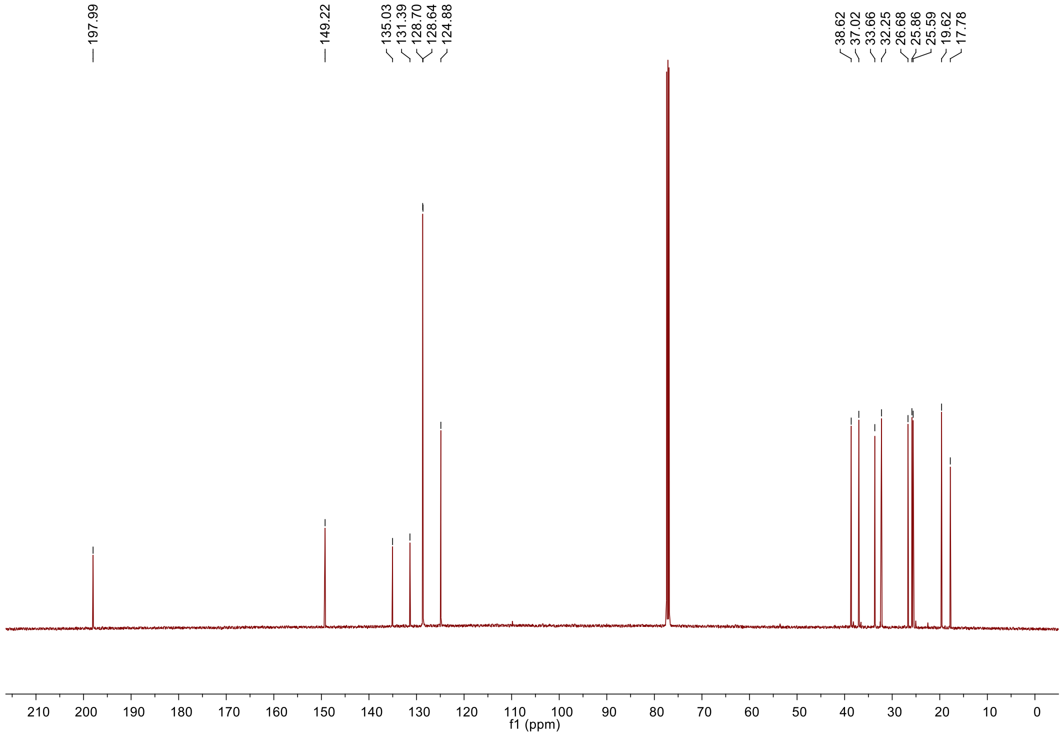
197.99
149.22
135.03
131.39
128.70
128.64
124.88
38.62
37.02
33.66
32.25
26.68
25.86
25.59
19.62
17.78
210 200 190 180 170 160 150 140 130 120 110 100 90 80 70 60 50 40 30 20 10 0
f1 (ppm)

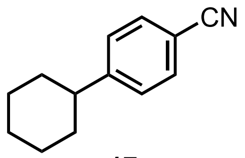
CN

17

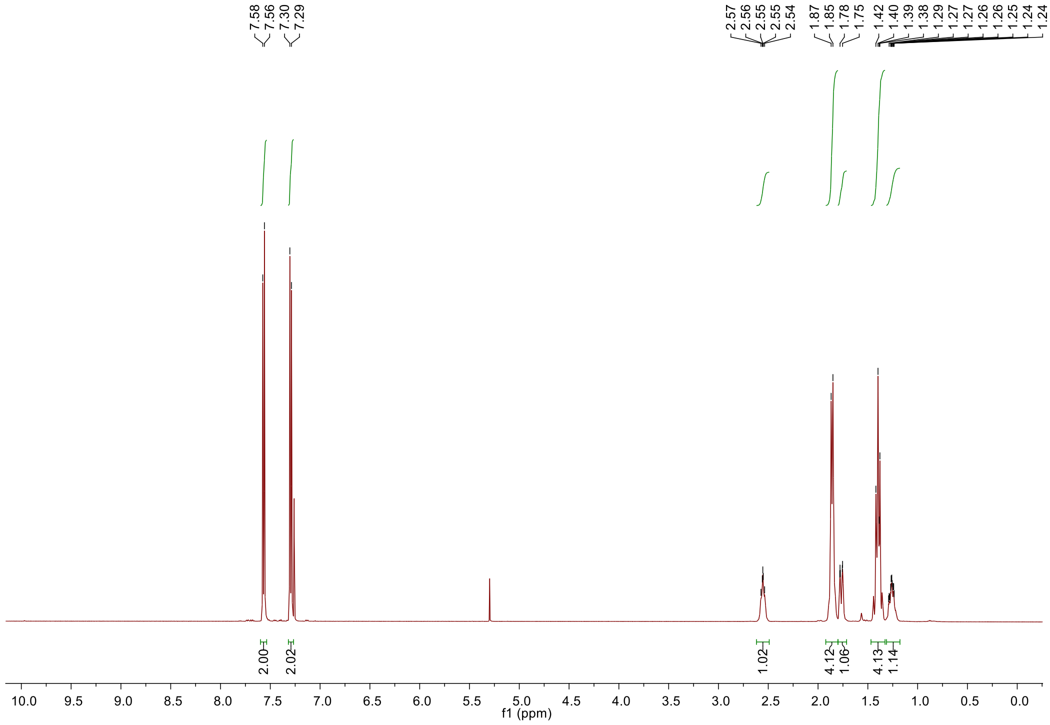
7.58
7.56
7.30
7.29
2.57
2.56
2.55
2.55
2.54
1.87
1.85
1.78
1.75
1.42
1.40
1.39
1.38
1.29
1.27
1.27
1.26
1.25
1.24
1.24
2.00
2.02
1.02
4.12
1.06
4.13
1.14
10.0 9.5 9.0 8.5 8.0 7.5 7.0 6.5 6.0 5.5 5.0 4.5 4.0 3.5 3.0 2.5 2.0 1.5 1.0 0.5 0.0
f1 (ppm)

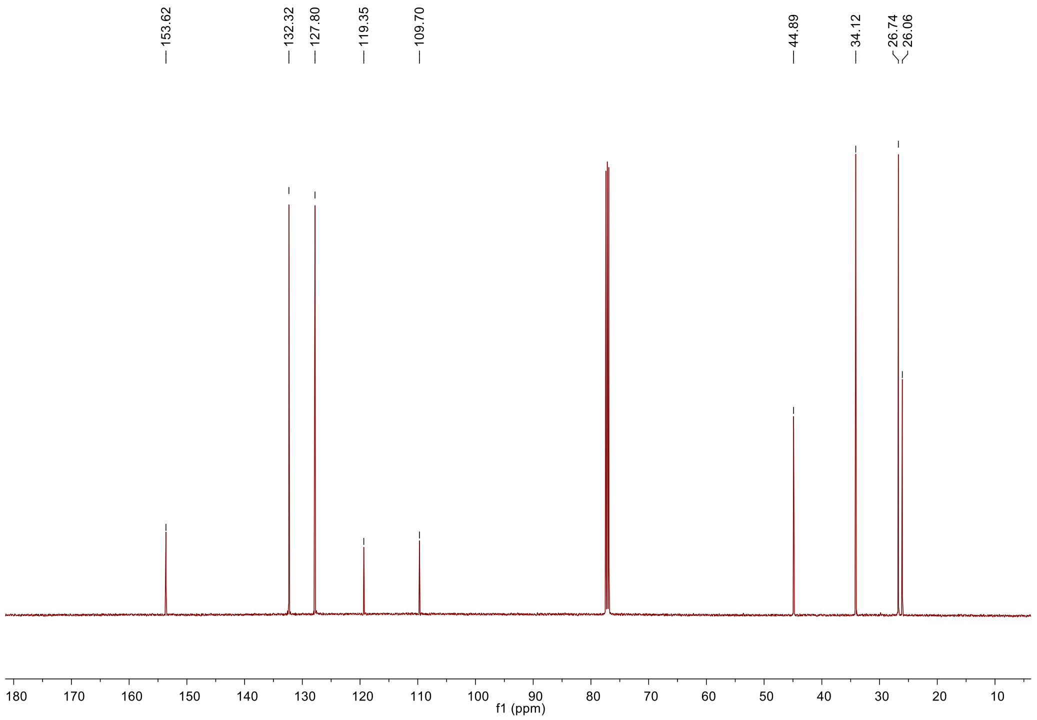
153.62
132.32
127.80
119.35
109.70
44.89
34.12
26.74
26.06
180 170 160 150 140 130 120 110 100 90 80 70 60 50 40 30 20 10
f1 (ppm)

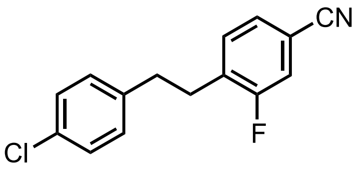

18

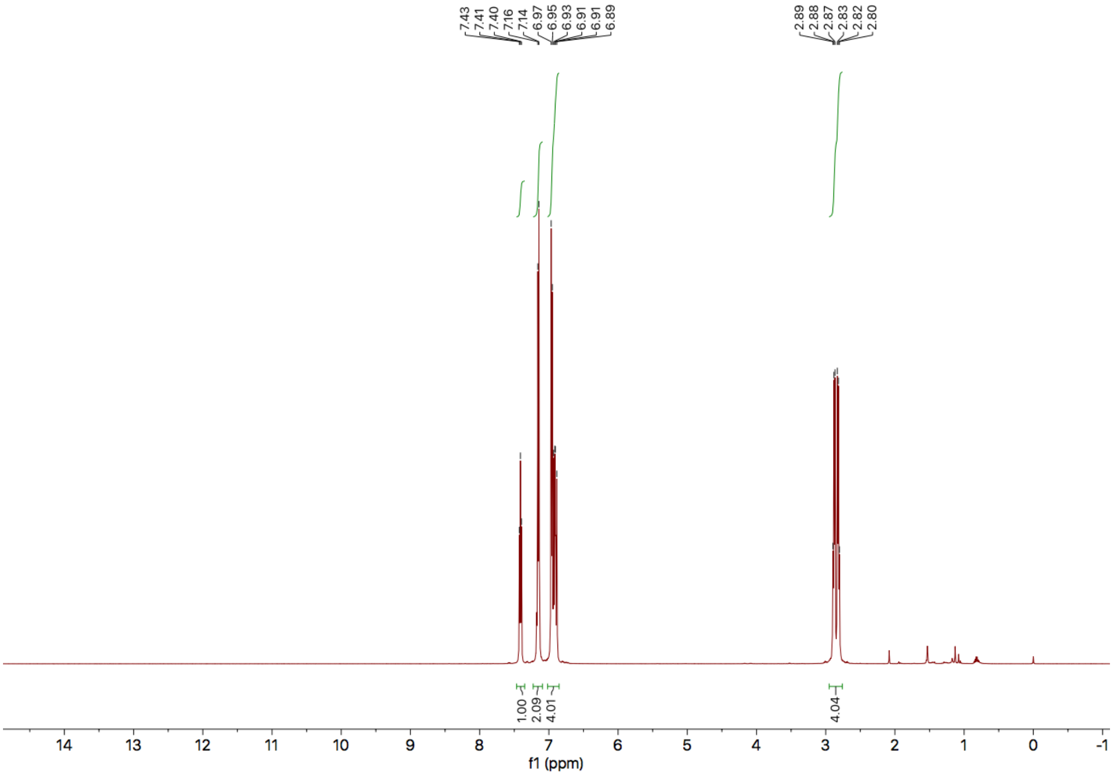
7.43
7.41
7.40
7.16
7.14
6.97
6.95
6.93
6.91
6.91
6.89
2.89
2.88
2.87
2.83
2.82
2.80
1.00
2.09
4.01
4.04
f1 (ppm)

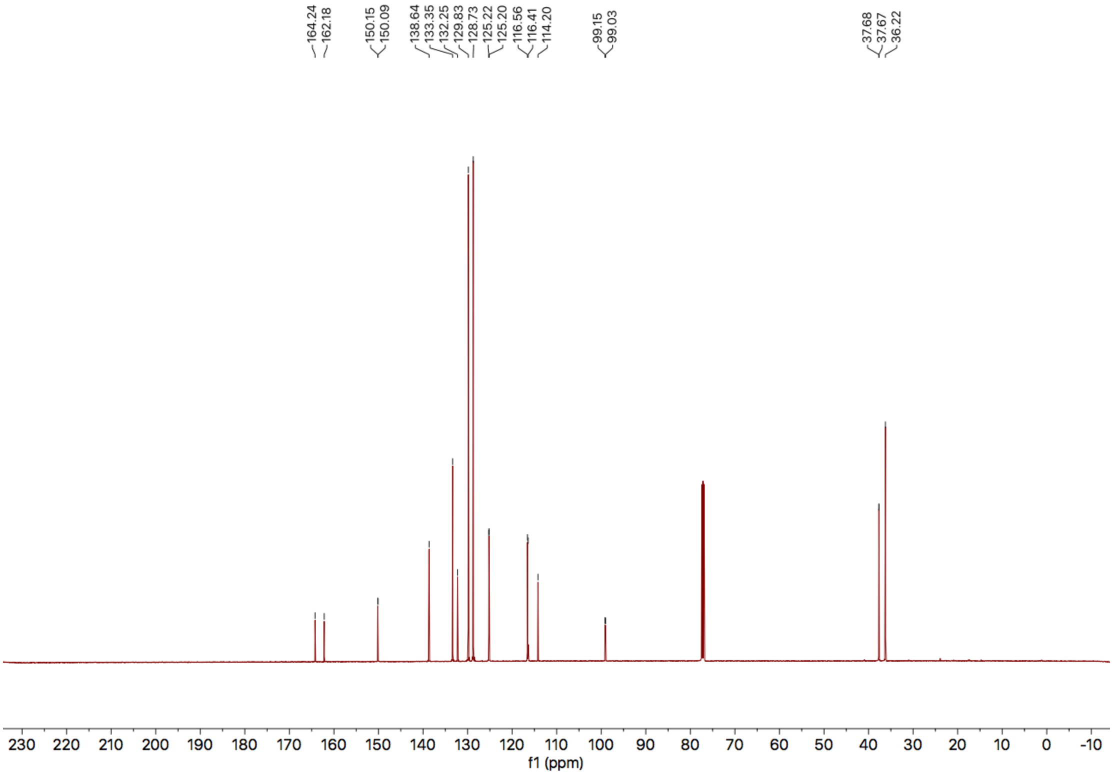
164.24
162.18
150.15
150.09
138.64
133.35
132.25
129.83
128.73
125.22
125.20
116.56
116.41
114.20
99.15
99.03
37.68
37.67
36.22
f1 (ppm)

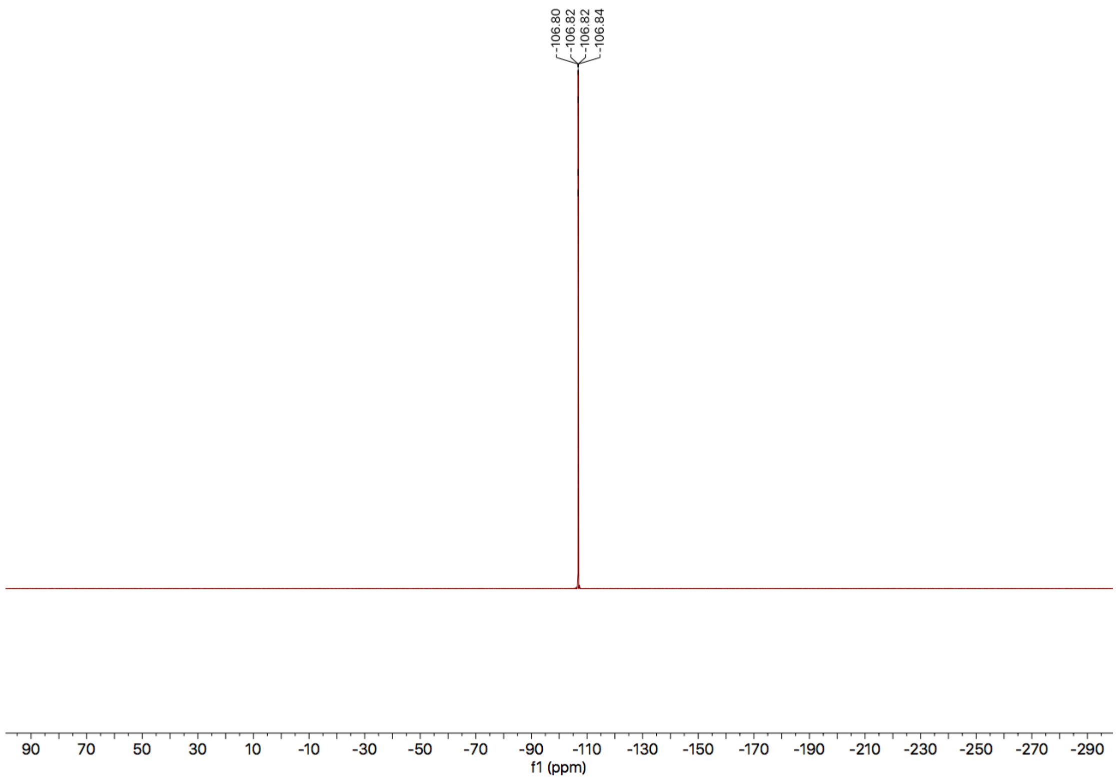
-106.80
-106.82
-106.82
-106.84
90 70 50 30 10 -10 -30 -50 -70 -90 -110 -130 -150 -170 -190 -210 -230 -250 -270 -290
f1 (ppm)

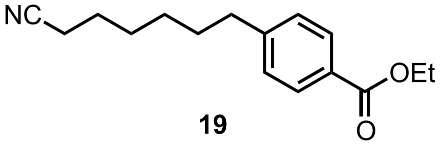
NC
OEt
O

19

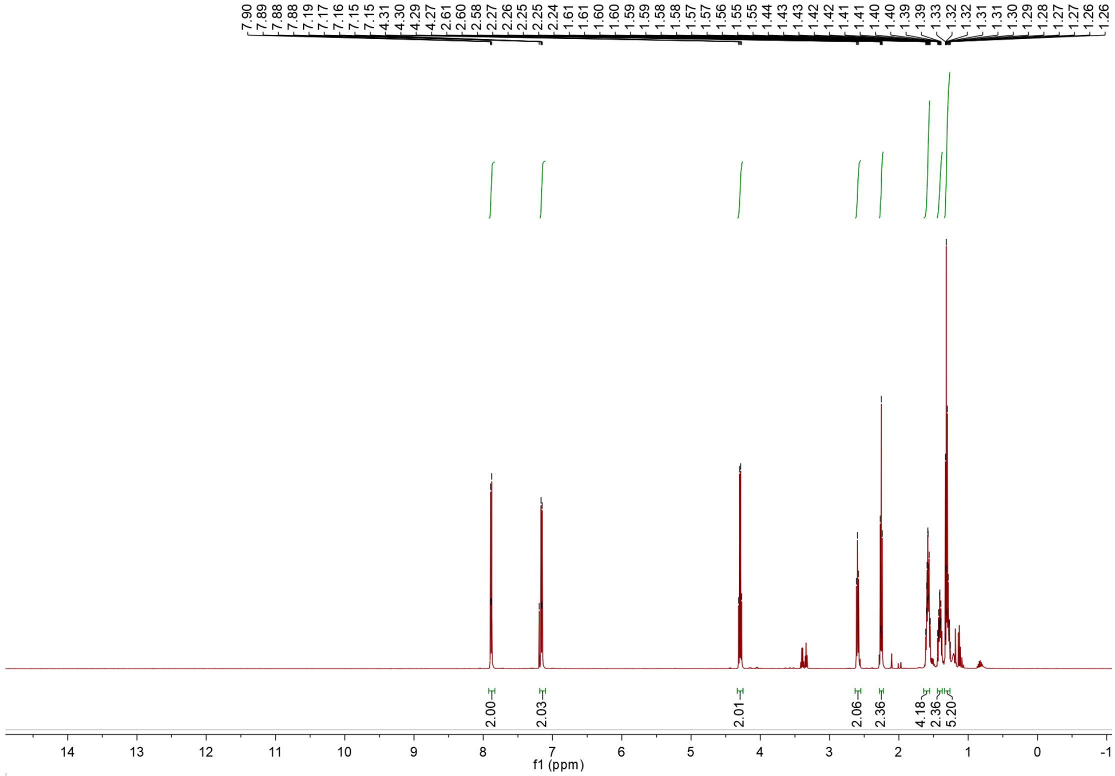
7.90
7.89
7.88
7.88
7.19
7.17
7.16
7.15
7.15
4.31
4.30
4.29
4.27
2.61
2.60
2.58
2.27
2.26
2.25
2.25
2.24
1.61
1.61
1.60
1.60
1.59
1.59
1.58
1.58
1.57
1.57
1.56
1.55
1.55
1.44
1.43
1.43
1.42
1.42
1.41
1.41
1.40
1.40
1.39
1.39
1.33
1.32
1.32
1.31
1.31
1.30
1.29
1.28
1.27
1.27
1.26
1.26
2.00
2.03
2.01
2.06
2.36
4.18
2.36
5.20
f1 (ppm)

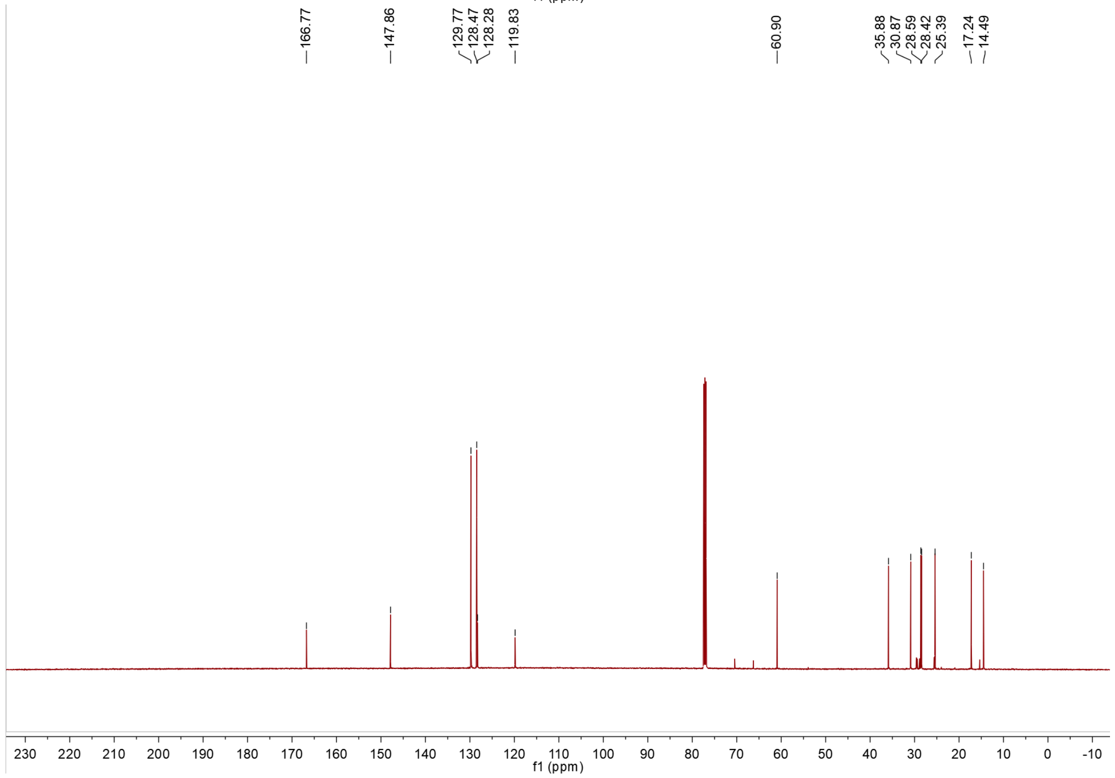
166.77
147.86
129.77
128.47
128.28
119.83
60.90
35.88
30.87
28.59
28.42
25.39
17.24
14.49
f1 (ppm)

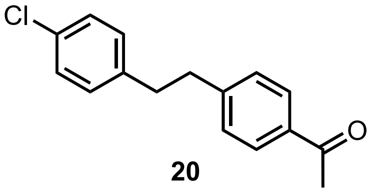
Cl
O

20

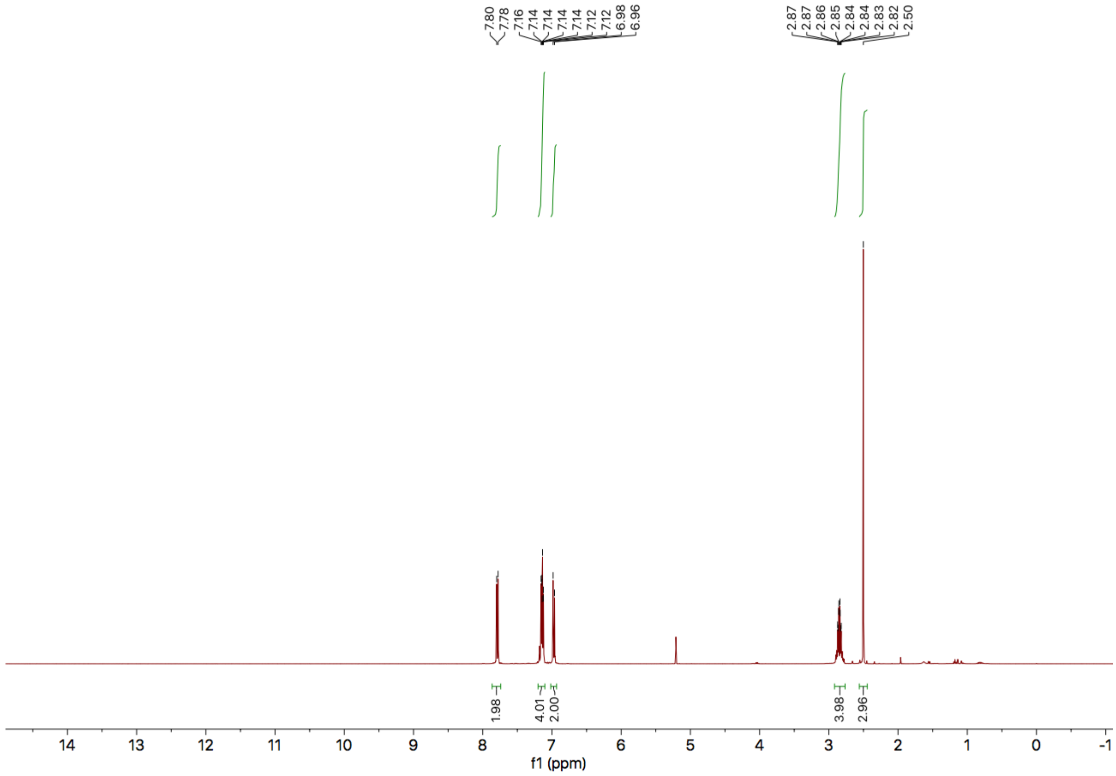
7.80
7.78
7.16
7.14
7.14
7.14
7.14
7.12
6.98
6.96
2.87
2.87
2.86
2.85
2.84
2.84
2.83
2.82
2.50
1.98
4.01
2.00
3.98
2.96
14 13 12 11 10 9 8 7 6 5 4 3 2 1 0 -1
f1 (ppm)

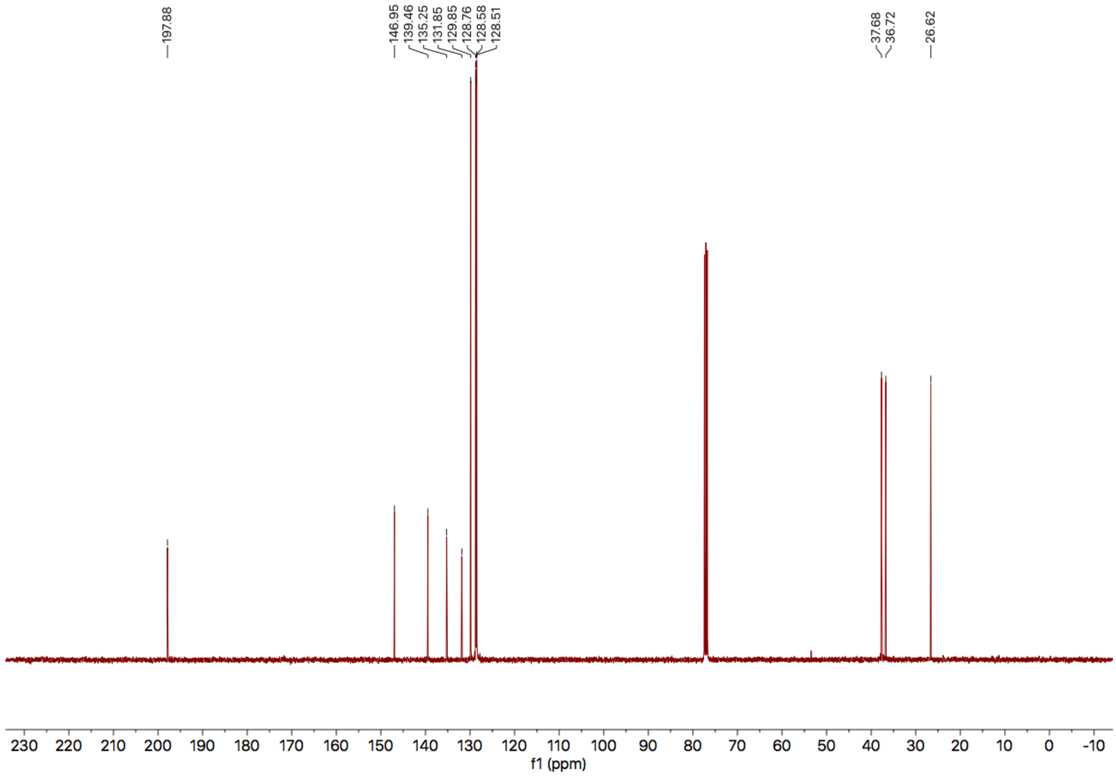
197.88
146.95
139.46
135.25
131.85
129.85
128.76
128.58
128.51
37.68
36.72
26.62
230 220 210 200 190 180 170 160 150 140 130 120 110 100 90 80 70 60 50 40 30 20 10 0 -10
f1 (ppm)

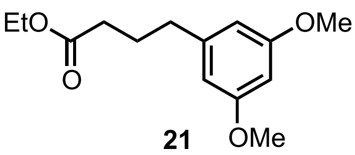
EtO
OMe
O
OMe

21

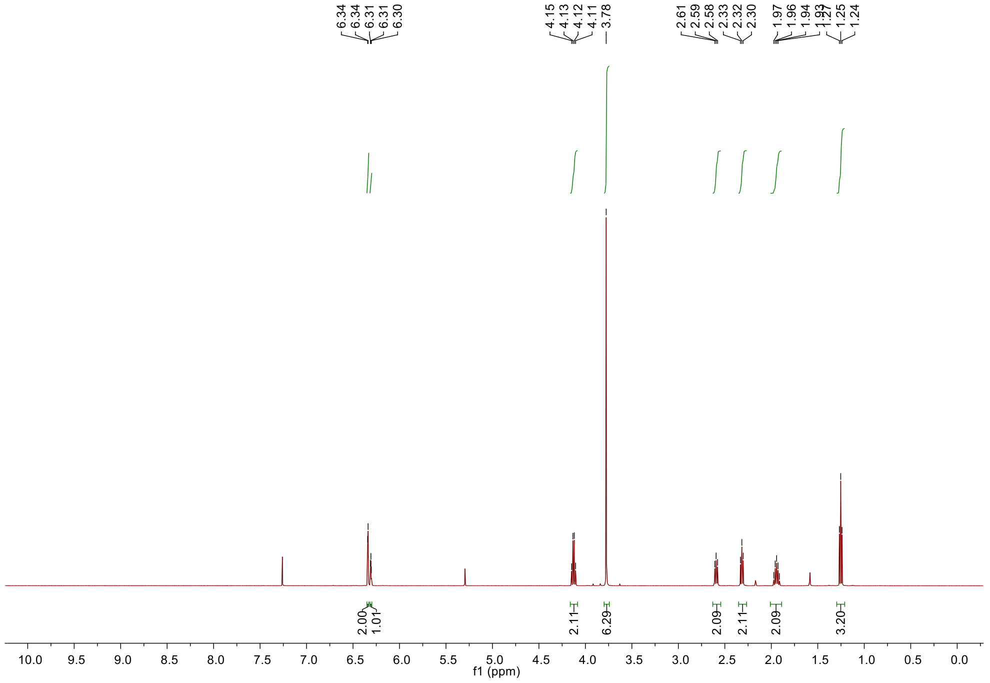
6.34
6.34
6.31
6.31
6.30
4.15
4.13
4.12
4.11
3.78
2.61
2.59
2.58
2.33
2.32
2.30
1.97
1.96
1.94
1.93
1.27
1.25
1.24
2.00
1.01
2.11
6.29
2.09
2.11
2.09
3.20
f1 (ppm)

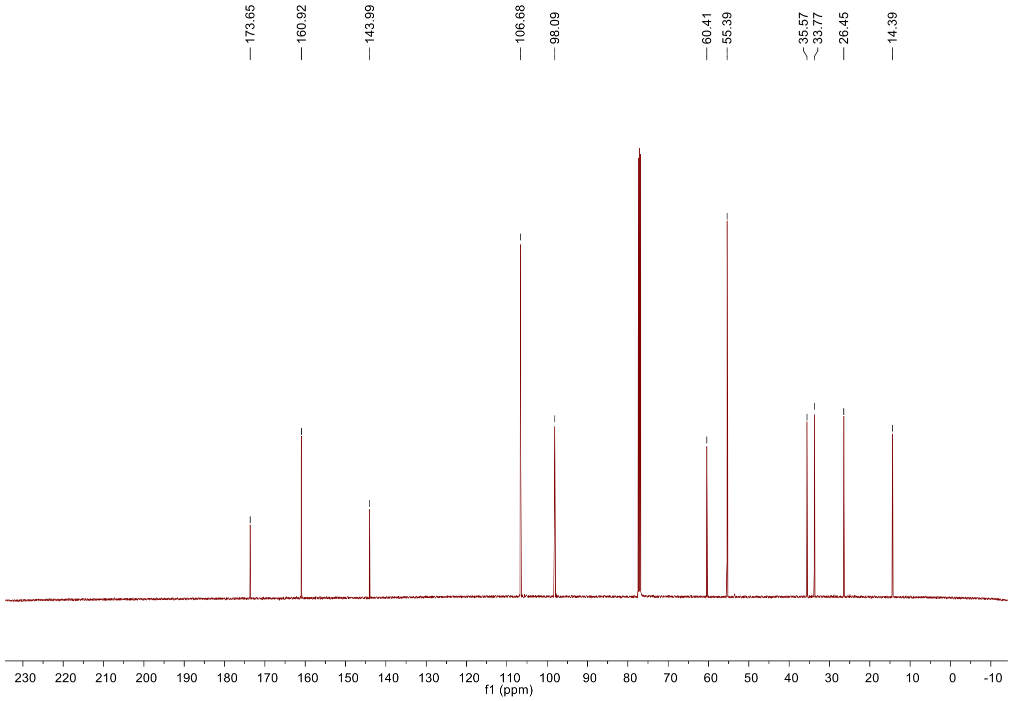
173.65
160.92
143.99
106.68
98.09
60.41
55.39
35.57
33.77
26.45
14.39
f1 (ppm)

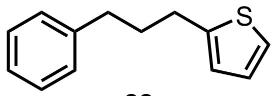
S

22

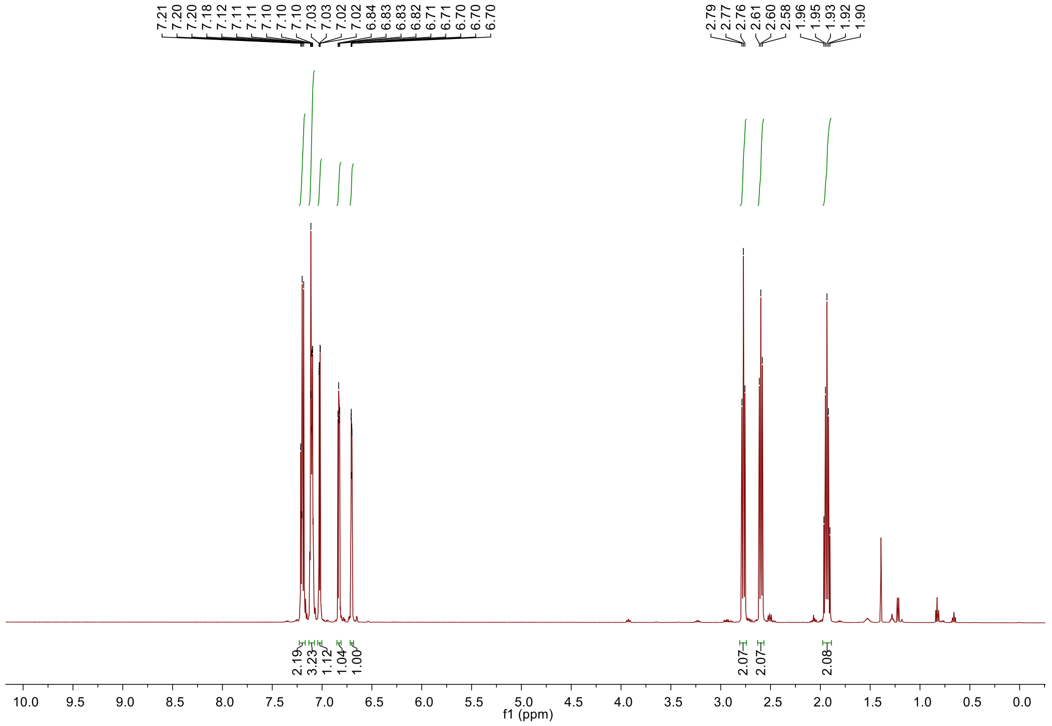
7.21
7.20
7.20
7.18
7.12
7.11
7.11
7.10
7.10
7.10
7.03
7.03
7.02
7.02
6.84
6.83
6.83
6.82
6.71
6.71
6.70
6.70
6.70
2.79
2.77
2.76
2.61
2.60
2.58
1.96
1.95
1.93
1.92
1.90
2.19
3.23
1.12
1.04
1.00
2.07
2.07
2.08
10.0 9.5 9.0 8.5 8.0 7.5 7.0 6.5 6.0 5.5 5.0 4.5 4.0 3.5 3.0 2.5 2.0 1.5 1.0 0.5 0.0
f1 (ppm)

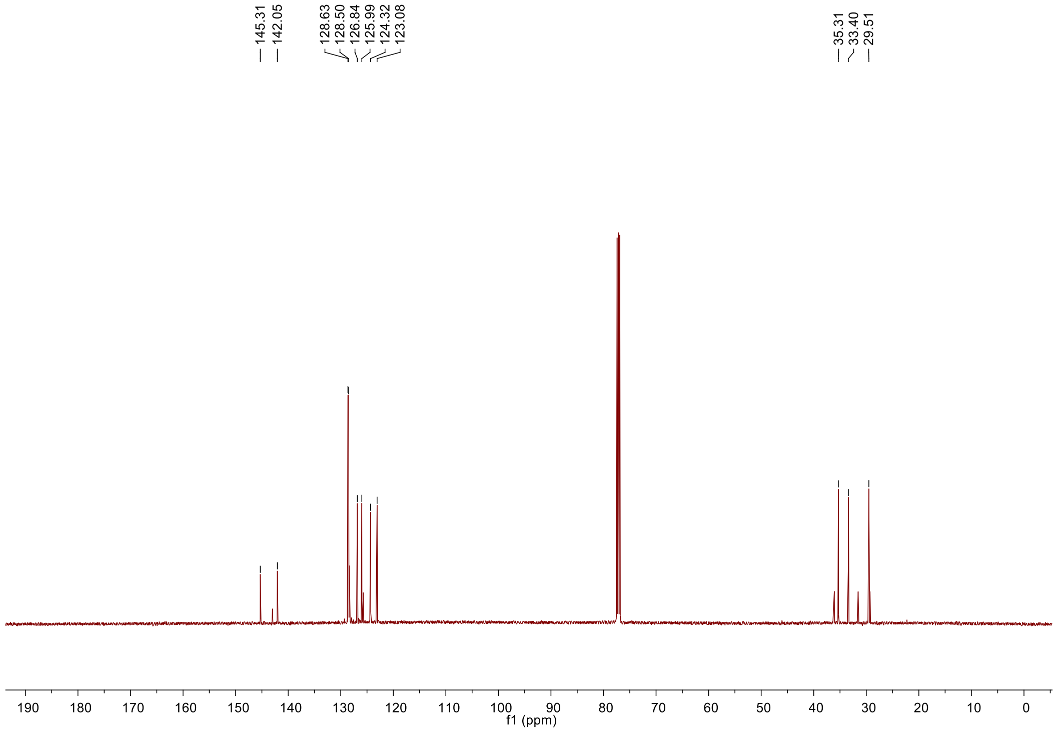
145.31
142.05
128.63
128.50
126.84
125.99
124.32
123.08
35.31
33.40
29.51
190 180 170 160 150 140 130 120 110 100 90 80 70 60 50 40 30 20 10 0
f1 (ppm)

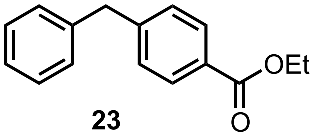
OEt
O

23

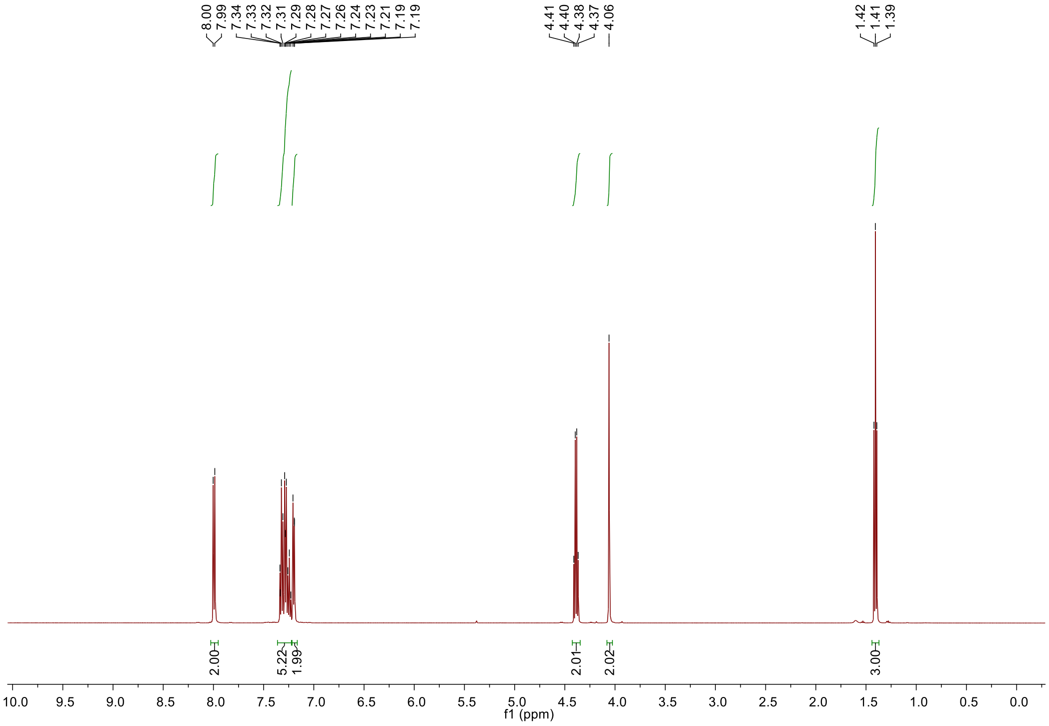
8.00
7.99
7.34
7.33
7.32
7.31
7.29
7.28
7.27
7.26
7.24
7.23
7.21
7.19
7.19
4.41
4.40
4.38
4.37
4.06
1.42
1.41
1.39
2.00
5.22
1.99
2.01
2.02
3.00
f1 (ppm)

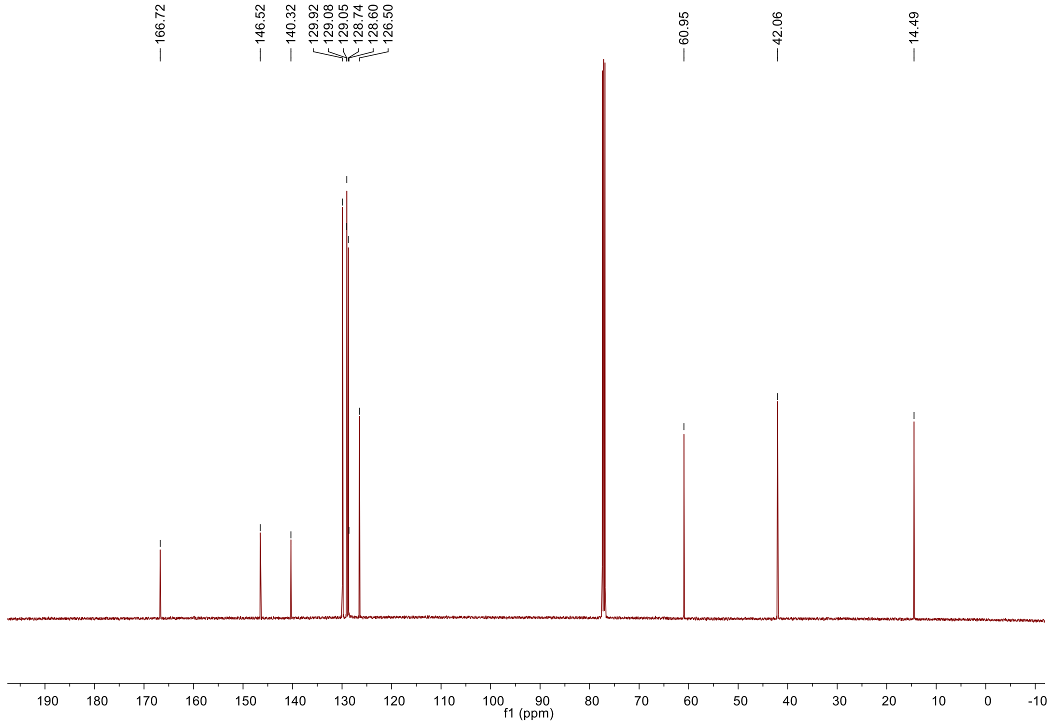
166.72
146.52
140.32
129.92
129.08
129.05
128.74
128.60
126.50
60.95
42.06
14.49
f1 (ppm)

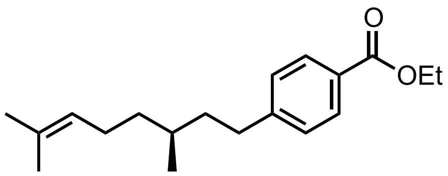
O
OEt

24

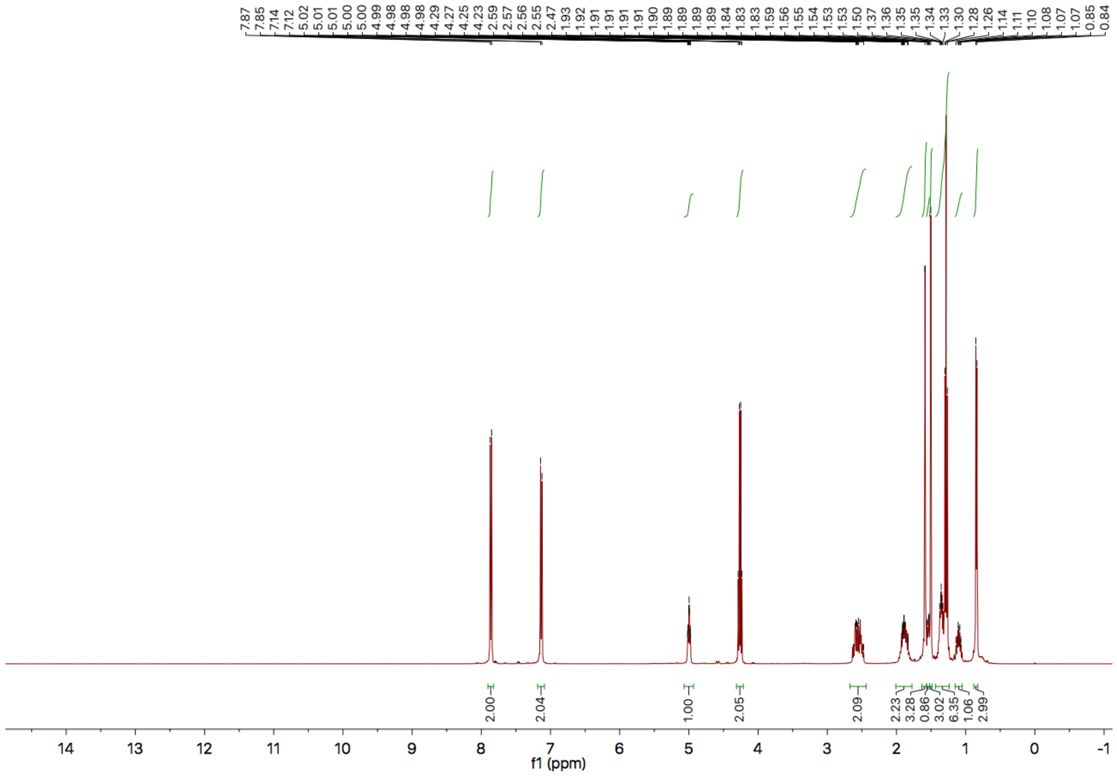
7.87
7.85
7.14
7.12
5.02
5.01
5.01
5.00
5.00
4.99
4.98
4.98
4.98
4.29
4.27
4.25
4.23
2.59
2.57
2.56
2.55
2.47
1.93
1.92
1.91
1.91
1.91
1.91
1.90
1.89
1.89
1.89
1.89
1.84
1.83
1.83
1.59
1.56
1.55
1.54
1.53
1.53
1.50
1.37
1.36
1.35
1.35
1.34
1.33
1.30
1.28
1.26
1.14
1.11
1.10
1.08
1.07
0.85
0.84
2.00
2.04
1.00
2.05
2.09
2.23
3.28
0.86
3.02
6.35
1.06
2.99
14 13 12 11 10 9 8 7 6 5 4 3 2 1 0 -1
f1 (ppm)

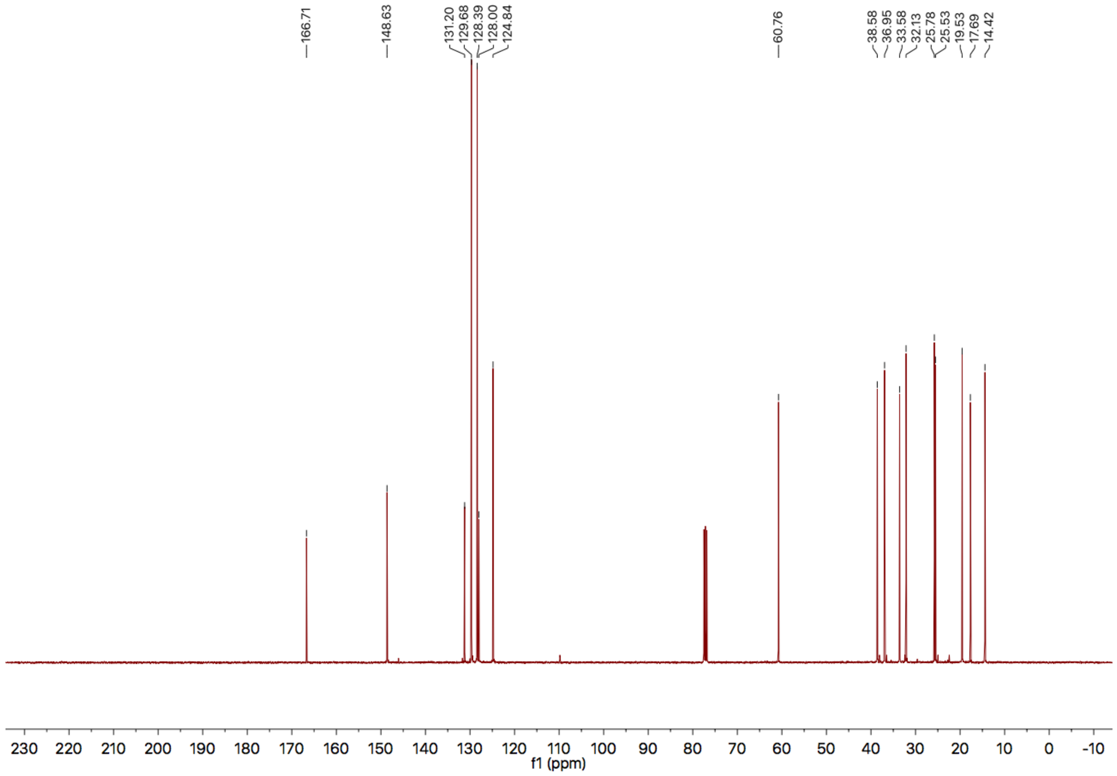
166.71
148.63
131.20
129.68
128.39
128.00
124.84
60.76
38.58
36.95
33.58
32.13
25.78
25.53
19.53
17.69
14.42
230 220 210 200 190 180 170 160 150 140 130 120 110 100 90 80 70 60 50 40 30 20 10 0 -10
f1 (ppm)

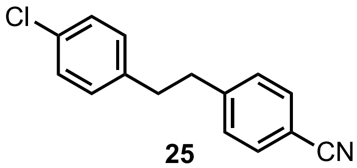
Cl
CN

25

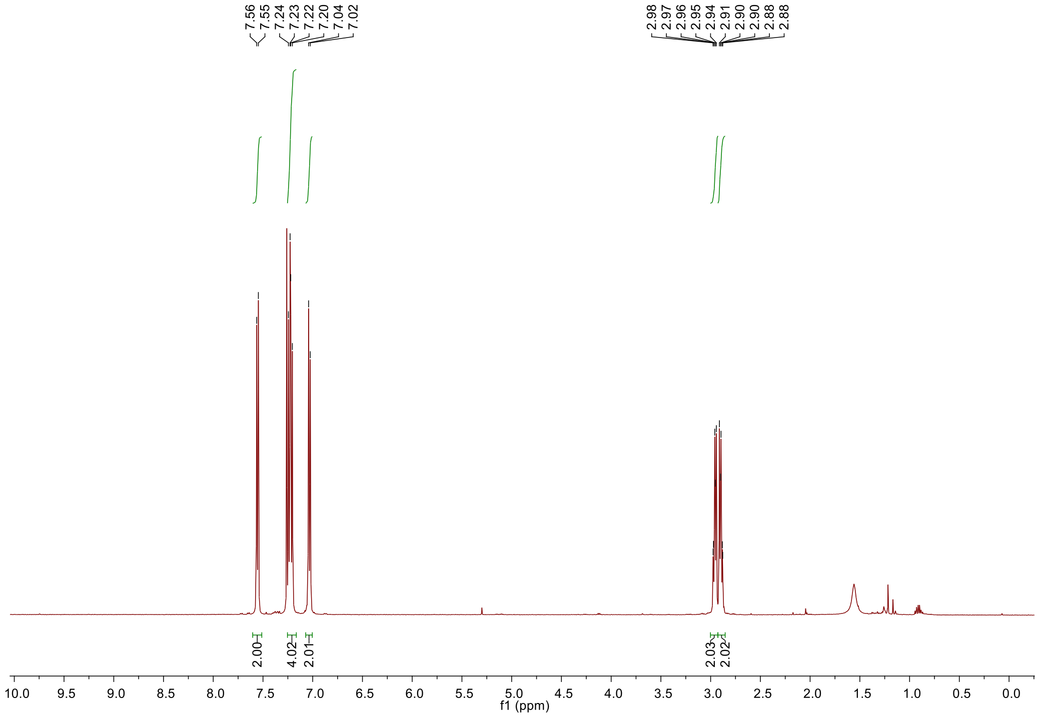
7.56
7.55
7.24
7.23
7.22
7.20
7.04
7.02
2.98
2.97
2.96
2.95
2.94
2.91
2.90
2.90
2.88
2.88
2.00
4.02
2.01
2.03
2.02
10.0
9.5
9.0
8.5
8.0
7.5
7.0
6.5
6.0
5.5
5.0
4.5
4.0
3.5
3.0
2.5
2.0
1.5
1.0
0.5
0.0
f1 (ppm)

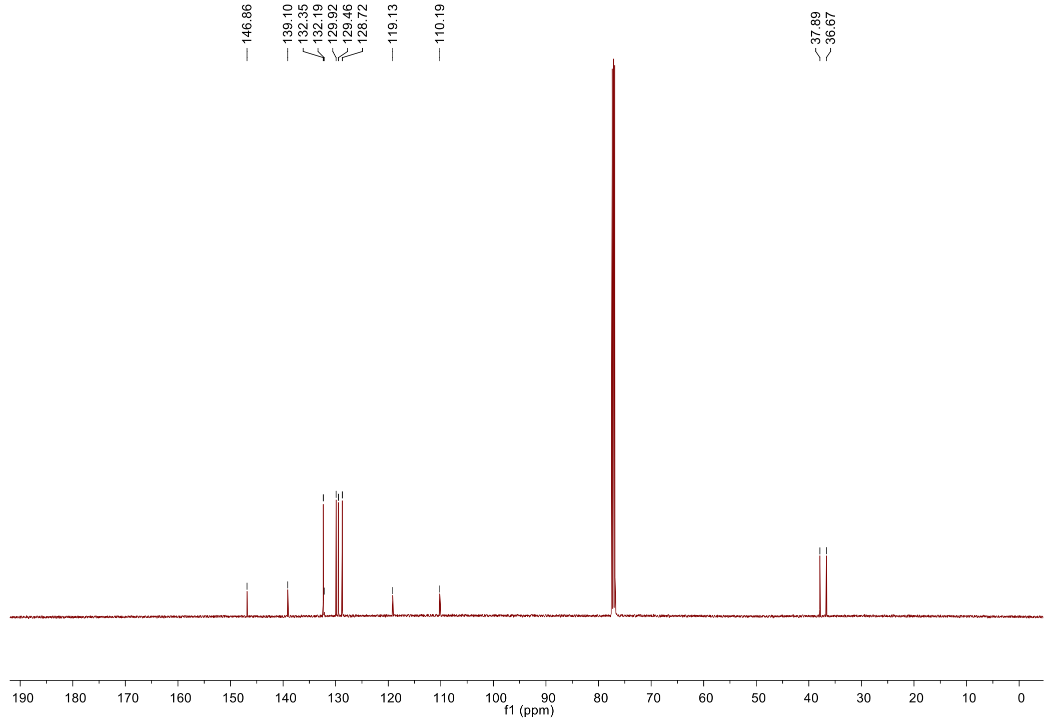
146.86
139.10
132.35
132.19
129.92
129.46
128.72
119.13
110.19
37.89
36.67
190
180
170
160
150
140
130
120
110
100
90
80
70
60
50
40
30
20
10
0
f1 (ppm)

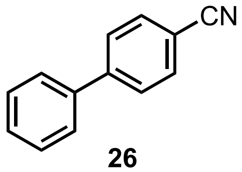
CN

26

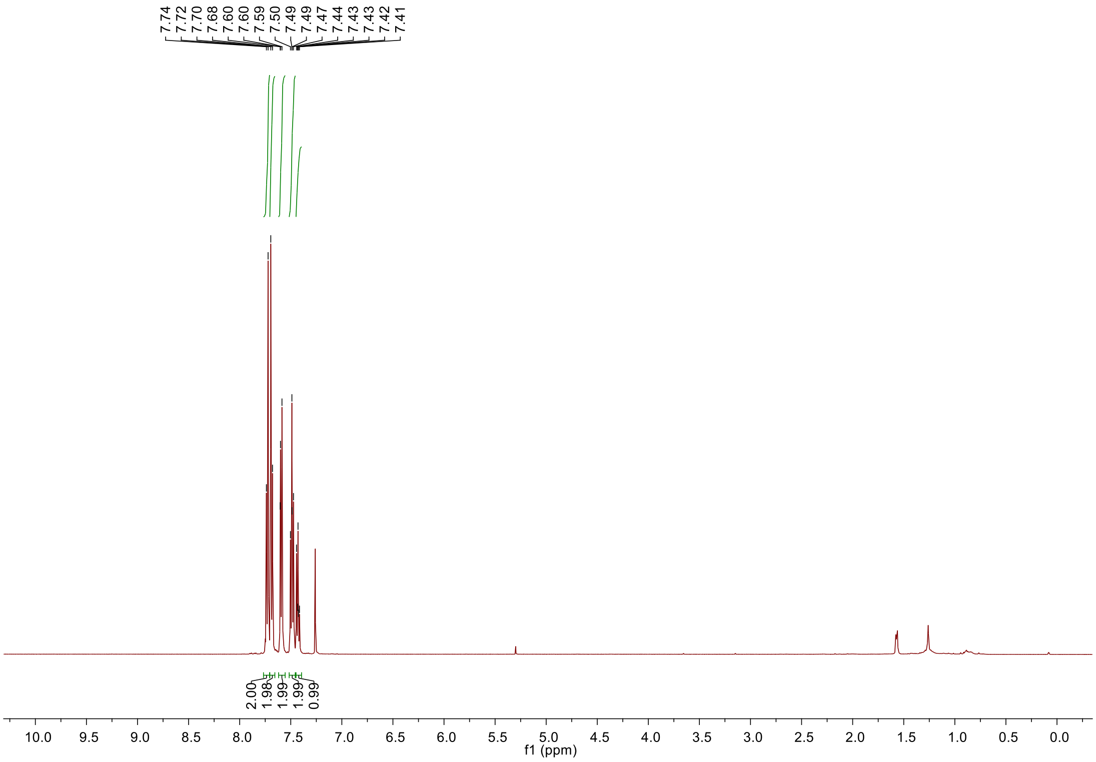
7.74
7.72
7.70
7.68
7.60
7.60
7.59
7.50
7.49
7.49
7.47
7.44
7.43
7.43
7.42
7.41
2.00
1.98
1.99
1.99
0.99
10.0 9.5 9.0 8.5 8.0 7.5 7.0 6.5 6.0 5.5 5.0 4.5 4.0 3.5 3.0 2.5 2.0 1.5 1.0 0.5 0.0
f1 (ppm)

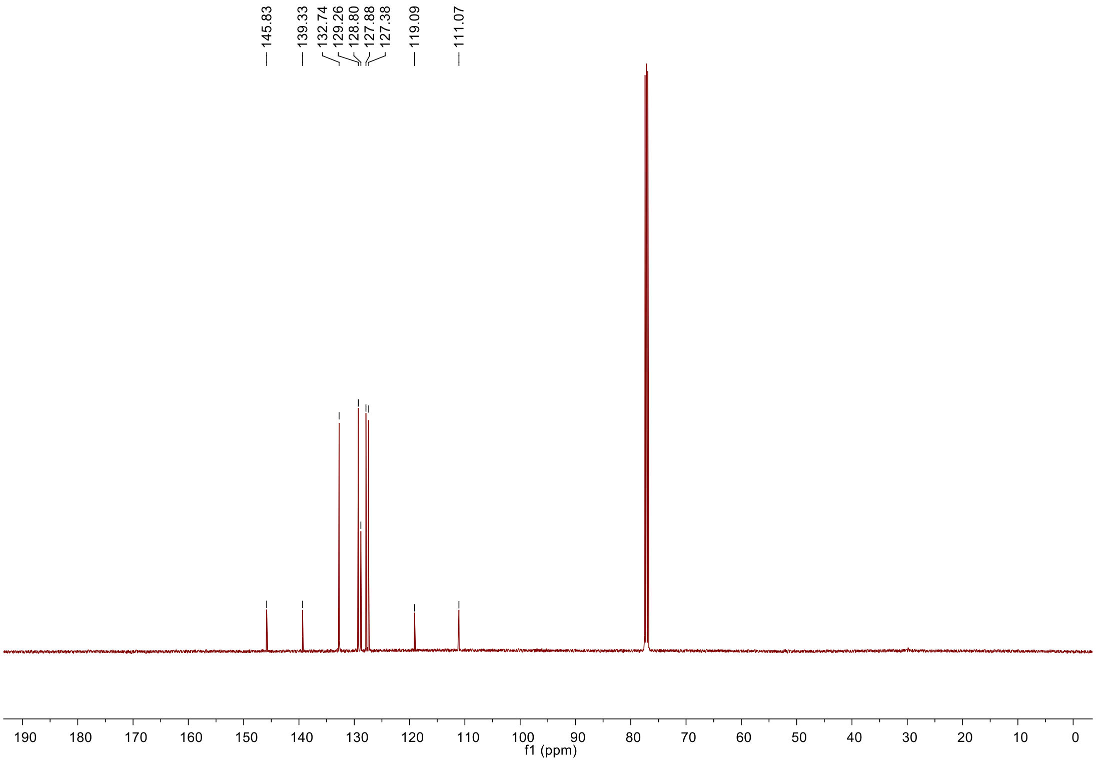
145.83
139.33
132.74
129.26
128.80
127.88
127.38
119.09
111.07
190 180 170 160 150 140 130 120 110 100 90 80 70 60 50 40 30 20 10 0
f1 (ppm)

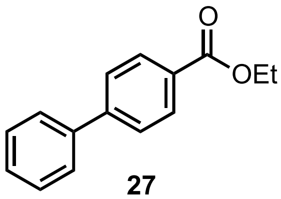

27

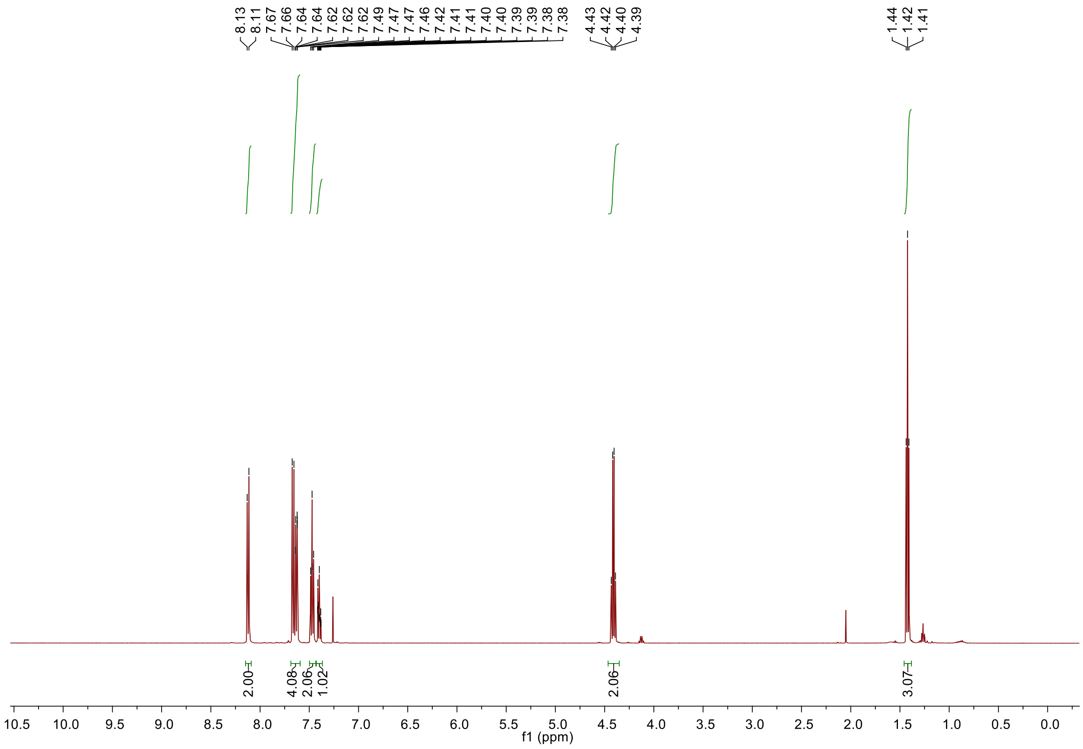
8.13
8.11
7.67
7.66
7.64
7.64
7.62
7.62
7.62
7.49
7.47
7.47
7.46
7.42
7.41
7.41
7.40
7.40
7.39
7.39
7.38
7.38
4.43
4.42
4.40
4.39
1.44
1.42
1.41
2.00
4.08
2.06
1.02
2.06
3.07
f1 (ppm)

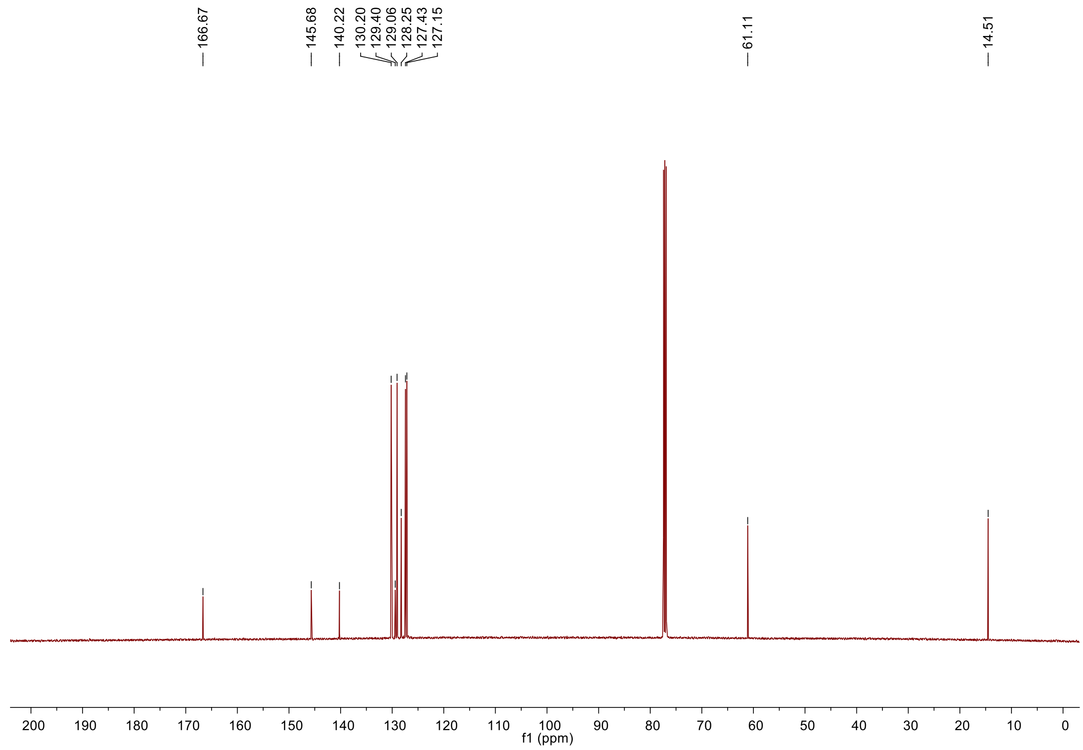
166.67
145.68
140.22
130.20
129.40
129.06
128.25
127.43
127.15
61.11
14.51
f1 (ppm)

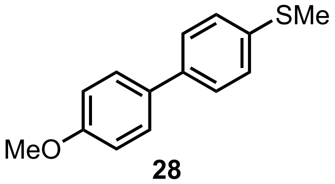
SMe
MeO

28

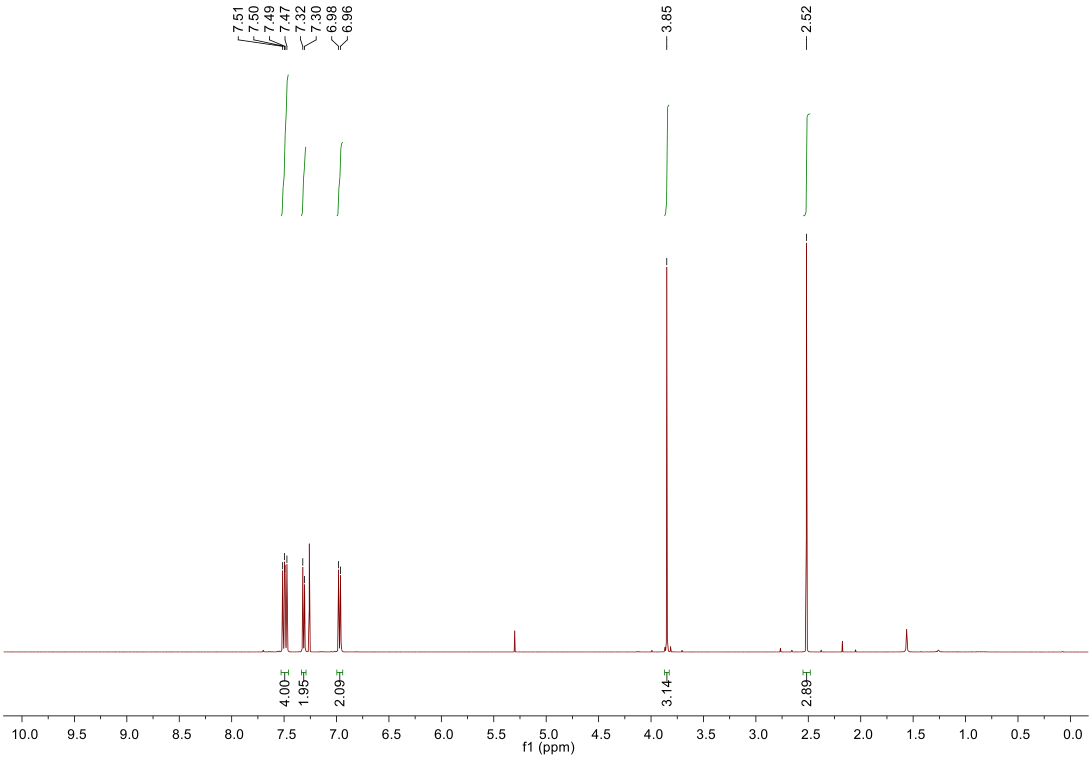
7.51
7.50
7.49
7.47
7.32
7.30
6.98
6.96
3.85
2.52
4.00
1.95
2.09
3.14
2.89
10.0 9.5 9.0 8.5 8.0 7.5 7.0 6.5 6.0 5.5 5.0 4.5 4.0 3.5 3.0 2.5 2.0 1.5 1.0 0.5 0.0
f1 (ppm)

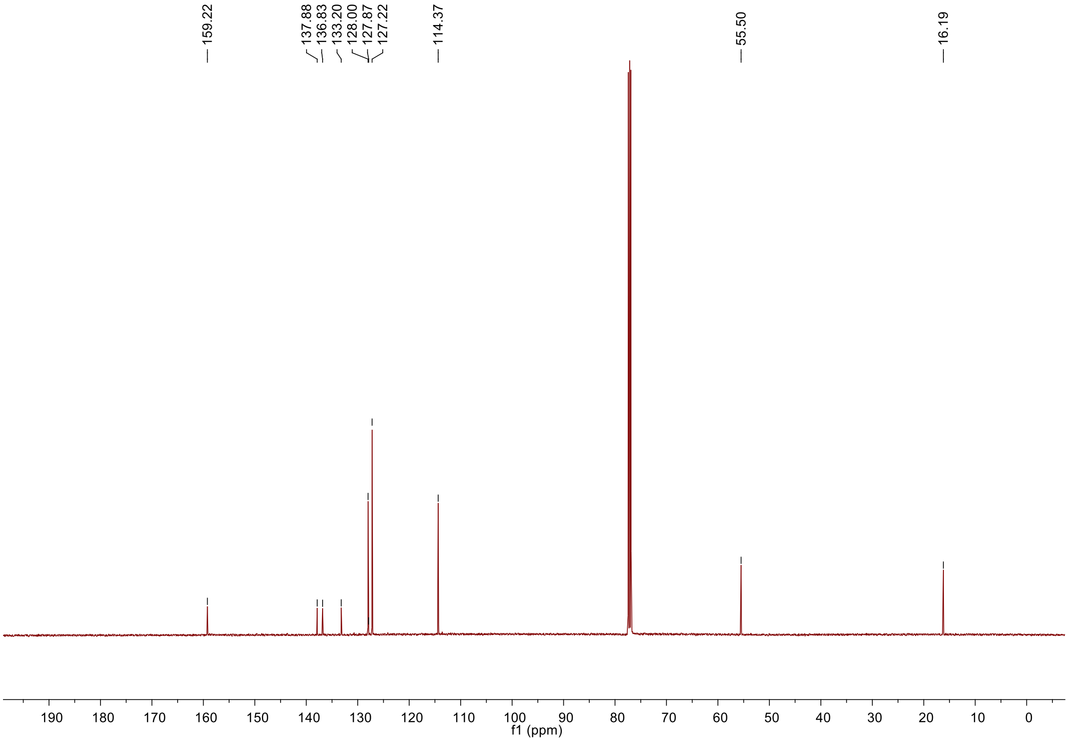
159.22
137.88
136.83
133.20
128.00
127.87
127.22
114.37
55.50
16.19
190 180 170 160 150 140 130 120 110 100 90 80 70 60 50 40 30 20 10 0
f1 (ppm)

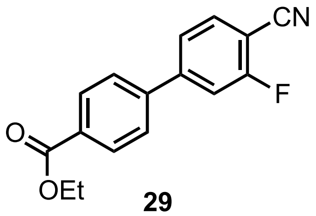

29

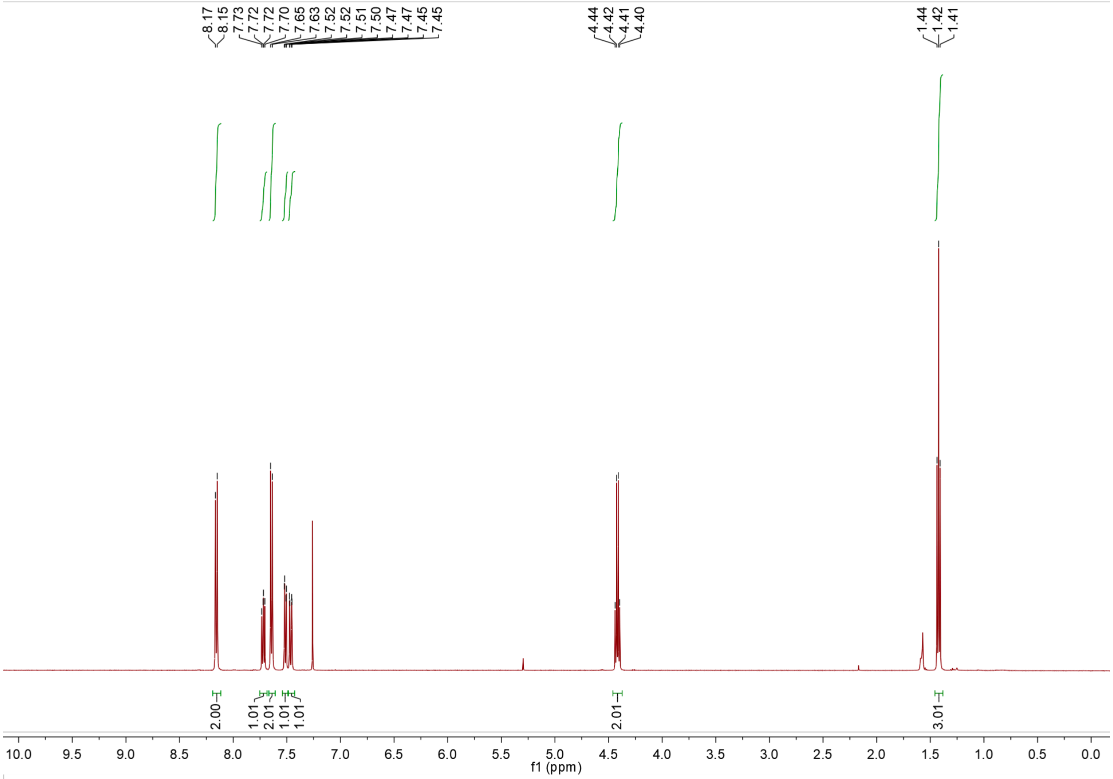

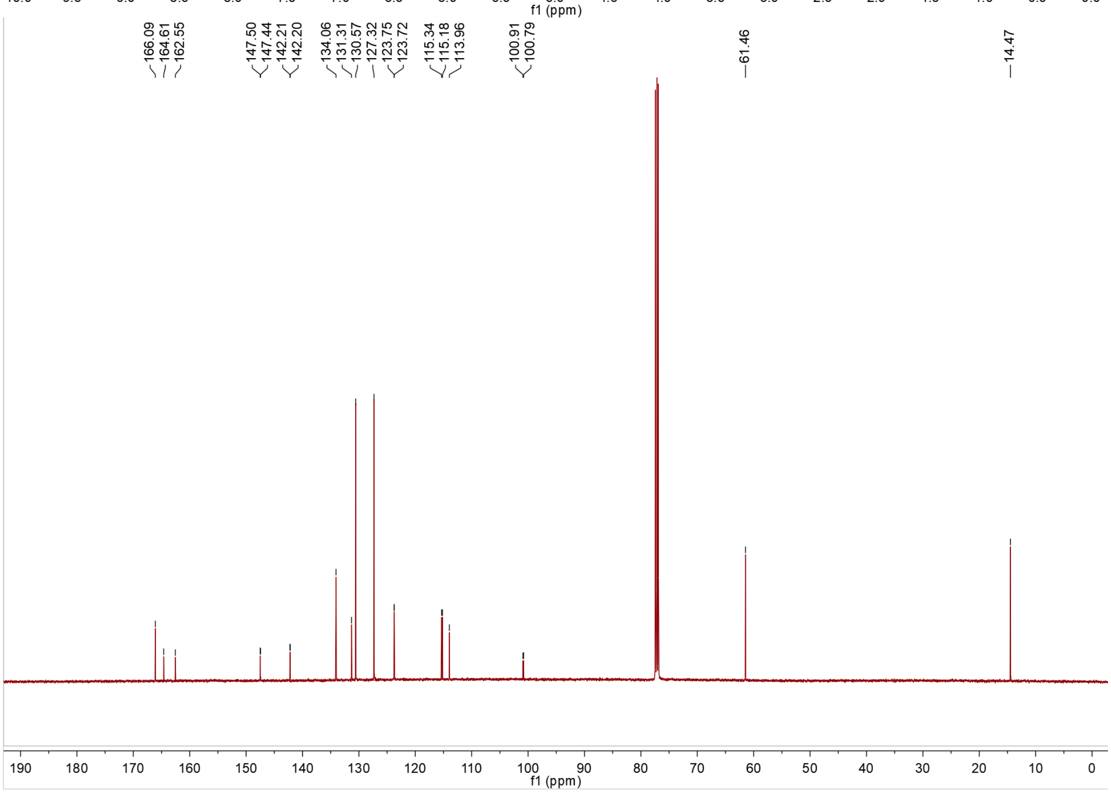

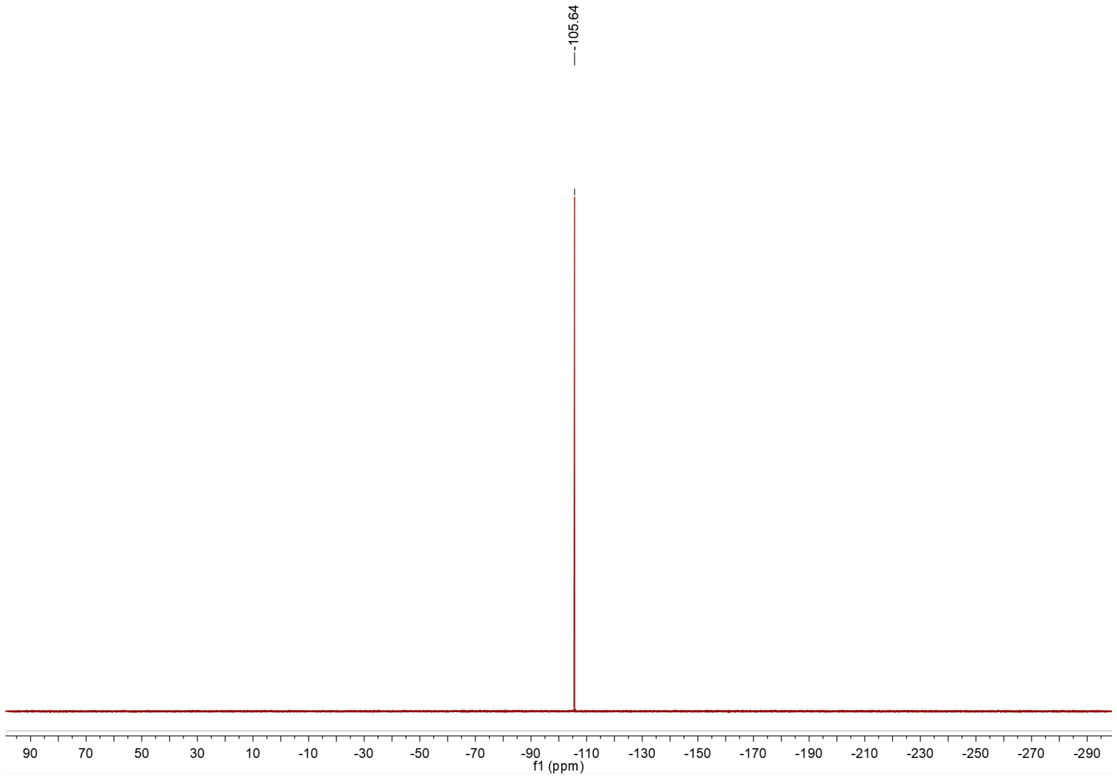
-105.64
90 70 50 30 10 -10 -30 -50 -70 -90 -110 -130 -150 -170 -190 -210 -230 -250 -270 -290
f1 (ppm)

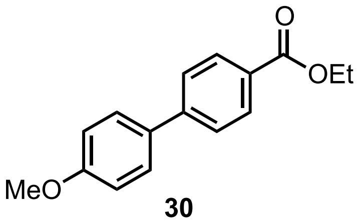

30

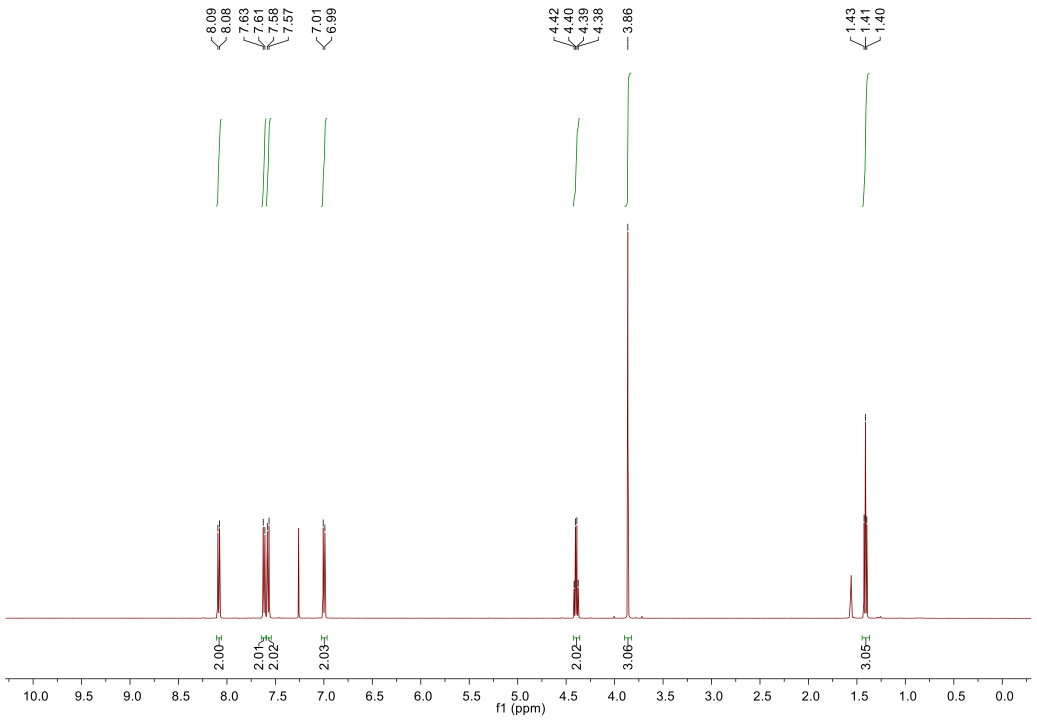

8.09
8.08
7.63
7.61
7.58
7.57
7.01
6.99
4.42
4.40
4.39
4.38
3.86
1.43
1.41
1.40
2.00
2.01
2.02
2.03
2.02
3.06
3.05
10.0 9.5 9.0 8.5 8.0 7.5 7.0 6.5 6.0 5.5 5.0 4.5 4.0 3.5 3.0 2.5 2.0 1.5 1.0 0.5 0.0
f1 (ppm)

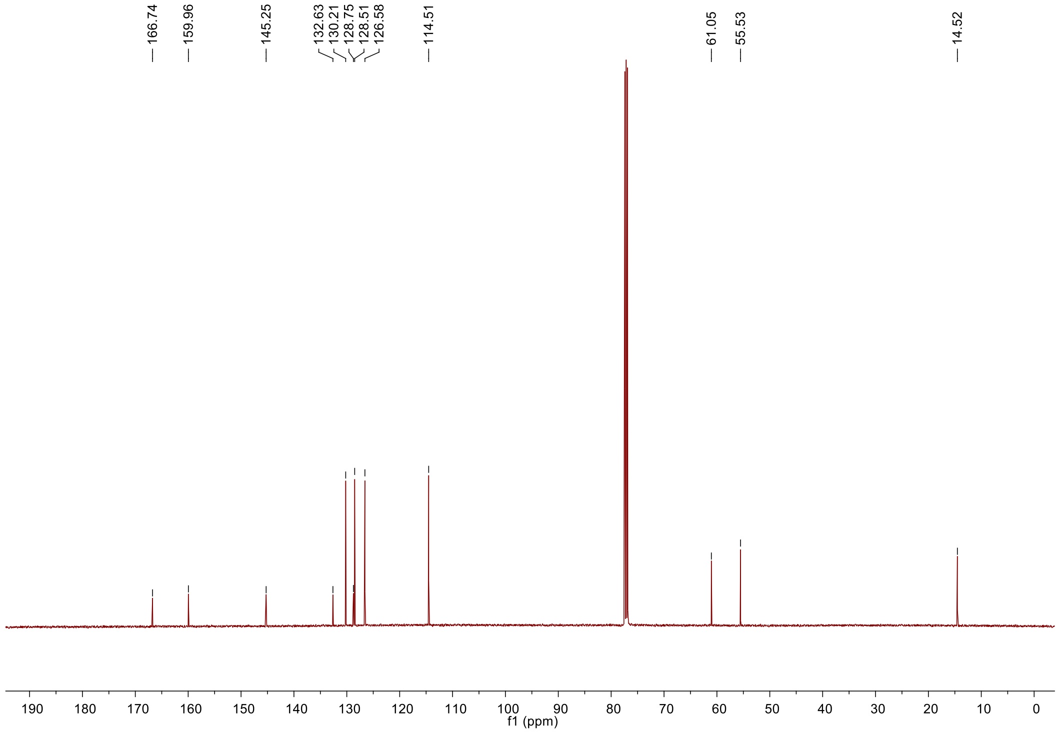

166.74
159.96
145.25
132.63
130.21
128.75
128.51
126.58
114.51
61.05
55.53
14.52
190 180 170 160 150 140 130 120 110 100 90 80 70 60 50 40 30 20 10 0
f1 (ppm)

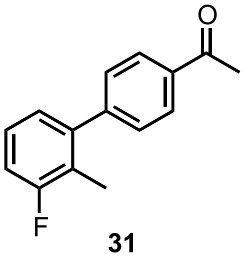

31

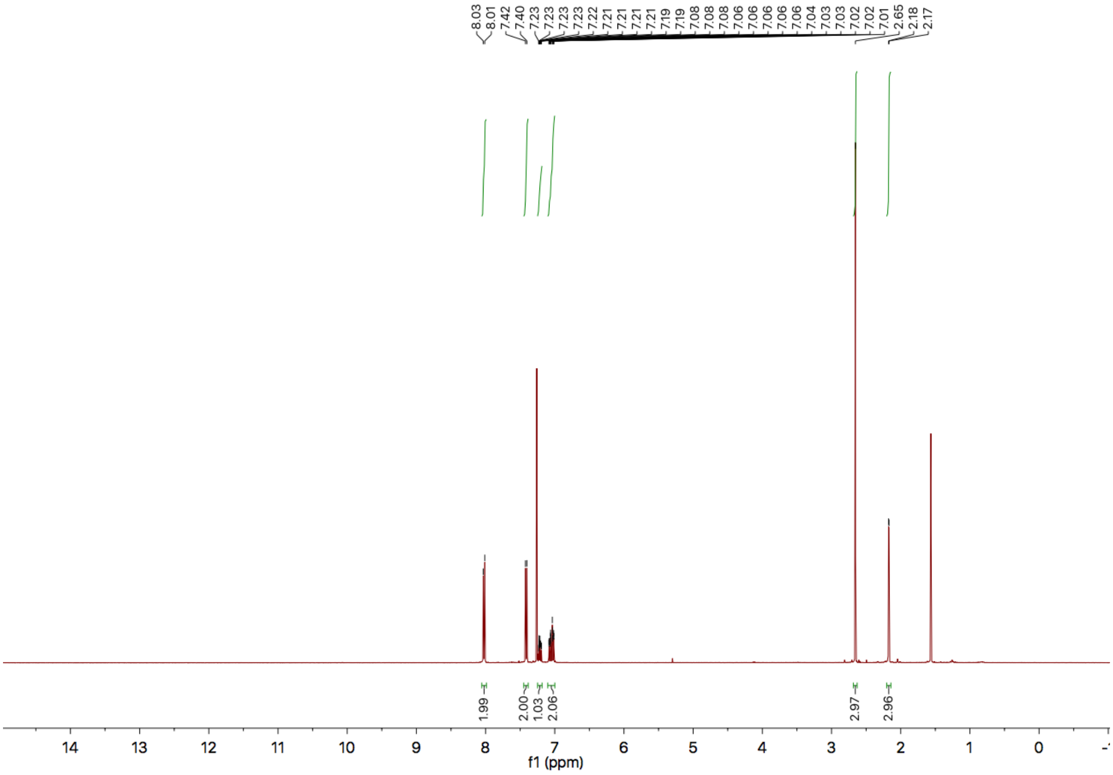
8.03
8.01
7.42
7.40
7.23
7.23
7.23
7.23
7.22
7.21
7.21
7.21
7.21
7.19
7.19
7.08
7.08
7.08
7.06
7.06
7.06
7.06
7.04
7.03
7.03
7.02
7.02
7.01
2.65
2.18
2.17
1.99
2.00
1.03
2.06
2.97
2.96
f1 (ppm)

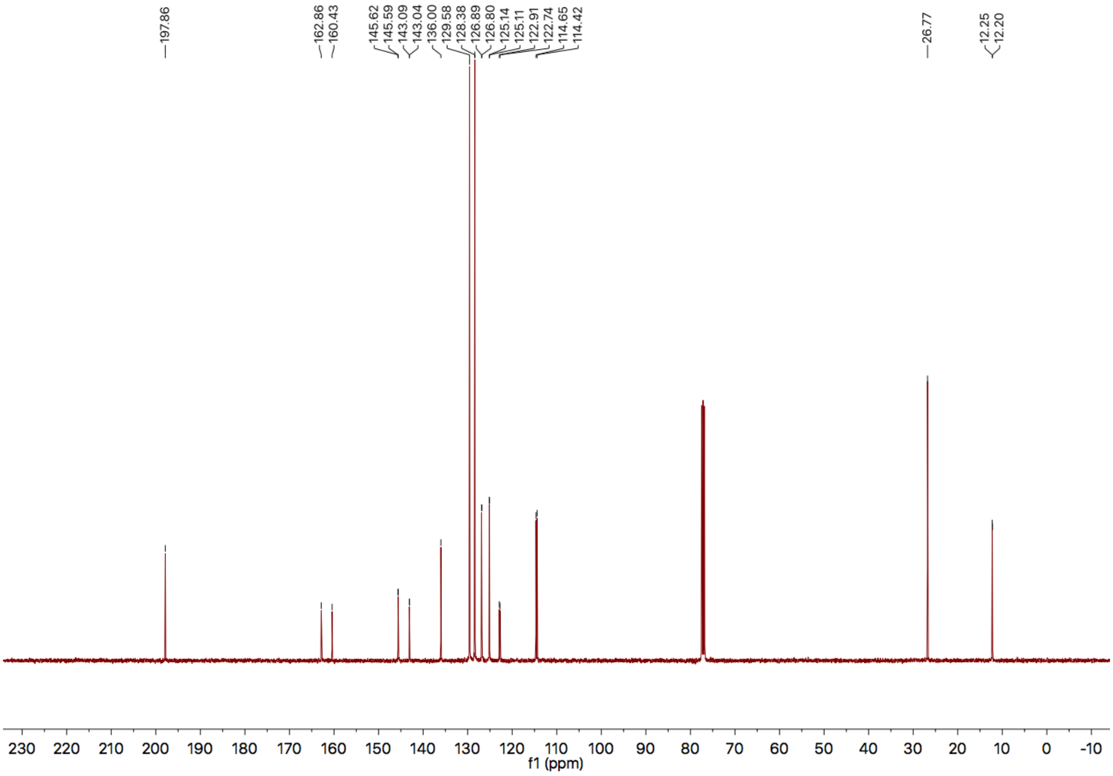
197.86
162.86
160.43
145.62
145.59
143.09
143.04
136.00
129.58
128.38
126.89
126.80
125.14
125.11
122.91
122.74
114.65
114.42
26.77
12.25
12.20
f1 (ppm)

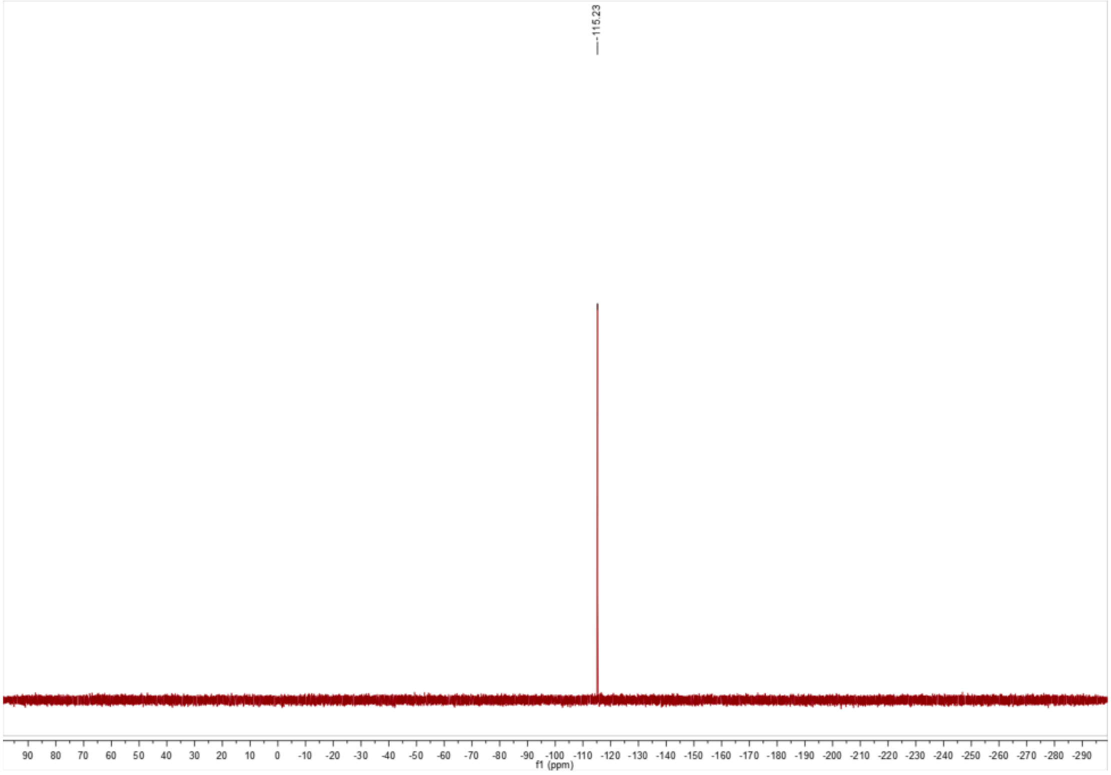
-115.23
90 80 70 60 50 40 30 20 10 0 -10 -20 -30 -40 -50 -60 -70 -80 -90 -100 -110 -120 -130 -140 -150 -160 -170 -180 -190 -200 -210 -220 -230 -240 -250 -260 -270 -280 -290
f1 (ppm)

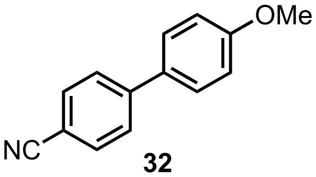
OMe
NC

32

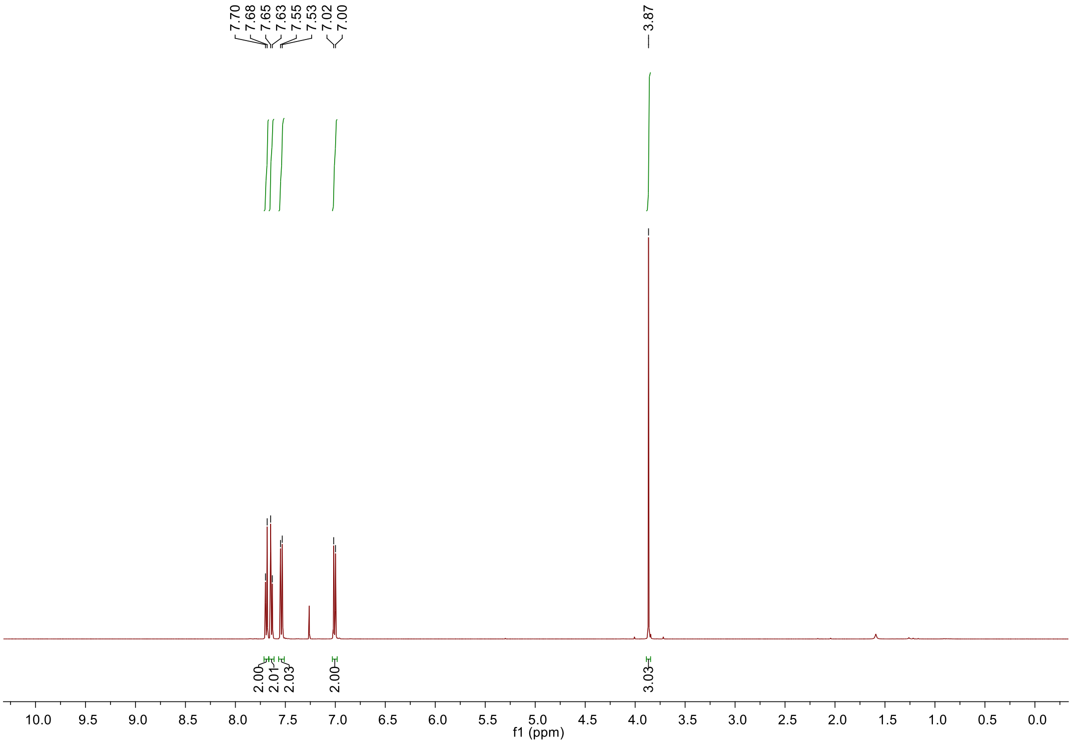
7.70
7.68
7.65
7.63
7.55
7.53
7.02
7.00
3.87
2.00
2.01
2.03
2.00
3.03
10.0 9.5 9.0 8.5 8.0 7.5 7.0 6.5 6.0 5.5 5.0 4.5 4.0 3.5 3.0 2.5 2.0 1.5 1.0 0.5 0.0
f1 (ppm)

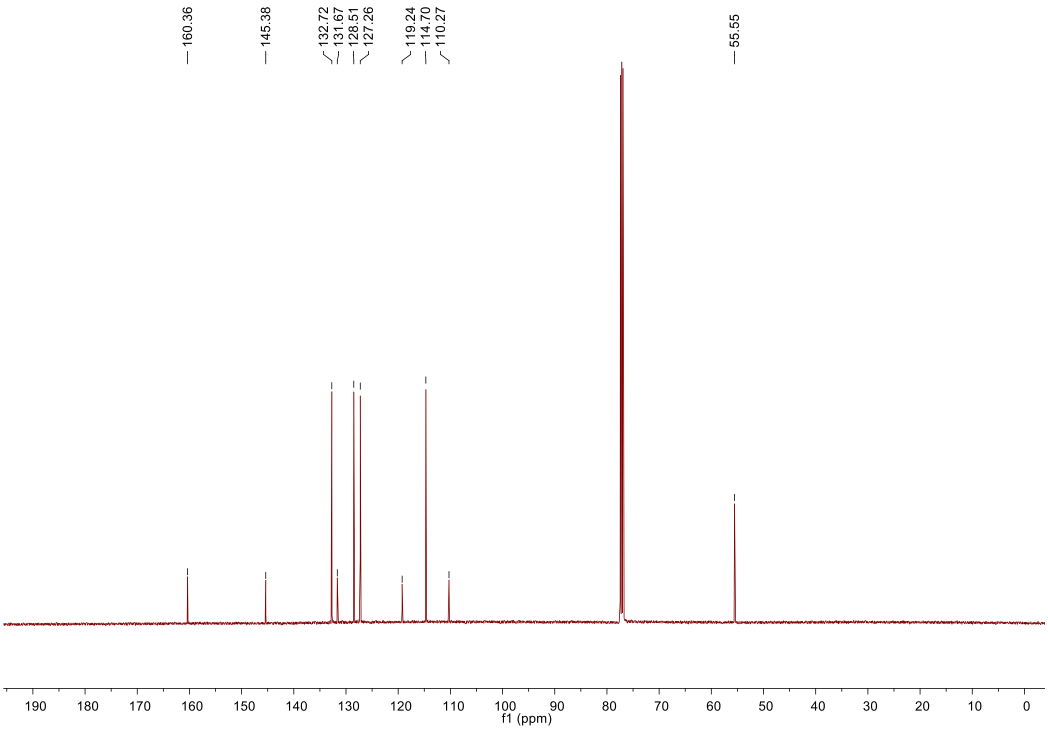
160.36
145.38
132.72
131.67
128.51
127.26
119.24
114.70
110.27
55.55
190 180 170 160 150 140 130 120 110 100 90 80 70 60 50 40 30 20 10 0
f1 (ppm)

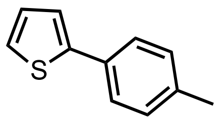

33

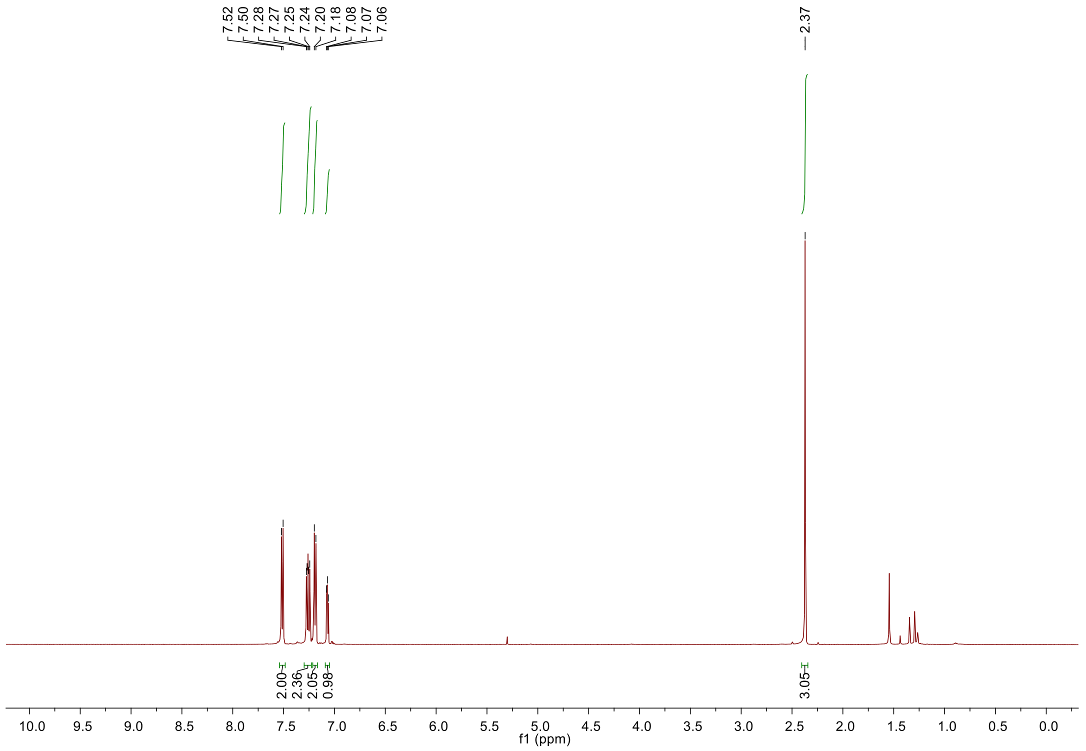

7.52
7.50
7.28
7.27
7.25
7.24
7.20
7.18
7.08
7.07
7.06
2.37
2.00
2.36
2.05
0.98
3.05
f1 (ppm)

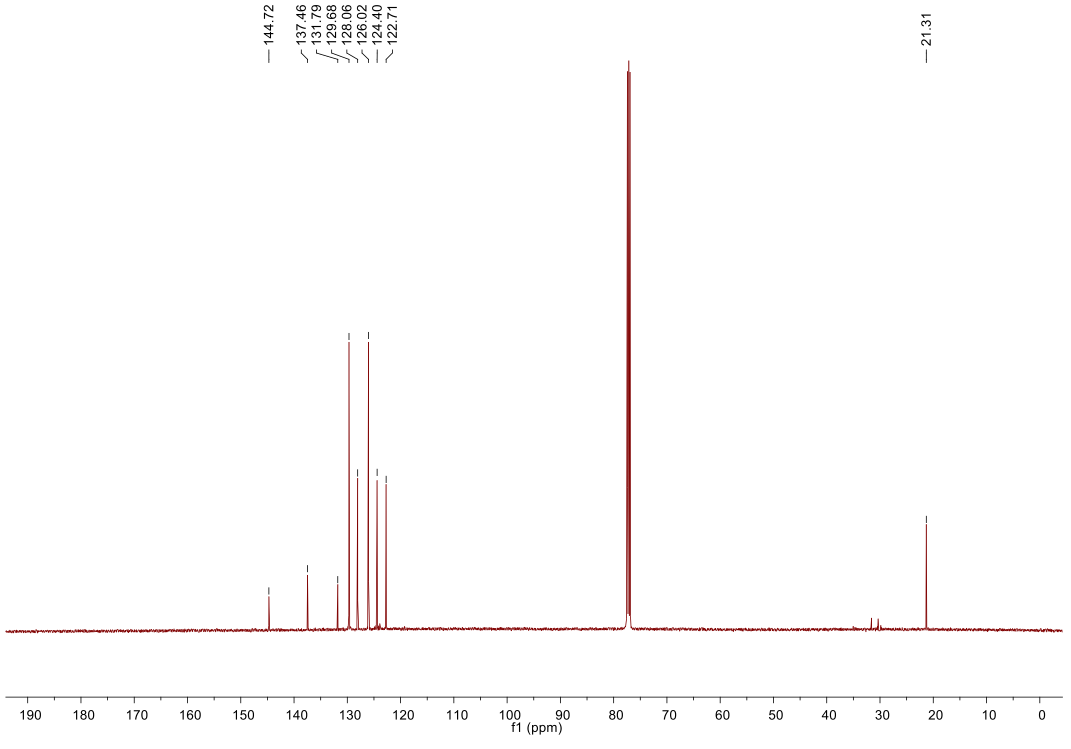

144.72
137.46
131.79
129.68
128.06
126.02
124.40
122.71
21.31
f1 (ppm)

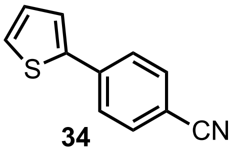
S
CN

34

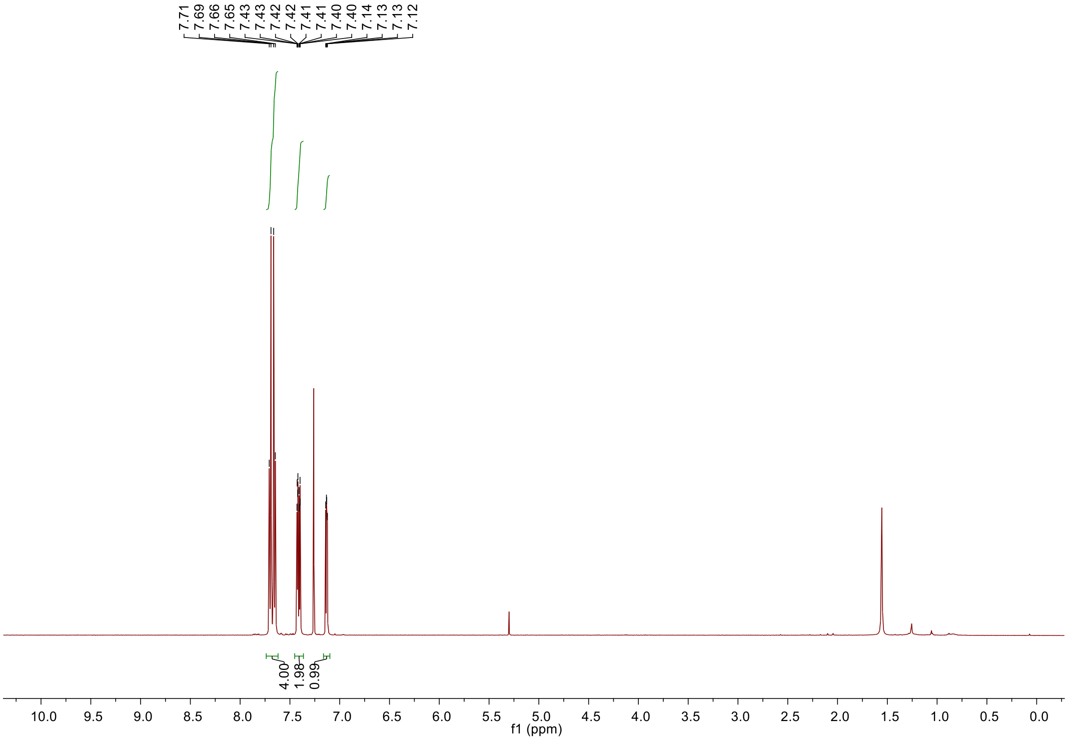
7.71
7.69
7.66
7.65
7.43
7.43
7.42
7.42
7.41
7.41
7.40
7.40
7.14
7.13
7.13
7.12
4.00
1.98
0.99
10.0 9.5 9.0 8.5 8.0 7.5 7.0 6.5 6.0 5.5 5.0 4.5 4.0 3.5 3.0 2.5 2.0 1.5 1.0 0.5 0.0
f1 (ppm)

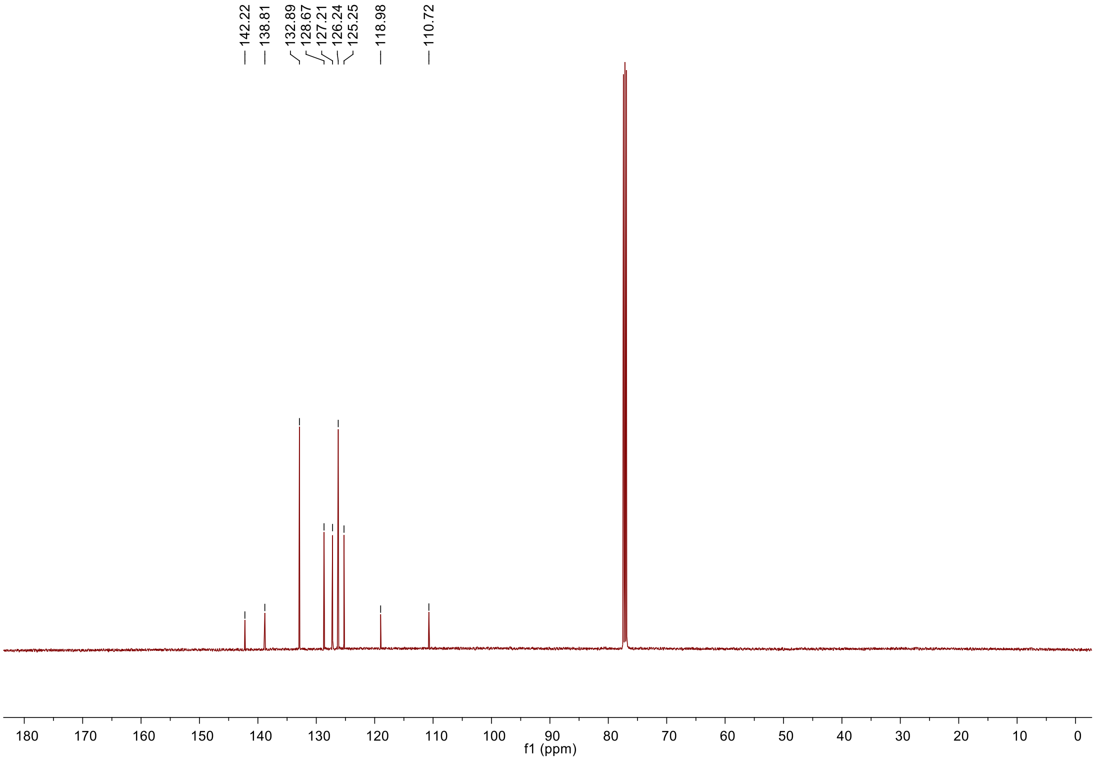
142.22
138.81
132.89
128.67
127.21
126.24
125.25
118.98
110.72
180 170 160 150 140 130 120 110 100 90 80 70 60 50 40 30 20 10 0
f1 (ppm)

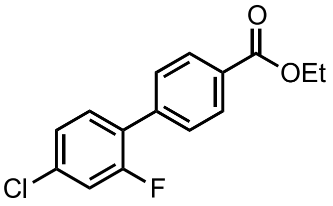
O
OEt
Cl
F

35

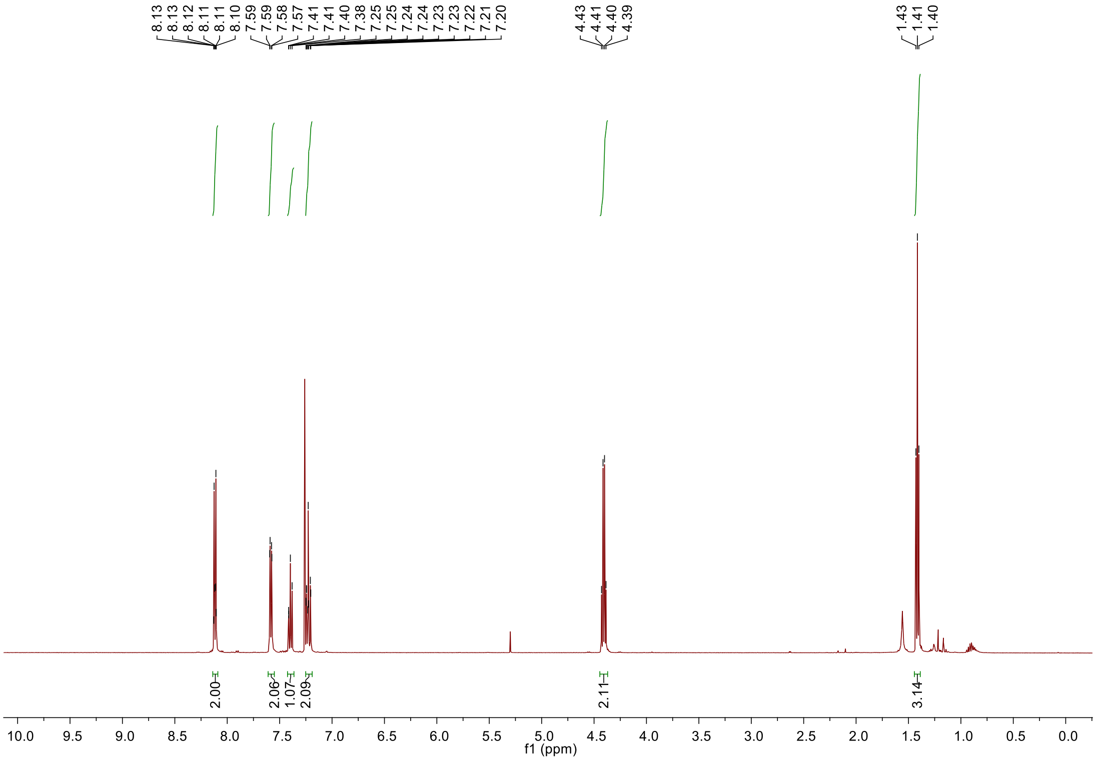
8.13
8.13
8.12
8.11
8.11
8.10
7.59
7.59
7.58
7.57
7.41
7.41
7.40
7.38
7.25
7.25
7.24
7.24
7.23
7.23
7.22
7.21
7.20
4.43
4.41
4.40
4.39
1.43
1.41
1.40
2.00
2.06
1.07
2.09
2.11
3.14
10.0 9.5 9.0 8.5 8.0 7.5 7.0 6.5 6.0 5.5 5.0 4.5 4.0 3.5 3.0 2.5 2.0 1.5 1.0 0.5 0.0
f1 (ppm)

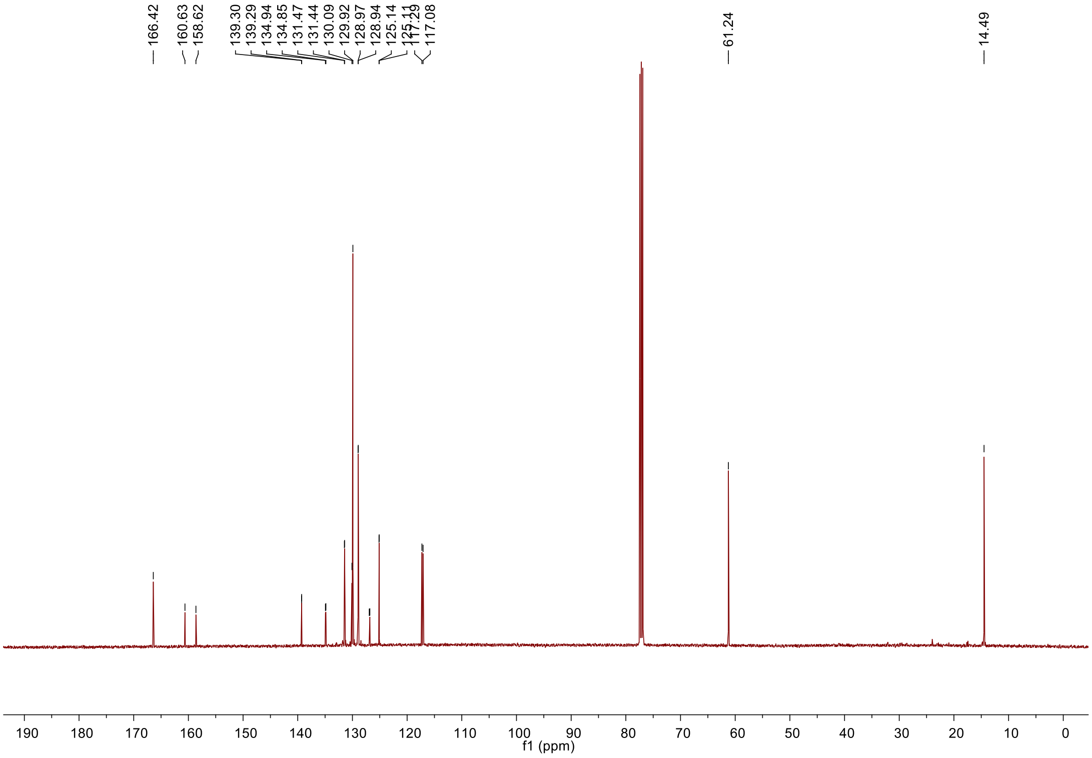
166.42
160.63
158.62
139.30
139.29
134.94
134.85
131.47
131.44
130.09
129.92
128.97
128.94
125.14
125.11
117.29
117.08
61.24
14.49
190 180 170 160 150 140 130 120 110 100 90 80 70 60 50 40 30 20 10 0
f1 (ppm)

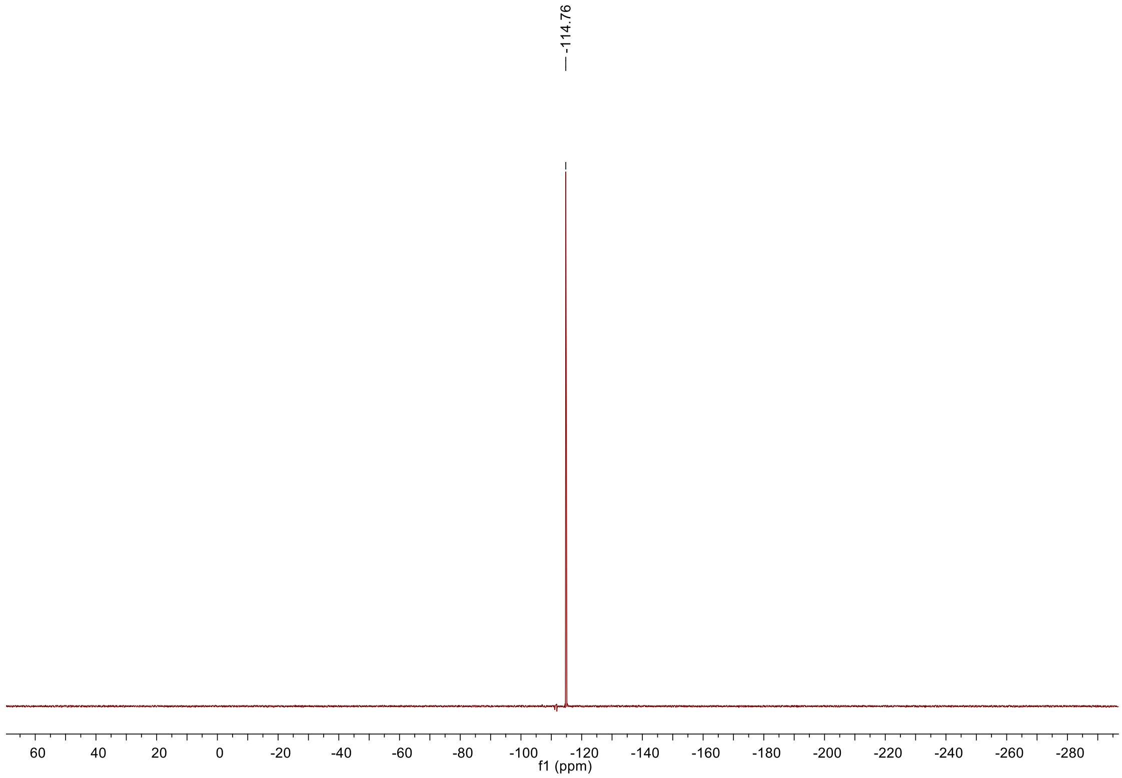
-114.76
60
40
20
0
-20
-40
-60
-80
-100
-120
-140
-160
-180
-200
-220
-240
-260
-280
f1 (ppm)

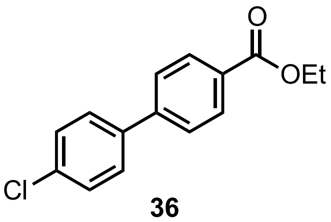
O
OEt
Cl

36

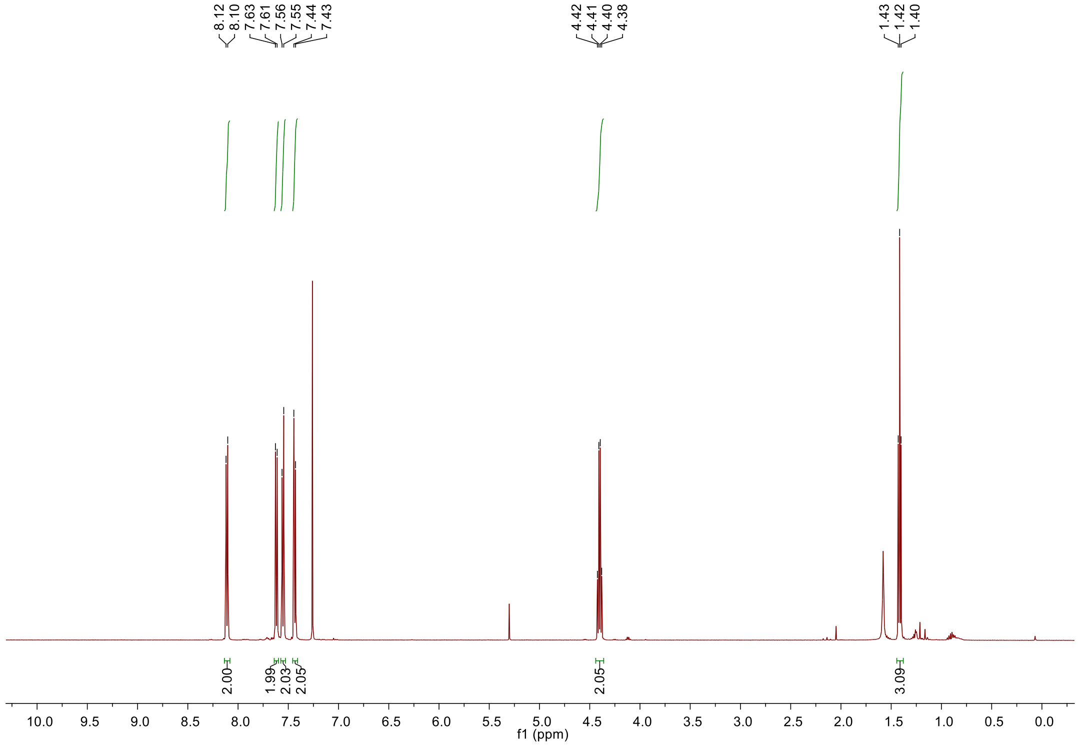
8.12
8.10
7.63
7.61
7.56
7.55
7.44
7.43
4.42
4.41
4.40
4.38
1.43
1.42
1.40
2.00
1.99
2.03
2.05
2.05
3.09
10.0 9.5 9.0 8.5 8.0 7.5 7.0 6.5 6.0 5.5 5.0 4.5 4.0 3.5 3.0 2.5 2.0 1.5 1.0 0.5 0.0
f1 (ppm)

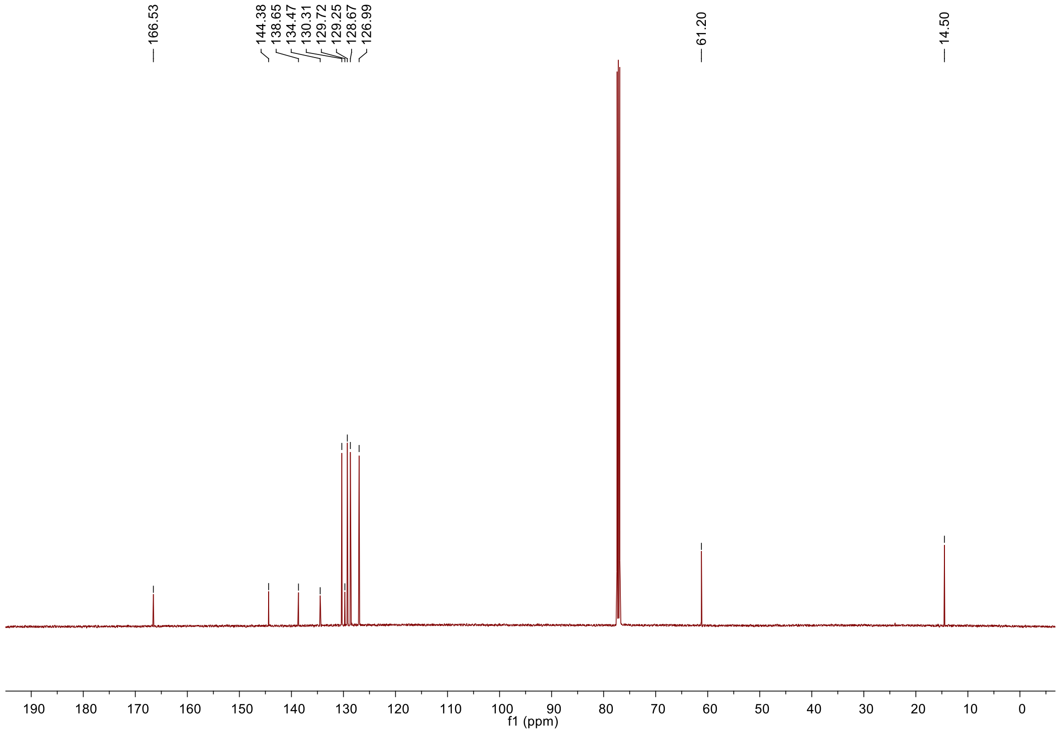
166.53
144.38
138.65
134.47
130.31
129.72
129.25
128.67
126.99
61.20
14.50
190 180 170 160 150 140 130 120 110 100 90 80 70 60 50 40 30 20 10 0
f1 (ppm)

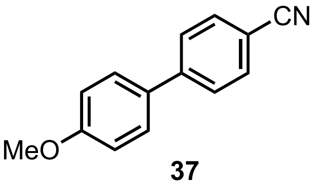

37

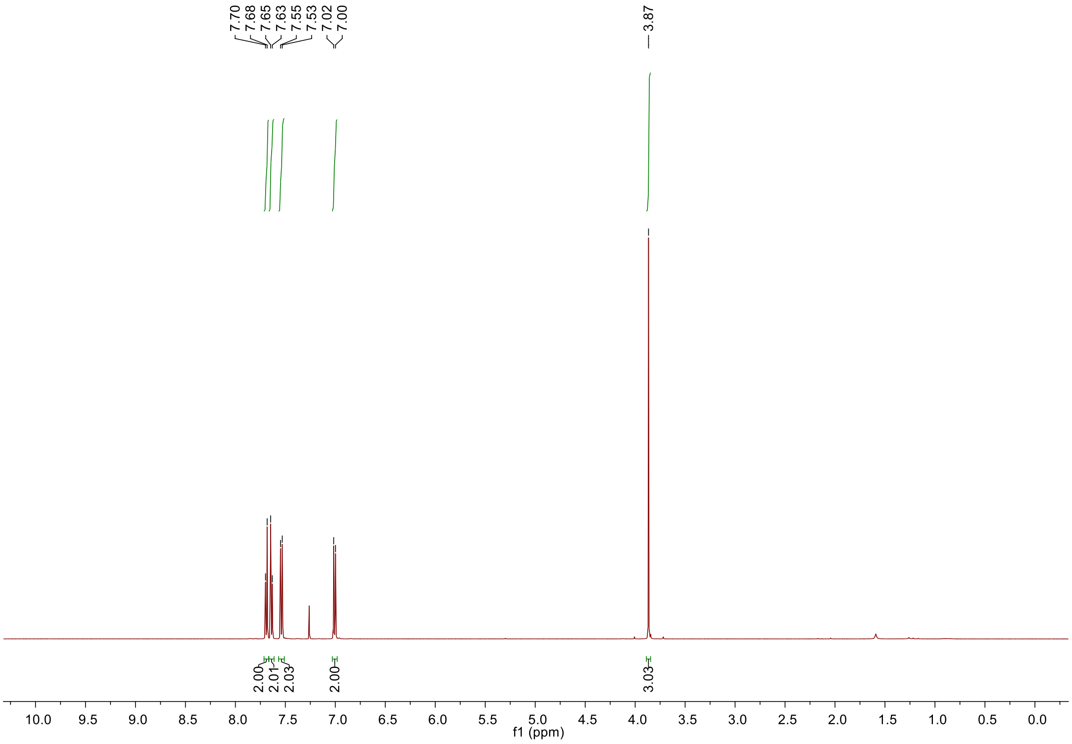
7.70
7.68
7.65
7.63
7.55
7.53
7.02
7.00
3.87
2.00
2.01
2.03
2.00
3.03
10.0 9.5 9.0 8.5 8.0 7.5 7.0 6.5 6.0 5.5 5.0 4.5 4.0 3.5 3.0 2.5 2.0 1.5 1.0 0.5 0.0
f1 (ppm)

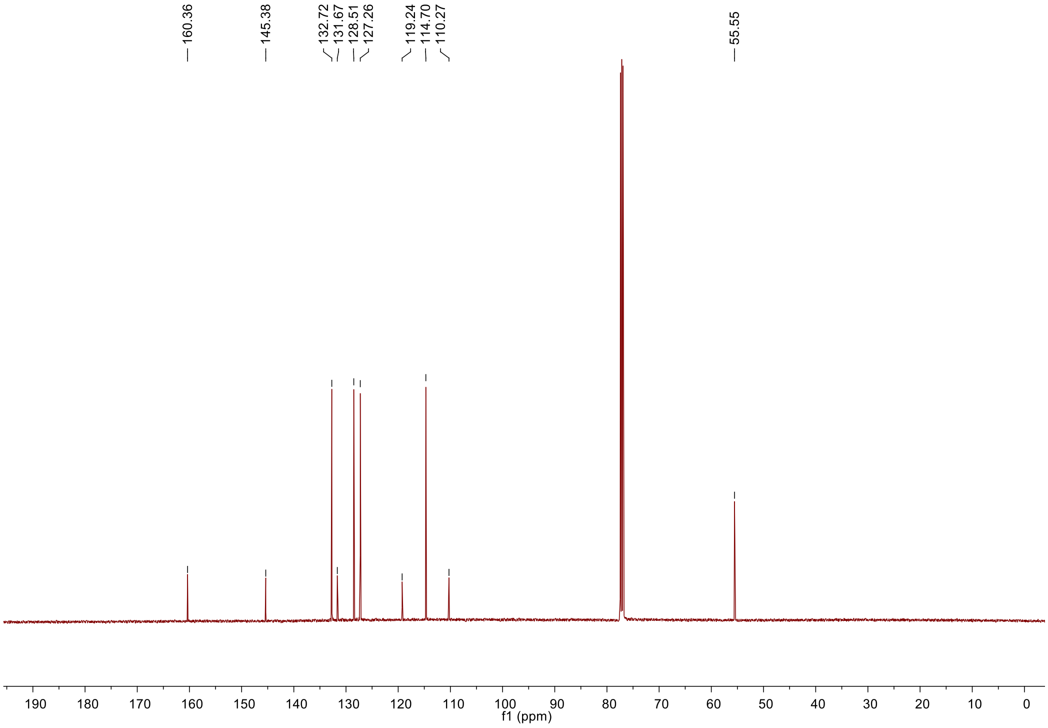
160.36
145.38
132.72
131.67
128.51
127.26
119.24
114.70
110.27
55.55
190 180 170 160 150 140 130 120 110 100 90 80 70 60 50 40 30 20 10 0
f1 (ppm)

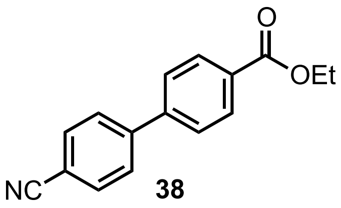
O
OEt
NC

38

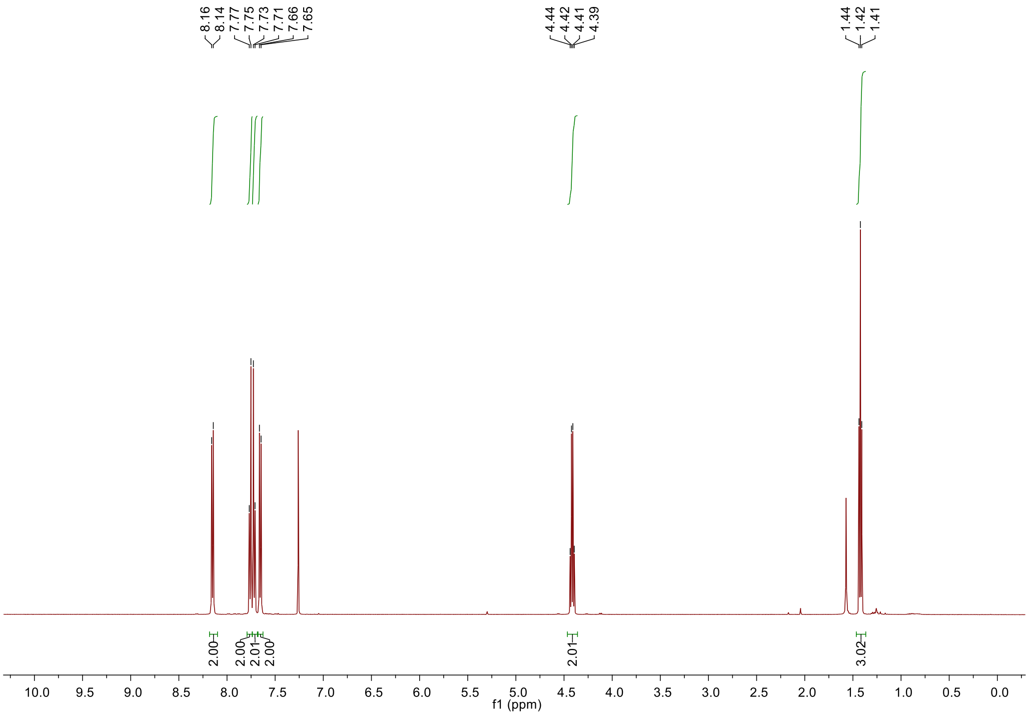
8.16
8.14
7.77
7.75
7.73
7.71
7.66
7.65
4.44
4.42
4.41
4.39
1.44
1.42
1.41
2.00
2.00
2.01
2.00
2.01
3.02
10.0 9.5 9.0 8.5 8.0 7.5 7.0 6.5 6.0 5.5 5.0 4.5 4.0 3.5 3.0 2.5 2.0 1.5 1.0 0.5 0.0
f1 (ppm)

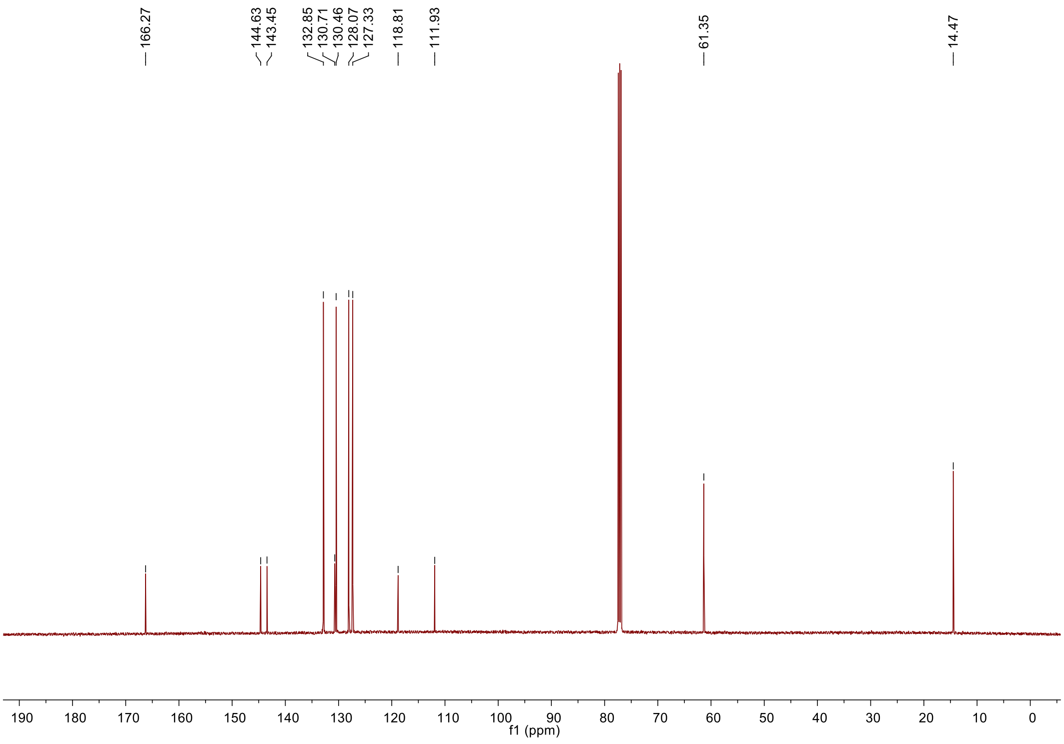
166.27
144.63
143.45
132.85
130.71
130.46
128.07
127.33
118.81
111.93
61.35
14.47
190 180 170 160 150 140 130 120 110 100 90 80 70 60 50 40 30 20 10 0
f1 (ppm)

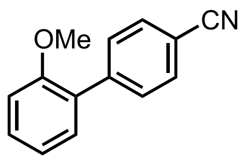
OMe
CN

39

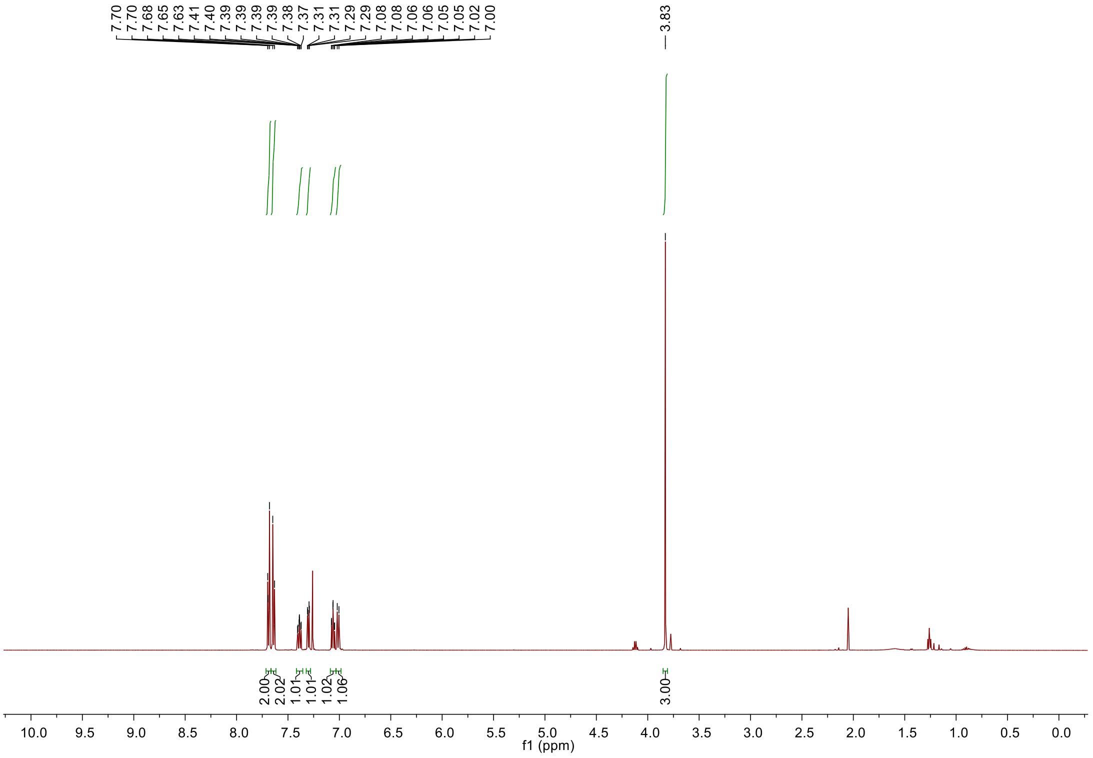
7.70
7.70
7.68
7.65
7.63
7.41
7.40
7.39
7.39
7.39
7.39
7.38
7.37
7.31
7.31
7.29
7.29
7.08
7.08
7.06
7.06
7.05
7.05
7.02
7.00
3.83
2.00
2.02
1.01
1.01
1.02
1.06
3.00
10.0 9.5 9.0 8.5 8.0 7.5 7.0 6.5 6.0 5.5 5.0 4.5 4.0 3.5 3.0 2.5 2.0 1.5 1.0 0.5 0.0
f1 (ppm)

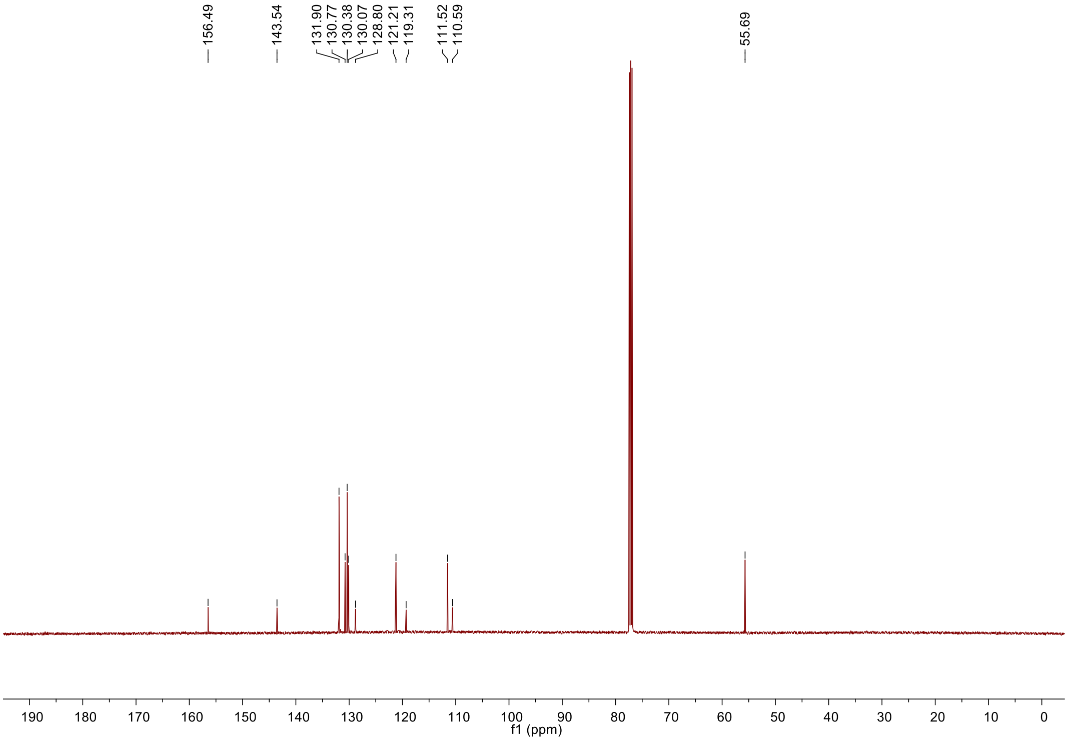
156.49
143.54
131.90
130.77
130.38
130.07
128.80
121.21
119.31
111.52
110.59
55.69
190 180 170 160 150 140 130 120 110 100 90 80 70 60 50 40 30 20 10 0
f1 (ppm)

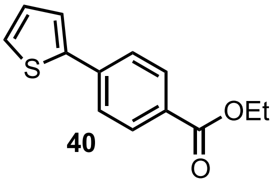

40

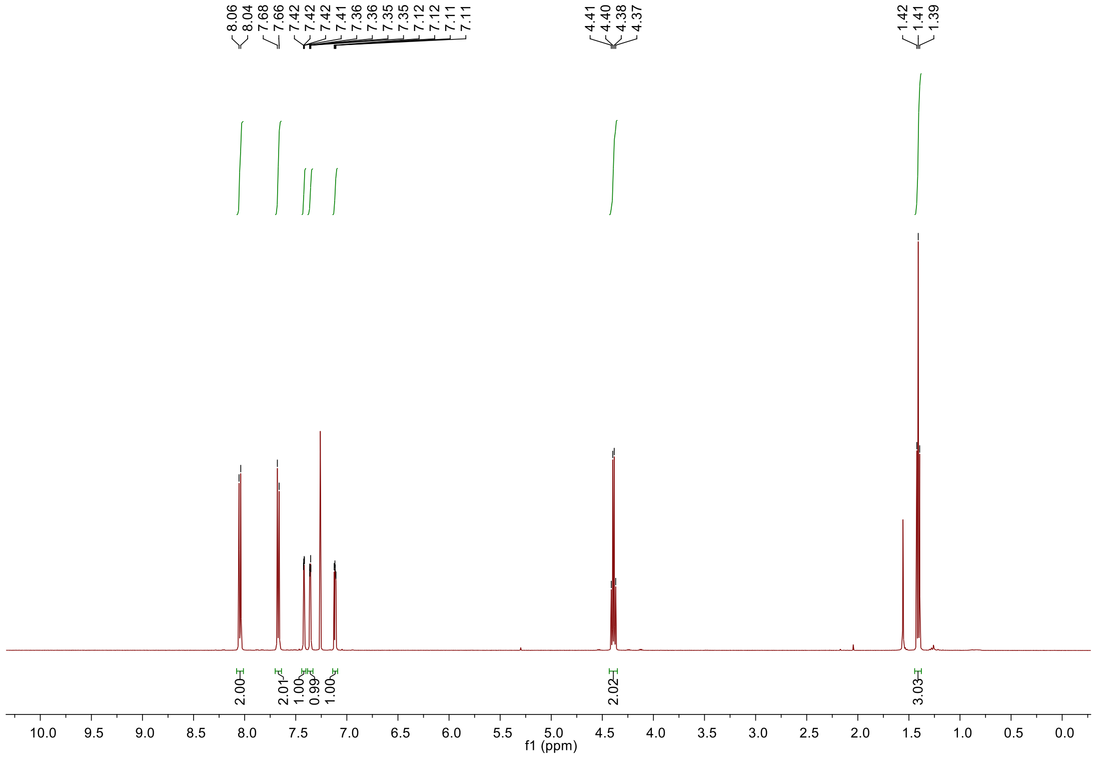

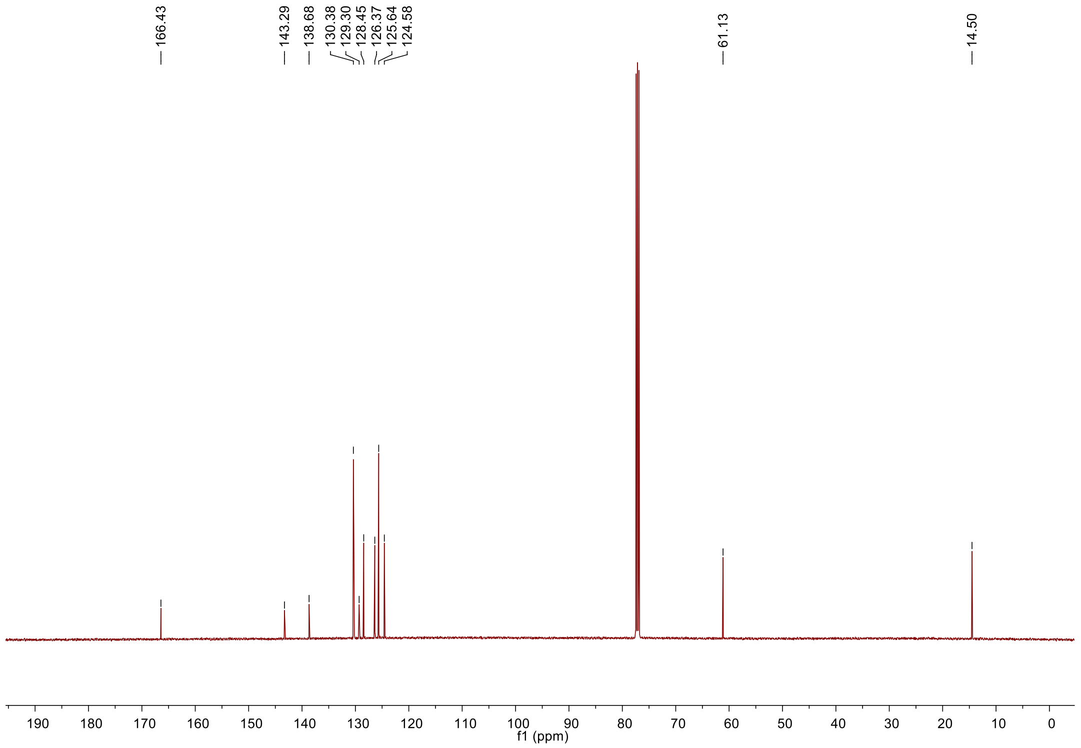

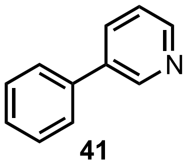

41

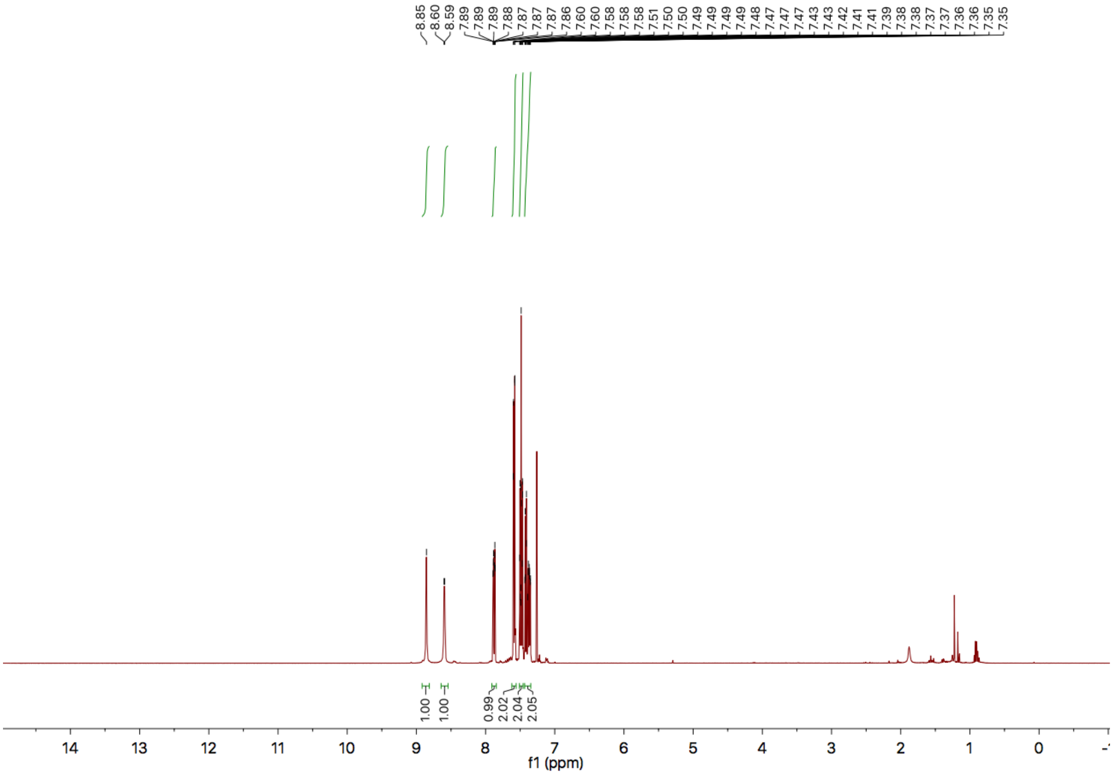

8.85
8.60
8.59
7.89
7.89
7.88
7.87
7.87
7.87
7.86
7.60
7.60
7.58
7.58
7.58
7.51
7.50
7.50
7.49
7.49
7.49
7.49
7.48
7.47
7.47
7.47
7.43
7.43
7.42
7.41
7.41
7.39
7.38
7.38
7.37
7.37
7.36
7.36
7.35
7.35
1.00
1.00
0.99
2.02
2.04
2.05
14
13
12
11
10
9
8
7
6
5
4
3
2
1
0
f1 (ppm)

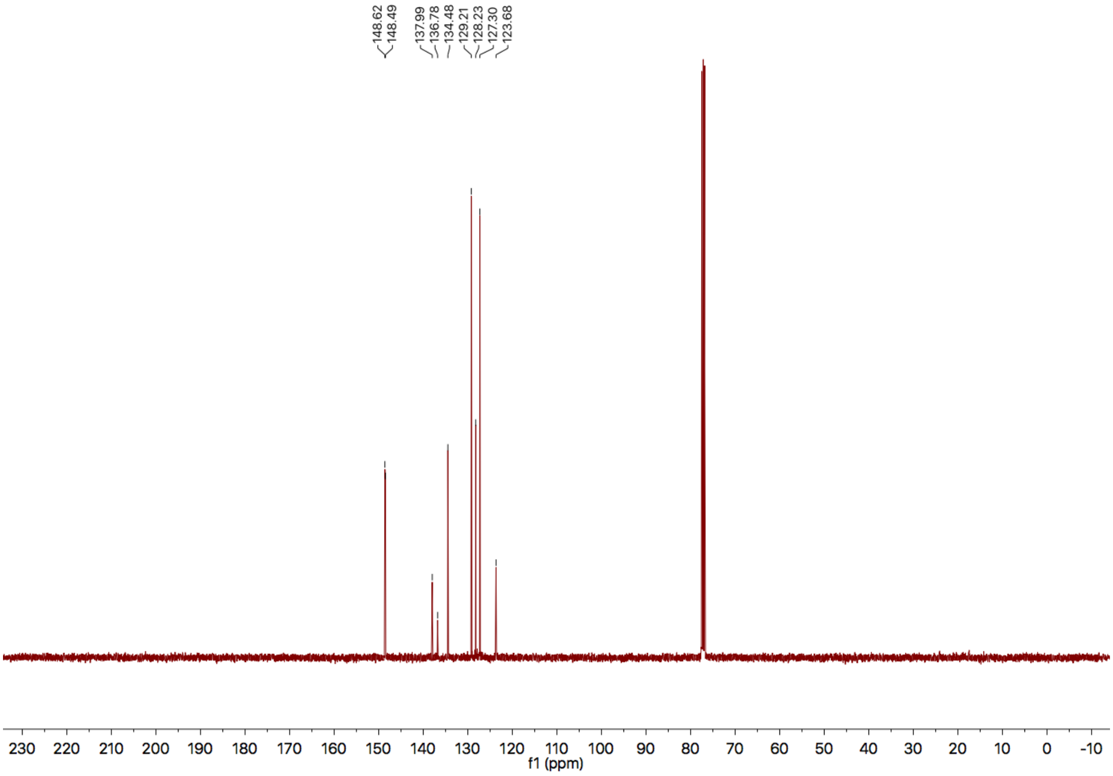

148.62
148.49
137.99
136.78
134.48
129.21
128.23
127.30
123.68
230
220
210
200
190
180
170
160
150
140
130
120
110
100
90
80
70
60
50
40
30
20
10
0
-10
f1 (ppm)

7 References

[1] Krasovskiy, A.; Knochel, P. *Synthesis.* **2006**, 5, 890.
[2] Huang, J.; Nolan, S. P. *J. Am. Chem. Soc.* **1999**, *121*, 9889.
[3] Vasudevan, K. V.; Butorac, R. R.; Abernethy, C. D.; Cowley, A. H. *Dalton Trans.* **2010**, *39*, 7401.
[4] O'Brien, C. J.; Kantchev, E. A. B.; Valente, C.; Hadei, N.; Chass, G. A.; Lough, A.; Hopkinson, A. C.; Organ, M. G. *Chem. Eur. J.* **2006**, *12*, 4743.
[5] Meiries, S.; Speck, K.; Cordes, D. B.; Slawin, A. M. Z.; Nolan, S. P. *Organometallics* **2013**, *32*, 330.
[6] Liu, Z.; Dong, N.; Xu, M.; Sun, Z.; Tu, T. *J. Org. Chem.* **2013**, *78*, 7436.
[7] Dastgir, S.; Coleman, K. S.; Cowley, A. R.; Green, M. L. H. *Organometallics* **2010**, *29*, 4858.
[8] Cao, Q.; Bailie, D. S.; Fu, R.; Muldoon, M. J. *Green Chem.* **2015**, *17*, 2750.
[9] Everson, D. A.; Shrestha, R.; Weix, D. J. *J. Am. Chem. Soc.* **2010**, *132*, 920.
[10] Chen, B.-Z.; Zhi, M.-L.; Wang, C.-X.; Chu, X.-Q.; Shen, Z.-L.; Loh, T.-P. *Org. Lett.* **2018**, 20, 1902
[11] Rasina, D.; Otikovs, M.; Leitans, J.; Recacha, R.; Borysov, O. V.; Kanepe-Lapsa, I.; Domraceva, I.; Pantelejevs, T.; Tars, K.; Blackman, M. J.; Jaudzems, K.; Jirgensons, A. *J. Med. Chem.* **2015**, *59*, 374.
[12] Liu, Z.; Dong, N.; Xu, M.; Sun, Z.; Tu, T. *J. Org. Chem.* **2013**, *78*, 7436.
[13] Basnet, P.; Thapa, S.; Dickie, D. A.; Giri, R. *Chem. Commun.* **2016**, *52*, 11072.
[14] Saphier, S.; Hu, Y.; Sinha, S. C.; Houk, K. N.; Keinan, E. *J. Am. Chem. Soc.* **2005**, *127*, 132.
[15] Whitaker, L.; Harb, H. Y.; Pulis, A. P. *Chem. Commun.* **2017**, *53*, 9364.
[16] Mäsing, F.; Nüsse, H.; Klingauf, J.; Studer, A. *Org. Lett.* **2018**, *20*, 752.
[17] Otsuka, S.; Fujino, D.; Murakami, K.; Yorimitsu, H.; Osuka, A. *Chem. Eur. J.* **2014**, *20*, 13146.
[18] Haag, B. A.; Sämann, C.; Jana, A.; Knochel, P. *Angew. Chem. Int. Ed.* **2011**, *50*, 7290.
[19] Liang, Q.; Xing, P.; Huang, Z.; Dong, J.; Sharpless, K. B.; Li, X.; Jiang, B. *Org. Lett.* **2015**, *17*, 1942.
[20] Shi, S.; Szostak, M. *Org. Lett.* **2017**, *19*, 3095.
[21] Vila, C.; Hornillos, V.; Giannerini, M.; Fañanás-Mastral, M.; Feringa, B. L. *Chem. Eur. J.* **2014**, *20*, 13078.
[22] Sévigny, S.; Forgione, P. *Chem. Eur. J.* **2013**, *19*, 2256.
[23] Ueura, K.; Satoh, T.; Miura, M. *Org. Lett.* **2005**, *7*, 2229.
[24] Sase, S.; Jaric, M.; Metzger, A.; Malakhov, V.; Knochel, P. *J. Org. Chem.* **2008**, *73*, 7380.
[25] Papoian, V.; Minehan, T. *J. Org. Chem.* **2008**, *73*, 7376.
[26] Ackermann, L.; Gschrei, C. J.; Althammer, A.; Riederer, M. *Chem. Commun.* **2006**, *41*, 1419.
